# Self-monitoring of blood pressure in hypertension: A systematic review and individual patient data meta-analysis

**DOI:** 10.1371/journal.pmed.1002389

**Published:** 2017-09-19

**Authors:** Katherine L. Tucker, James P. Sheppard, Richard Stevens, Hayden B. Bosworth, Alfred Bove, Emma P. Bray, Kenneth Earle, Johnson George, Marshall Godwin, Beverly B. Green, Paul Hebert, F. D. Richard Hobbs, Ilkka Kantola, Sally M. Kerry, Alfonso Leiva, David J. Magid, Jonathan Mant, Karen L. Margolis, Brian McKinstry, Mary Ann McLaughlin, Stefano Omboni, Olugbenga Ogedegbe, Gianfranco Parati, Nashat Qamar, Bahman P. Tabaei, Juha Varis, Willem J. Verberk, Bonnie J. Wakefield, Richard J. McManus

**Affiliations:** 1 Nuffield Department of Primary Care, University of Oxford, Oxford, United Kingdom; 2 Center for Health Services Research in Primary Care, Durham VAMC, Durham, North Carolina, United States of America; 3 Cardiology, Lewis Katz School of Medicine, Temple University, Philadelphia, Pennsylvania, United States of America; 4 School of Psychology, University of Central Lancashire, Preston, United Kingdom; 5 Thomas Addison Diabetes Unit, St. George's NHS Trust, London, United Kingdom; 6 Centre for Medicine Use and Safety, Faculty of Pharmacy and Pharmaceutical Sciences, Monash University, Parkville, Australia; 7 Family Medicine, Memorial University of Newfoundland, St. John’s, Canada; 8 Kaiser Permanente Washington Health Research Institute, Seattle, Washington, United States of America; 9 Department of Health Services, University of Washington School of Public Health, Seattle, Washington, United States of America; 10 Division of Medicine, Turku University Hospital and University of Turku, Turku, Finland; 11 Centre for Primary Care and Public Health, Queen Mary University of London, London, United Kingdom; 12 Primary Care Research Unit of Mallorca, Baleares Health Services-IbSalut, Mallorca, Spain; 13 Colorado School of Public Health, University of Colorado, Denver, Colorado, United States of America; 14 Primary Care Unit, Department of Public Health and Primary Care, University of Cambridge, Cambridge, United Kingdom; 15 HealthPartners Institute for Education and Research, Minneapolis, Minnesota, United States of America; 16 Usher Institute of Population Health Sciences and Informatics, University of Edinburgh, Edinburgh, United Kingdom; 17 Icahn School of Medicine at Mount Sinai New York, New York, New York, United States of America; 18 Clinical Research Unit, Italian Institute of Telemedicine, Varese, Italy; 19 Center for Healthful Behavior Change, Division of Health and Behavior, Department of Population Health, Langone School of Medicine, New York University, New York, New York, United States of America; 20 Department of Cardiovascular, Neural and Metabolic Sciences, IRCCS, San Luca Hospital, Istituto Auxologico Italiano, Milan, Italy; 21 Department of Medicine and Surgery, University of Milano-Bicocca, Milan, Italy; 22 Primary Care Clinical Sciences, University of Birmingham, Birmingham, United Kingdom; 23 Division of Prevention and Primary Care, New York City Department of Health & Mental Hygiene, New York, New York, United States of America; 24 Cardiovascular Research Institute Maastricht and Departments of Internal Medicine, Maastricht University, Maastricht, the Netherlands; 25 Department of Veterans (VA) Health Services Research and Development Centre for Comprehensive Access and Delivery Research and Evaluation (CADRE), Iowa City VA Medical Centre, University of Iowa, Iowa, United States of America; University of Oxford, UNITED KINGDOM

## Abstract

**Background:**

Self-monitoring of blood pressure (BP) appears to reduce BP in hypertension but important questions remain regarding effective implementation and which groups may benefit most. This individual patient data (IPD) meta-analysis was performed to better understand the effectiveness of BP self-monitoring to lower BP and control hypertension.

**Methods and findings:**

Medline, Embase, and the Cochrane Library were searched for randomised trials comparing self-monitoring to no self-monitoring in hypertensive patients (June 2016). Two reviewers independently assessed articles for eligibility and the authors of eligible trials were approached requesting IPD. Of 2,846 articles in the initial search, 36 were eligible. IPD were provided from 25 trials, including 1 unpublished study. Data for the primary outcomes—change in mean clinic or ambulatory BP and proportion controlled below target at 12 months—were available from 15/19 possible studies (7,138/8,292 [86%] of randomised participants). Overall, self-monitoring was associated with reduced clinic systolic blood pressure (sBP) compared to usual care at 12 months (−3.2 mmHg, [95% CI −4.9, −1.6 mmHg]). However, this effect was strongly influenced by the intensity of co-intervention ranging from no effect with self-monitoring alone (−1.0 mmHg [−3.3, 1.2]), to a 6.1 mmHg (−9.0, −3.2) reduction when monitoring was combined with intensive support. Self-monitoring was most effective in those with fewer antihypertensive medications and higher baseline sBP up to 170 mmHg. No differences in efficacy were seen by sex or by most comorbidities. Ambulatory BP data at 12 months were available from 4 trials (1,478 patients), which assessed self-monitoring with little or no co-intervention. There was no association between self-monitoring and either lower clinic or ambulatory sBP in this group (clinic −0.2 mmHg [−2.2, 1.8]; ambulatory 1.1 mmHg [−0.3, 2.5]). Results for diastolic blood pressure (dBP) were similar. The main limitation of this work was that significant heterogeneity remained. This was at least in part due to different inclusion criteria, self-monitoring regimes, and target BPs in included studies.

**Conclusions:**

Self-monitoring alone is not associated with lower BP or better control, but in conjunction with co-interventions (including systematic medication titration by doctors, pharmacists, or patients; education; or lifestyle counselling) leads to clinically significant BP reduction which persists for at least 12 months. The implementation of self-monitoring in hypertension should be accompanied by such co-interventions.

## Abstract 2

In an individual patient data meta-analysis of randomized trials, Katherine Tucker and colleagues examine the evidence for the effectiveness of self-monitoring of blood pressure in hypertension.

## Author summary

**Background:**

- Self-monitoring of BP appears to lower BP in people with hypertension, over and above usual care.
- Implementation of self-monitoring has been inconsistent, perhaps because important evidence gaps remain regarding how best to use it and for which patient groups.

**Why was this study done?:**

- To better understand the effect of self-monitoring on BP lowering and BP control.
- Specifically, to examine the effect of self-monitoring in combination with various co-interventions, and in different groups of patients.

**What did the researchers do and find?:**

- We undertook a systematic literature search to identify all studies that included self-monitoring of BP in people with high BP.
- For studies published since the year 2000 with at least 6 months of follow-up data and at least 100 patients, we contacted authors to gain access to the original data collected for each individual patient (15/19 studies with the primary outcome provided data: 7,138/8,292 randomised participants).
- We then used these data to perform IPD meta-analysis to evaluate the effect of self-monitoring on BP levels and in the control of hypertension using 1 year of follow-up as our primary end point.
- We predefined levels of intensity of co-intervention and subgroups of patients for further analysis.
- Self-monitoring worked best when combined with more intensive interventions such as self-management, systematic medication titration, or lifestyle counselling, but had little or no effect on its own.
- Self-monitoring was most effective in those with fewer antihypertensive medications and higher baseline sBP up to 170 mmHg. No differences in efficacy were seen by sex or by most comorbidities.

**What do these findings mean?:**

- Self-monitoring can be recommended to lower BP when combined with co-interventions involving individually tailored support.
- Self-monitoring alone does not seem to lower BP but may be useful for other reasons including engaging with patients or reducing clinician workload.

## Introduction

Treatment of hypertension results in significant reductions in risk of subsequent cardiovascular disease [1,2]. Despite strong evidence for such treatment, international epidemiological studies suggest that many people remain suboptimally controlled [3]. Self-monitoring of blood pressure (BP), where individuals measure their own blood pressure, usually in a home environment, can improve BP control and is an increasingly common part of hypertension management. Such monitoring can be accompanied by additional support such as from a nurse or pharmacist [4].

Self-monitoring is well tolerated by patients and has been shown to be a better predictor of end organ damage than clinic measurement [5–8]. This is despite potential issues with quality control of self-measurement such as poor technique or withholding of results [9,10]. The latter can be reduced to an extent by the use of telemonitoring [11].

Previous meta-analyses have shown that self-monitoring reduces clinic BP by a small but significant amount compared to conventional care (around 4/1.5 mmHg) [4,12–14]. Analysis by Bray and colleagues suggested that when self-monitoring was accompanied by a co-intervention, participants were more likely to meet target BP, but it remains unclear which interventions are most effective and what specific populations (if any) should be targeted [14].

The aim of this work was therefore to use individual patient data (IPD) from relevant trials to assess the effectiveness of BP self-monitoring on BP reduction and hypertension control, evaluating how best to utilise self-monitoring of BP and to determine which subpopulation is most likely to benefit.

## Materials and methods

This study systematically reviewed the existing literature to identify randomised trials examining the efficacy of self-monitoring of BP compared to control. Authors of all eligible trials were approached for access to IPD. A protocol with detailed methods has been published previously [15]. The methods used are summarised below.

### Data sources and searches

Medline, Embase, and the Cochrane Library were searched for trials using BP self-monitoring in hypertensive patients (S2 Fig; search date June 2016).

### Study selection

Two reviewers (RM and KT) independently assessed the articles for eligibility and inclusion; disagreements were resolved by discussion. Randomised trials were eligible that recruited patients with hypertension being managed as outpatients using an intervention that included self-measurement of BP. Self-monitoring had to be without medical professional input (i.e., by patient with or without carer support) and using a validated monitor, with or without other co-interventions, and where a comparator group had no organised self-measurement of BP. Included studies were required to have involved at least 100 patients, followed up for at least 24 weeks, and to have been published since 2000. This was to ensure that self-monitoring equipment was likely to be relevant to contemporary medical management (i.e., automated oscillometric monitors). Relevant outcomes were systolic blood pressure (sBP) and/or diastolic blood pressure (dBP) measured in clinic, by researcher or by ambulatory measurement, and achievement of BP control.

### Data extraction and quality assessment

Authors whose trials met the inclusion criteria were approached for provision of IPD including demographic details, comorbidities, antihypertensive medications, lifestyle factors, and BP end points (clinic and/or ambulatory). Study-level data were extracted where available from published articles and checked by the original authors. In particular, any co-interventions were carefully documented and prospectively allocated to 1 of 4 levels of interventional support based on a previous classification [4] (S1 Table).

Study quality was assessed in terms of potential bias from randomisation, blinding, outcome assessment, and method of analysis using an adaptation of the Cochrane tool [16].

Original data were kept on a secure server and assembled in a consistent format for all trials. Three researchers (KT, RM, and JS) cross-checked trial details, summary measures, major outcomes, and definitions against published articles. Any apparent inconsistencies were checked with the original trial authors. Overall ethical approval was not required as this study does not include identifiable data; collaborating groups gained individual approval where required for data sharing.

### Data synthesis and analysis

A 2-stage IPD meta-analysis was conducted using linear regression for continuous outcomes and logistic regression for proportions, aggregated across studies by random-effects inverse variance methods. Intention-to-treat comparisons of outcomes between the self-monitoring and comparator arms were summarised with forest plots using the I-squared (*I*^2^) statistic for heterogeneity. Regression models were adjusted for age, sex, baseline clinic BP, and diabetic status (the latter due to the lower BP target generally used in a diabetic population).

The primary outcomes were change in sBP and dBP at 12 months and likelihood of uncontrolled BP below target at 12 months (control as defined by each trial). Analyses are reported in subgroups, by pre-specified level of self-monitoring intervention as described in Table 1 and in the published protocol [15].

**Table 1:** Characteristics of the included studies.

Lead author(s) [Ref] Country, Year	Study	Setting	Self-monitoring	Number of readings / session	Co-interventions	Pre-defined level	Comparison	Home target (mmHg)	Clinic target (mmHg)	Baseline BP ±SD mmHg	No. randomised	Data available	No. 6M	No. 12M	No. 18M
**Halme/ Kantola** [41] **Finland, 2005**	HOMER	Primary care	Daily for 1 week every 2 months	2	None	**1**	Usual care	135/80	140/85	157/93±18/8	269	231	170	N/A	N/A
**McManus** [24] **UK 2005**	TASMINH	Primary care	Monthly in GP practice waiting room	2	None	**1**	Usual care	140/85 140/80 DM	140/85 140/80 DM	156/88±15/7	441	440	413	401	N/A
**Bosworth** [26] **US, 2007**	HINTS	Primary care	3 days per week	1	Behavioural intervention	**3**	Usual care	135/85 135/80 DM	140/90 135/80 DM	129/77±18/13	593	591	535	523	N/A
					Or meds management	**4**				130/78±19/14					
					Or both	**4**				128/77±19/13					
**Verberk** [25] **Netherlands, 2007**	HOMERUS	Primary care and outpatients	1 week per month, then 1 week every 2 months	3	None	**1**	Usual care with monthly clinic visits then 2 monthly	140/90	140/90	164/96±17/10	517	517	N/A	434	N/A
**Green** [42] **US, 2008**	eBP	Primary care and outpatients	2 days per week	2	Website and email	**2**	Usual care	135/85	140/90	151/89±12/9	778	778	N/A	730	N/A
					+/- web-based pharmacist management	**4**				151/89±12/9					
**Bosworth** [27] **US, 2009**	TCYB	Primary care	3 days per week	1	None	**1**	Usual care	135/85	140/90	125/71±16/10	636 (476 applicable)	446	383	350	324
					Or behavioural interventions, education, and support	**4**		130/80 DM	130/80 DM	125/71±18/11					
**Parati & Omboni** [33] **Italy, 2009**	TeleBPcare	Primary Care	3 days per week	2	Telemonitoring	**2**	Usual care	135/85	140/90	146/88±12/8	329	298	298	N/A	N/A
**Godwin** [43] **Canada, 2010**		Primary Care	At least weekly	1	None	**1**	Usual care	135/85	140/90	144/81±18/11	552	458	458	458	N/A
**Earle** [23] **UK, 2010**		Community (recruited through a hospital site)	Weekly	1	Blood glucose testing, text/app system with feedback from clinicians	**4**	Usual care	140/90	140/90	131/77±17/10	137	126	126	N/A	N/A
**McManus** [36] **UK, 2010**	TASMINH2	Primary care	Daily for the first week of each month	2	Telemonitoring and self- titration	**3**	Usual care	130/85 130/75 DM	140/90 140/80 DM	152/85±12/9	527	527	480	480	N/A
**Hebert** [40] **US, 2011**		Outpatients and primary care	Variable	Not clear	None	**1**	Usual care		140/90	153/86 16/13	416	416	N/A	N/A	286
					Or nurse support	**4**			130/80DM	153/86 18/13					
**Wakefield** [31] **US, 2011**		Primary care	Daily	1	Low-intensity management algorithm	**2**	Usual care	135/85	135/85	135/72±18/11	302	300	268	261*	N/A
					High-intensity algorithm	**3**		130/80 DM	130/80 DM	136/74±19/11					
**Bove** [44] **US, 2013**	HTN	Primary care and outpatients	2 days per week	Not clear	Telemonitoring	**2**	Usual care		140/90	155/88±15/11	241	235	202	N/A	N/A
**Kerry** [39] **UK, 2013**		Community (recruited from stroke services)	Daily in week 1, then 1 day per week	3	Nurse-led telephone support	**2**	Usual care	130/80	140/85	138/74±21/12	381	381	352	334	N/A
**Magid** [50] **US, 2013**		Primary care	3 days per week	1	Patient education and BP reporting; or patient education, BP reporting, and pharmacist management	**4**	Usual care	135/85 125/75 DM/CKD	140/90 130/80 DM/ CKD	147/15±89/10	338	326	326	N/A	N/A
**Margolis** [37] **US, 2013**	Hyperlink	Primary care	3 days per week	2	Telemonitoring and pharmacist management	**4**	Usual care	135/85 125/75 DM/CKD	140/90 130/80 DM/CKD	148/85±13/12	450	450	403	388	370
**McKinstry** [45] **UK, 2013**	HITS	Primary care	Daily in week 1, then at least 1 day per week thereafter	2	Optional automated telemonitoring	**2**	Usual care	135/85	140/90	153/91±15/11	401	401	374	N/A	N/A
**Parati** [32] **Italy, 2013**	TeleBPMET	Primary care	3 days per week	2	Telemonitoring	**2**	Usual care	135/85	140/90	147/90±12/8	252	182	181	179	N/A
**Green** [51] **US, 2014**	eCare		At least 1 day per week for 2 months, then 1 day per fortnight for 2 months, then monthly	2	Dietician with BP plan and visits (weekly for 2 months, fortnightly for 2 months, then monthly)	**4**	Usual care	135/85	140/90	150/92±12/9	101	101	90	N/A	N/A
**Leiva** [46] **Spain, 2014**	Adherencia	Primary care	Weekly, with morning and afternoon readings	3	Motivational interview, pillbox reminder, family support, BP and medication reminder form, and pharmacist review	**3**	Usual care	135/85	140/90 and 130/80 for DM or CKD	156/84±15/11	221	215	N/A	215	N/A
**McManus** [35] **UK 2014**	TASMIN-SR	Primary care	Daily for the first week of each month	2	Self-management	**3**	Usual care	120/75	130/80 ST	144/80±13/10	552	450	439	450	N/A
**Ogedegbe** [34] **US, 2014**	CAATCH	Primary care	3 days per week	1	Education, lifestyle, and behavioural support	**3**	Usual care			151/91±17/10	1,039	997	610	691	N/A
**Stewart** [48] **Australia, 2014**	HAPPy	Primary care	Several readings per week	1	Pharmacist management with motivational interviewing, medication review, education, and optional refill reminders	**4**	Usual care	140/90 and 130/80 for DM and CKD	140/90 and 130/80 for DM and CKD	141/84±20/11	395	391	351	N/A	N/A
**Yi** [47] **US, 2015**		Primary care	As prescribed by their doctor	Variable	Educational material on hypertension	**1**	Usual care	140/90 or 130/80 DM or CKD	140/90 or 130/80 DM or CKD	152/83±16/11	900	828	529	N/A	N/A
**Parati** (unpublished study) **Italy**	AUPRES	Primary care	3 days per week	3		**1**	Usual care	135/85	140/90	154/95±15/8	407	407	407	407	407

Summary information of included studies. Differences between number randomised and data available for Godwin (2010) and McManus (2014) are due to the use of complete case data only. The difference observed in Bosworth (2009) results from the removal of the behavioural intervention arm and patients with missing baseline BP data. Abbreviations: BP, blood pressure; CKD, chronic kidney disease; DM, diabetes mellitus; GP, general practitioner; M, months; ST, self-tested BP.

*Participants self-monitored for 6 months; follow-up data collected at 12 months.

Subgroup analyses examined the effect of self-monitoring on BP mean and control by age, sex, baseline sBP, the presence and number of antihypertensive medications prescribed, and comorbidities (myocardial infarction [MI], stroke, diabetes mellitus [DM], chronic kidney disease [CKD], and obesity [defined as a body mass index (BMI) ≥ 30 kg/m^2^]). All subgroup analyses were adjusted for age, sex, baseline clinic BP, level of intervention, and individual study (contributing to each analysis).

Sensitivity analyses included incorporation of aggregate data from studies that did not contribute IPD [17–23], exclusion of individual patients for whom a lower home BP target was not used (due to study design or the presence of comorbidities such as diabetes) [24–27], influence of BP inclusion criteria (clinic or ambulatory) from ambulatory outcome studies, different assumptions regarding BP of patients lost to follow-up (controlled or uncontrolled), and influence of adjusting for medication changes (in those studies which recorded changes in medication). Finally, the influence of each study on the overall results was assessed using an influence analysis. Egger’s test for funnel plot asymmetry was applied to consider possible publication bias (S21 Fig) [28].

There were no deviations from the protocol [15]. Five post-hoc analyses were undertaken: firstly, an additional subgroup analysis was carried out (resistant hypertension [defined as BP > 140/90 mmHg and 3 medications at baseline or any BP level and 4 or more medications at baseline]); secondly, the distribution of baseline antihypertensive medications was compared in patients with and without a history of stroke using Pearson’s chi-squared; thirdly, the effectiveness of self-monitoring in stroke was assessed controlling for the number of baseline medications; fourthly, the influence of blinding was assessed; and finally, sBP was plotted against medication changes.

### Statistical software and presentation

All analyses were conducted using STATA version 13.1 (MP parallel edition, StataCorp, College Station, Texas, USA), using the ipdmetan package [29]. Data are presented as proportions of the total study population, means with standard deviation or relative risk (RR) with 95% confidence intervals unless otherwise stated.

## Results

Of 2,846 unique studies from the combined searches, 132 were assessed in full and 36 studies were deemed potentially eligible (S1 Fig). One study which would otherwise have been eligible was excluded because the comparator group used ambulatory monitoring to guide treatment, a control intervention that had not been anticipated in the protocol but which was not comparable to any other included studies [30]. Of the 36 potentially eligible studies, 19 had published data at 12 months, the primary outcome. Authors from 24 of the potentially eligible studies provided IPD, with 1 group submitting additional data from an unpublished study. These 25 studies were published from 2005–2014, were conducted in North America and Europe (11 United States; 6 United Kingdom; 3 Italy; 1 each from the Netherlands, Australia, Spain, Finland, and Canada), and included a wide range of self-monitoring protocols, co-interventions, and populations (Table 1) [23–27,31–48]. Authors from the remaining 12 studies were either unable to provide IPD (2 studies) or did not respond to the request for data (10 studies). Four studies which followed up patients for 12 months did not provide IPD, so that data for the primary outcome were available from 15/19 studies (7,138/8,292 [86%], of potential participants) (S2 Table) [17,18,22,49]. A total of 838 patients (12%) were lost to follow-up across all included studies, and a further 227 patients from the potentially available studies were lost to follow-up, leaving 6,300/7,227 patients (87%) for inclusion in the final analysis of the primary outcome (12 months follow-up).

Overall, the information from the included trials was judged to be at low risk of bias: most studies used computerised generation of randomisation sequences (23/25, 92%), appropriate allocation concealment (24/25, 96%), and all used an intention-to-treat approach with either multiple imputation for missing data or analysis of complete cases. Most studies (19/25, 76%) followed up more than 80% of participants, but only 12/25 (48%) used blinded assessment of outcome (S3 Table). An influence analysis assessed the impact of each individual study on the overall results. Included studies were predominantly publically funded (S4 Table).

### Clinic BP

**Fig 1:**
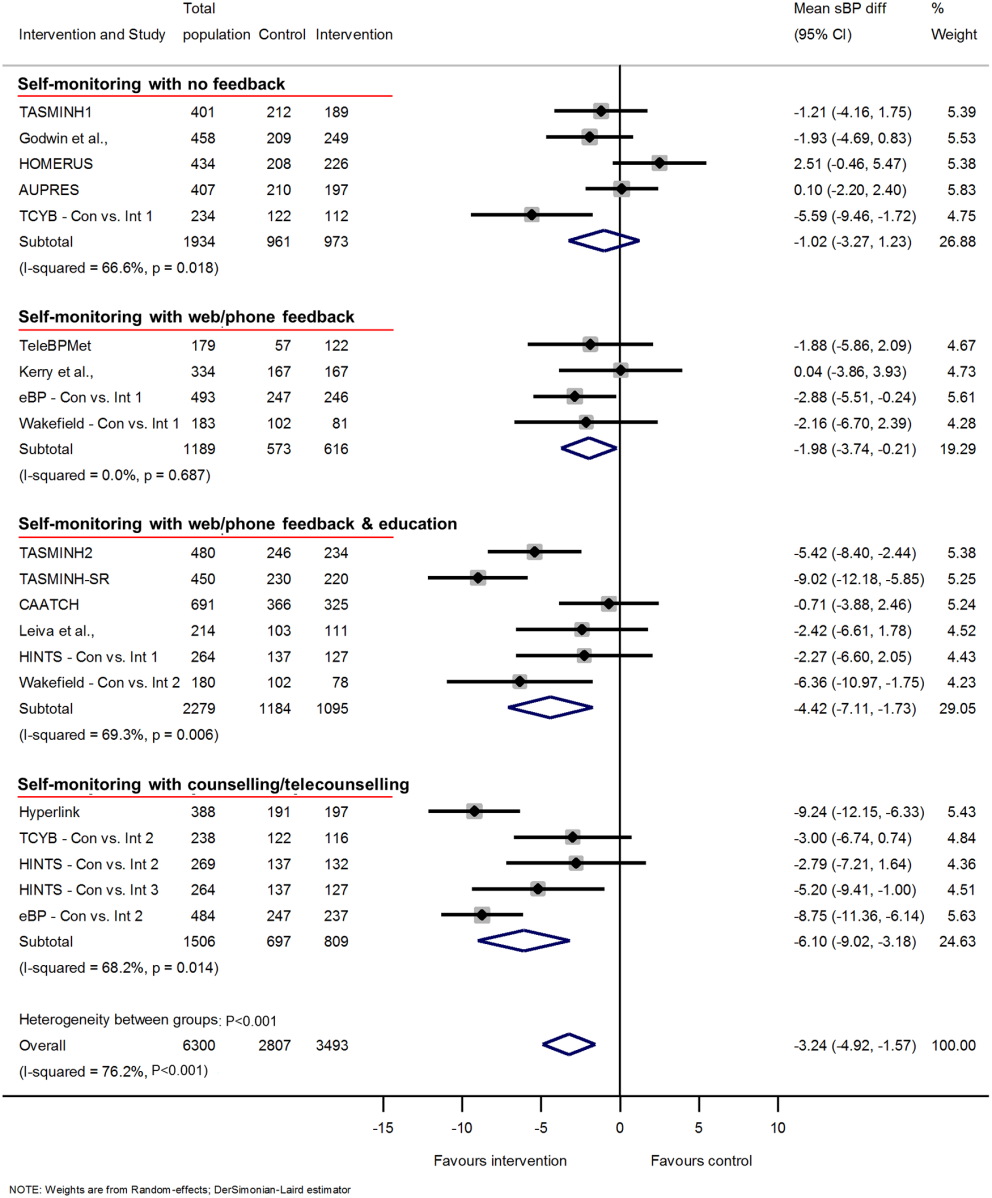
Impact of self-monitoring of BP on clinic sBP according to level of co-intervention support at 12 months (15 studies). Change in sBP adjusted for age, sex, baseline clinic BP, and history of diabetes. The trials are grouped into the 4 levels of intervention, and *I*^2^ and *P* values are shown for each level of intervention and for the overall analysis. Effect of self-monitoring on clinic sBP at 6 and 18 months are shown in S3 and S6 Figs, respectively. Wakefield’s study participants self-monitored for 6 months; follow-up continued to 12 months. Abbreviations: BP, blood pressure; sBP, systolic blood pressure.

**Fig 2:**
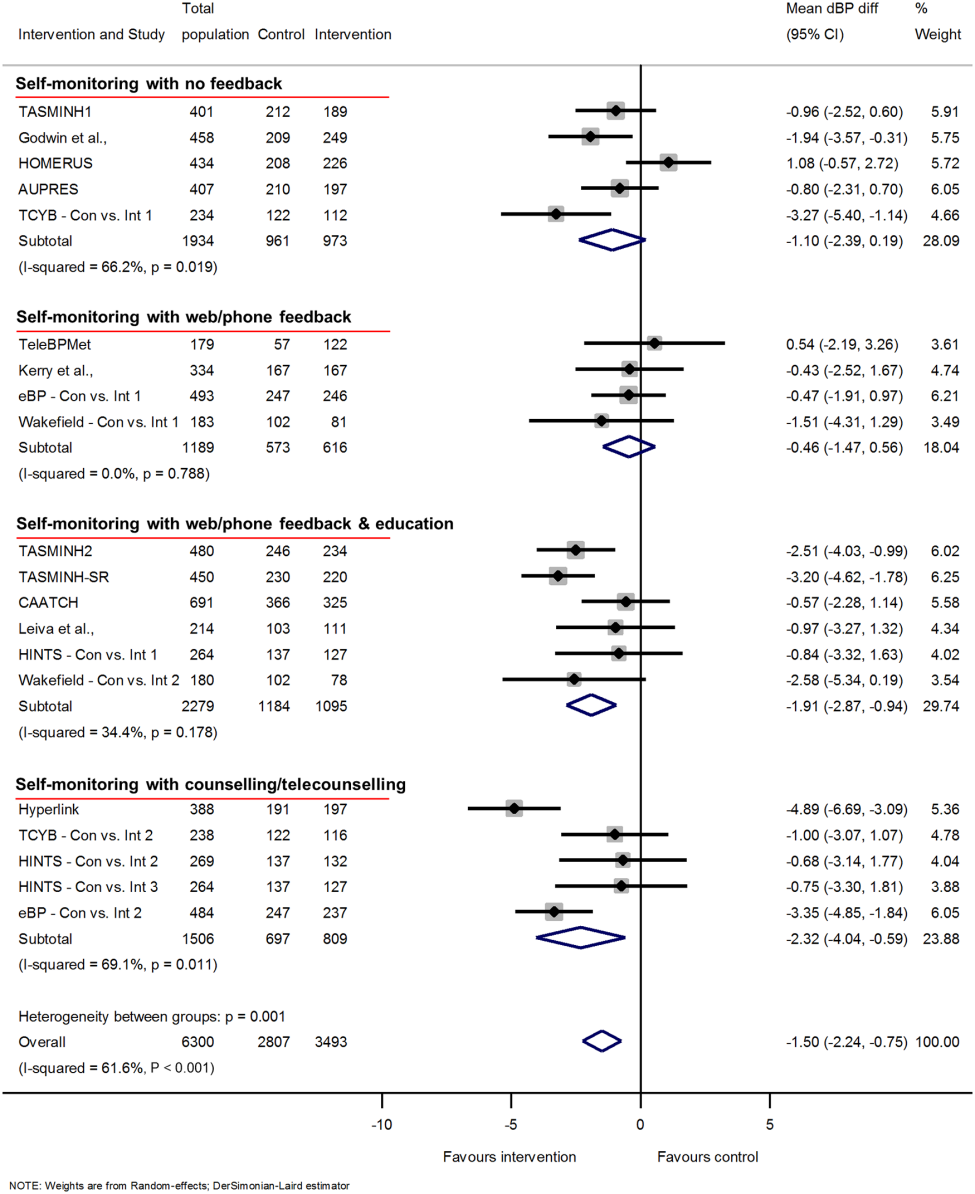
Impact of self-monitoring of BP on clinic dBP according to level of co-intervention support at 12 months (15 studies). Change in dBP adjusted for age, sex, baseline clinic BP, and history of diabetes. The trials are grouped into the 4 levels of intervention, and *I*^2^ and *P* values are shown for each level of intervention and for the overall analysis. Effect of self-monitoring on clinic dBP at 6 and 18 months are shown in S4 and S7 Figs, respectively. Wakefield’s participants self-monitored for 6 months; follow-up continued to 12 months. Abbreviations: BP, blood pressure; dBP, diastolic blood pressure.

Overall, self-monitoring was associated with reduced clinic sBP between baseline and 12-months follow-up compared to usual care (systolic −3.2 mmHg, 95% CI −4.9 to −1.6 mmHg) (Fig 1). Significant heterogeneity was present between studies: *I*^2^ = 76%, *P* < 0.001. Self-monitoring was also associated with reduced dBP at 12-months follow-up (diastolic −1.5 mmHg, 95% CI −2.2 to −0.8 mmHg) and significant heterogeneity remained (*I*^2^ = 62%, *P* < 0.001) (Fig 2). Similar reductions in BP were seen after 6-months follow-up, but the point estimates after 18-months follow-up were smaller, albeit from only 5 studies (S3, S4, S6 and S7 Figs).

### Clinic BP control

**Fig 3:**
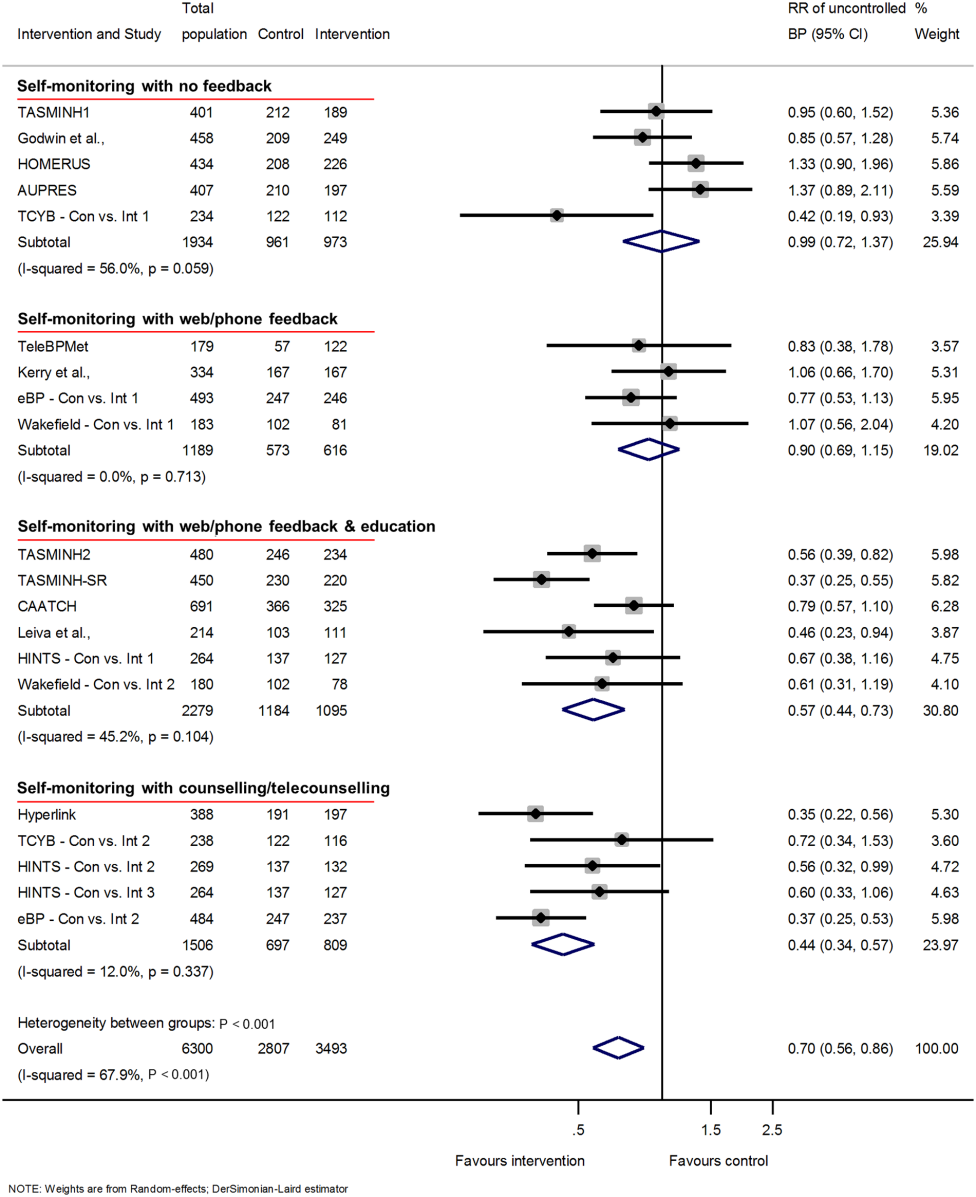
Impact of self-monitoring of BP on the RR of uncontrolled BP at 12 months according to level of co-intervention support (15 studies). RR of uncontrolled BP adjusted for age, sex, baseline clinic BP, and history of diabetes. The trials are grouped into the 4 levels of intervention, and *I*^2^ and *P* values are shown for each level of intervention and for the overall analysis. The effect of self-monitoring on the RR of BP at 6 and 18 months are displayed in S5 and S8 Figs, respectively. Wakefield study participants self-monitored for 6 months; follow-up continued to 12 months. Abbreviations: BP, blood pressure; RR, relative risk.

Clinic BP control was improved at 12-months follow-up (RR of uncontrolled BP 0.7 [95% CI 0.56 to 0.86]) again with significant heterogeneity between groups (Fig 3). Similar results were seen at 6 and 18 months (S5 and S8 Figs, respectively).

### Intensity of co-intervention

The reductions in clinic sBP varied with different levels of intervention: level 1 (with no co-intervention) −1.0 mmHg, [95% CI −3.3 to 1.2 mmHg]; level 4 (personal support throughout the trial) −6.1 mmHg, [95% CI −9.0 to −3.2 mmHg] (Fig 1) (heterogeneity in outcome between different levels of intervention *P* < 0.001). Within predefined categories of intensity of co-intervention, significant heterogeneity remained, apart from within level 2.

A similar pattern of reductions was seen in dBP: level 1 (with no co-intervention) −1.1 mmHg, [95% CI −2.4 to 0.2 mmHg]; level 4 (personal support throughout the trial) −2.3 mmHg, [95% CI −4.0 to −0.6 mmHg] (Fig 2) (heterogeneity in outcome between different levels of intervention *P* < 0.001). Within predefined categories of intensity of co-intervention, significant heterogeneity remained in levels 1 and 4.

BP control (defined according to individual study targets, Table 1) at 12 months also differed by level of intensity. The RR of having uncontrolled BP with a self-monitoring intervention at 12 months varied from level 1 (RR 1.0, 95% CI 0.7 to 1.4) to level 4 (RR 0.4, 95% CI 0.3 to 0.6) (Fig 3) (heterogeneity between levels of intervention *P* < 0.001). Heterogeneity within levels of intervention in this analysis was low for levels 2 and 4 of co-intervention, although the *I*^*2*^ remained above 50% for level 1. Similar results were seen at 6-months follow-up (21 studies) and at 18-months follow-up (5 studies) (S5 and S8 Figs, respectively).

### Ambulatory BP

**Fig 4:**
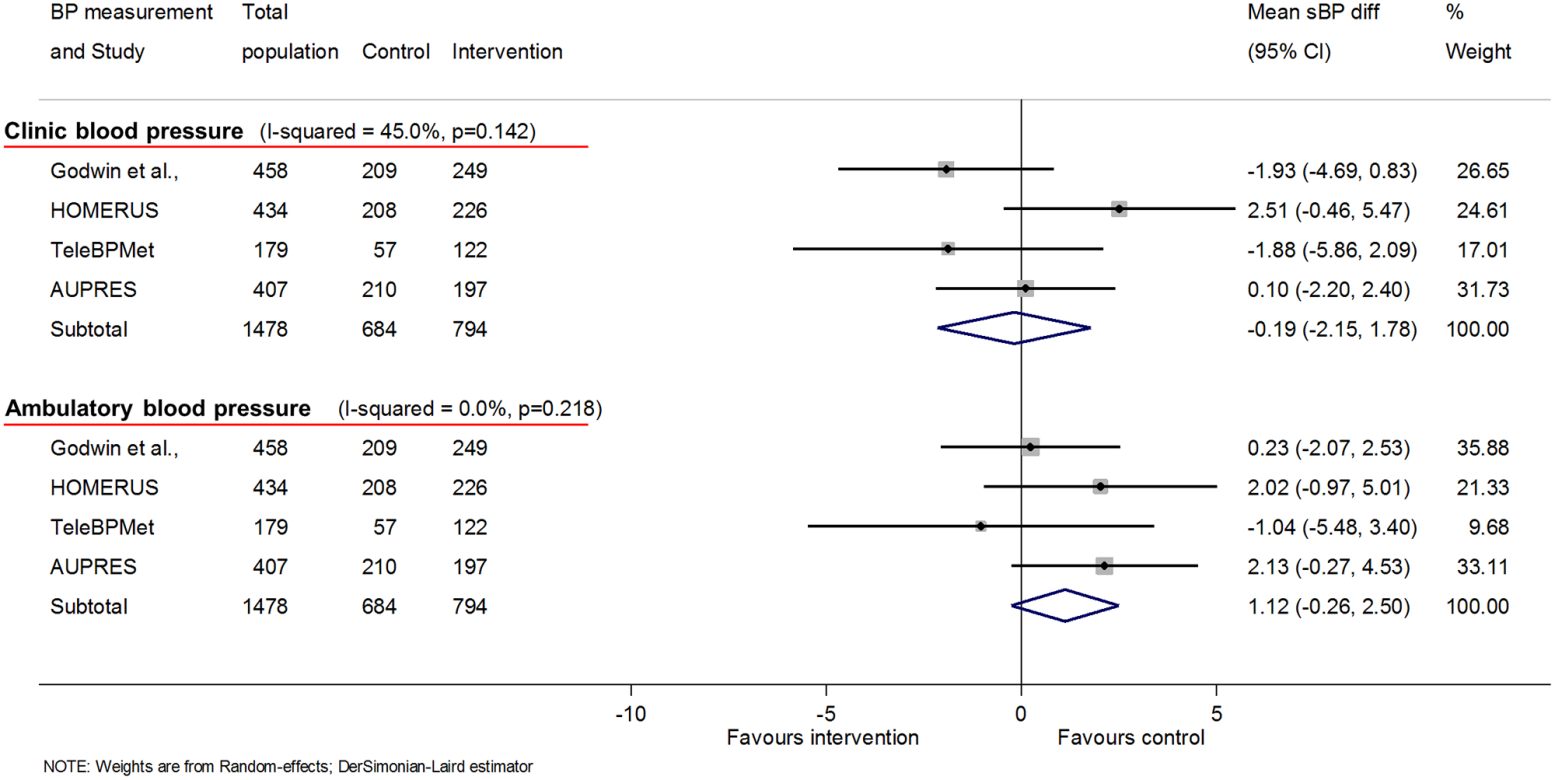
Impact of self-monitoring of BP on clinic and ambulatory sBP at 12 months (4 studies). These 4 studies used both clinic and ambulatory BP as endpoints and so are presented in addition to the overall results in Fig 1, which are for clinic BP alone (including these studies). Change in sBP adjusted for age, sex, baseline clinic BP, history of diabetes, and level of intervention. Effect of self-monitoring on systolic clinic and ambulatory BP at 6 months is in S9 Fig. Abbreviations: BP, blood pressure; sBP, systolic blood pressure.

**Fig 5:**
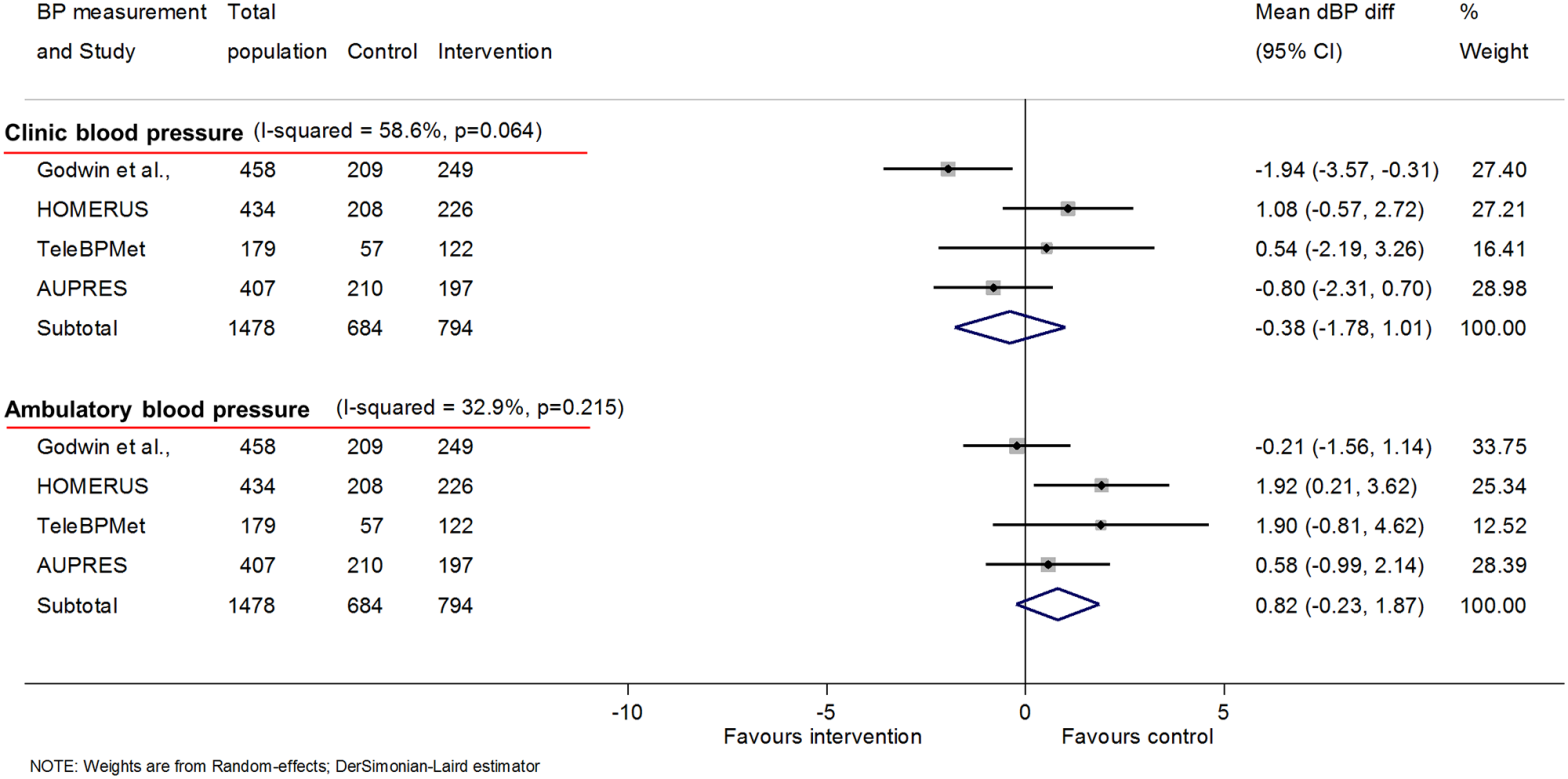
Impact of self-monitoring of BP on clinic and ambulatory dBP at 12 months (4 studies). These 4 studies used both clinic and ambulatory BP as endpoints and so are presented in addition to the overall results in Fig 1, which are for clinic BP alone (including these studies). Change in dBP adjusted for age, sex, baseline clinic BP, history of diabetes, and level of intervention. Effect of self-monitoring on diastolic clinic and ambulatory BP at 6 months is in S10 Fig. Abbreviations: BP, blood pressure; dBP, diastolic blood pressure.

Four studies had data at 12 months using ambulatory BP as the outcome (1,478 participants); these were studies with no co-intervention (level 1; *n* = 3) or automated feedback only (level 2; *n* = 1). No change was seen in ambulatory sBP associated with self-monitoring (1.1 mmHg [−0.3, 2.5]) (Fig 4) or ambulatory dBP (0.8 mmHg [−0.2, 1.9]), and there was no significant heterogeneity between studies in either case (Fig 5). At 6 months, data were available for 5 studies with no difference seen in ambulatory sBP (−1.0 mmHg [−2.8, 0.9]) or dBP (−0.4 mmHg [−1.6, 0.8]) (S9 and S10 Figs, respectively). The additional study, which used a level 3–intensity intervention, increased heterogeneity as it had a significant outcome.

No ambulatory data were available at 18 months.

### Subgroup analysis

**Fig 6:**
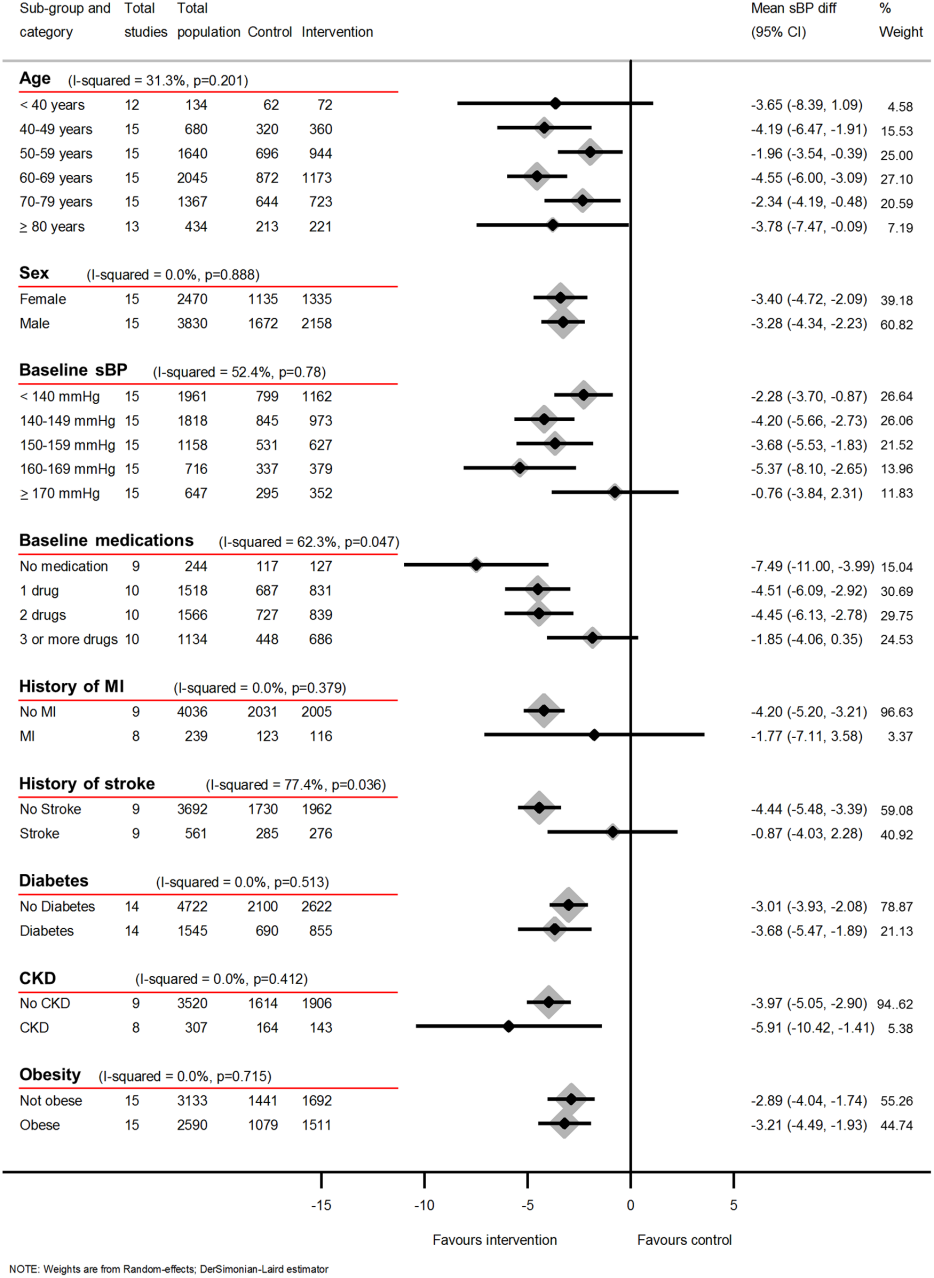
Impact of self-monitoring of BP on clinic sBP at 12 months according to prespecified subgroups (15 studies). Obesity defined as BMI ≥ 30 kg/m^2^. Change in sBP at 12 months adjusted for age, sex, baseline clinic BP, level of intervention, and studies contributing patient data. Abbreviations: BMI, body mass index; BP, blood pressure; CKD, chronic kidney disease; MI, myocardial infarction; sBP, systolic blood pressure.

**Fig 7:**
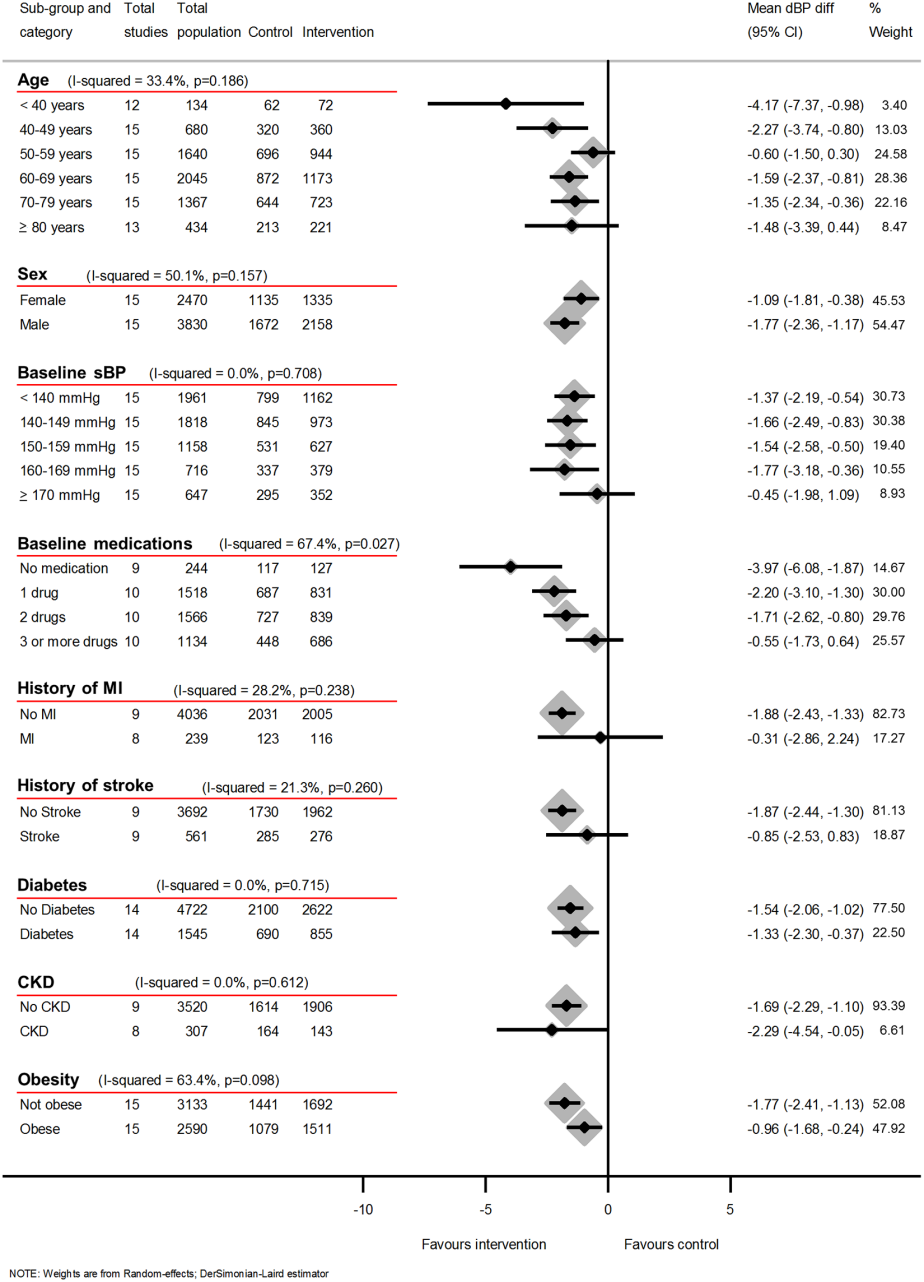
Impact of self-monitoring of BP on clinic dBP at 12 months according to prespecified subgroups (15 studies). Obesity defined as BMI ≥ 30 kg/m^2^. Change in dBP at 12 months adjusted for age, sex, baseline clinic BP, level of intervention, and studies contributing patient data. Abbreviations: BMI, body mass index; BP, blood pressure; CKD, chronic kidney disease; dBP, diastolic blood pressure; MI, myocardial infarction.

**Fig 8:**
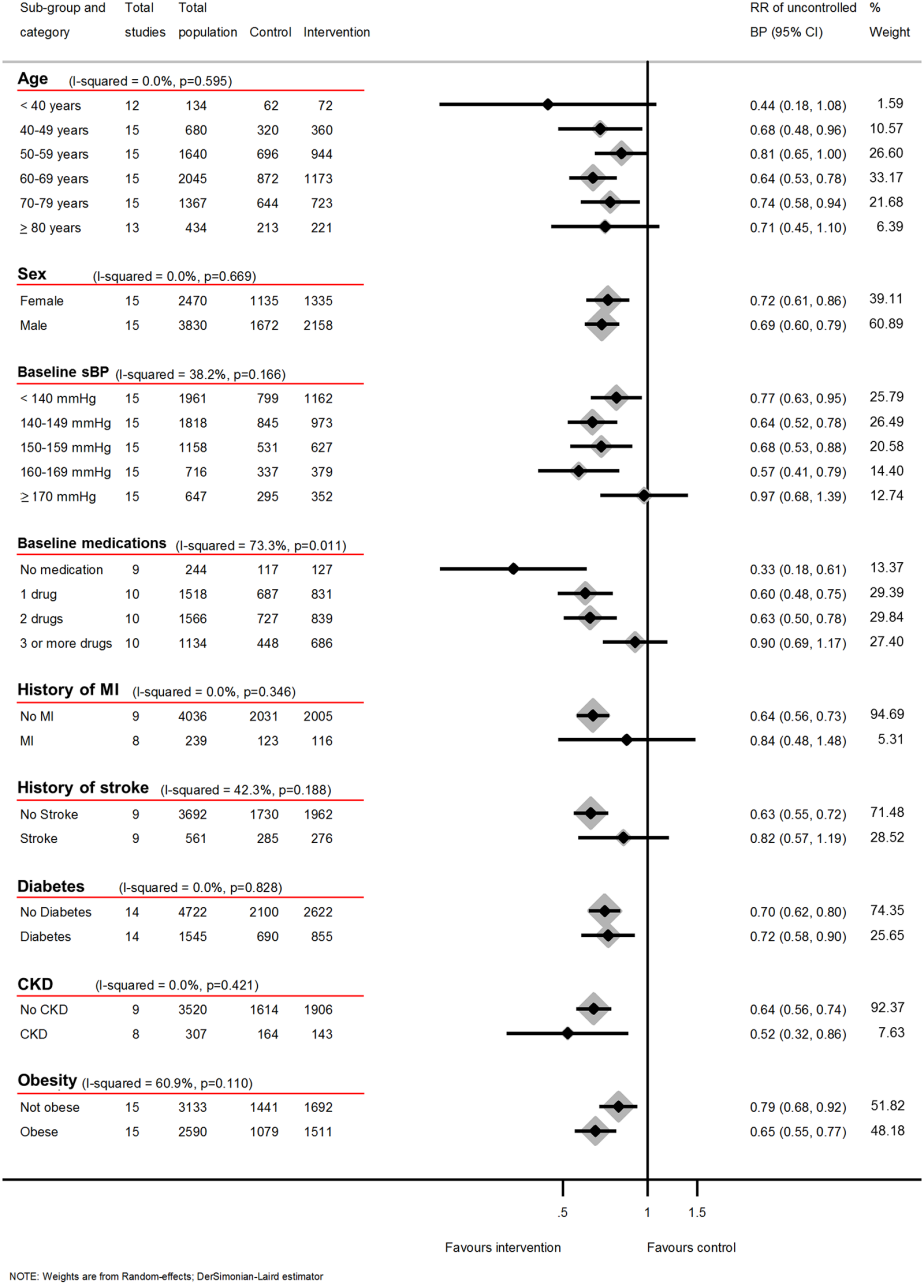
Impact of self-monitoring of BP on the RR of uncontrolled BP at 12 months according to prespecified subgroups (15 studies). Obesity defined as BMI ≥ 30 kg/m^2^. RR of uncontrolled BP at 12 months adjusted for age, sex, baseline clinic BP, level of intervention, and studies contributing patient data. Abbreviations: BMI, body mass index; BP, blood pressure; CKD, chronic kidney disease; MI, myocardial infarction; RR, risk ratio; sBP, systolic blood pressure.

Subgroup analyses using data from 12-months follow-up showed little difference in either reduction of systolic or diastolic clinic BP or likelihood of uncontrolled BP depending on history of MI or presence of CKD or diabetes (Figs 6, 7 and 8) (*I*^2^ ≤ 20% for all subgroups).

However, a history of stroke was associated with a reduced effectiveness of self-monitoring in terms of clinic sBP lowering (*I*^2^ = 77%, *P* = 0.04), though this difference was not observed for dBP or maintained in the likelihood of control analysis (RR *I*^2^ = 42%, *P* = 0.19). Post-hoc analyses showed that the distribution of number of medications between stroke and non-stroke patients was similar (S5 Table), and adjusting for baseline medication use did not explain the lack of effectiveness in patients with stroke. There was moderate heterogeneity between age groups for the effect of self-monitoring on systolic and diastolic clinic BP (*I*^2^ = 31%, *P* = 0.20 and *I*^2^ = 33, *P* = 0.19, respectively) but not in the likelihood of uncontrolled BP (*I*^2^ = 0.0%, *P* = 0.60). Considering the effect of obesity, there was no difference in the effect on systolic clinic BP reduction (*I*^2^ = 0, *P* = 0.72) but there was some evidence of heterogeneity of effect for dBP (*I*^2^ = 63, *P* = 0.10) and the risk of uncontrolled BP (*I*^2^ = 61%, *P* = 0.11).

Fewer baseline antihypertensive medications were associated with larger reductions of BP and better control (Figs 6–8). Post-hoc analyses, comparing those with resistant hypertension to those without, suggested that self-monitoring was less effective at achieving BP control in the former (RR of uncontrolled BP = 0.62, 95% CI 0.54–0.71 [non-resistant hypertension] versus RR of uncontrolled BP = 0.94, 95% CI 0.65–1.36 [resistant hypertension], *I*^2^ = 76%, *P* = 0.04). Similarly, the post-hoc analysis plotting change in sBP against medication changes was consistent with the hypothesis that self-monitoring interventions resulted in BP decreases via increases in prescribed medication (S22 Fig).

### Sensitivity analysis

Inclusion of aggregate data for clinic BP at 12 months from the 4 eligible studies that did not contribute IPD (S2 Table) and exclusion of studies that did not use a lower home BP threshold did not materially change the results (S11 and S12 Figs). The exclusion of studies that randomised on the basis of ambulatory BP monitoring (ABPM) or studies that randomised on clinic BP did not change the impact of clinic or ambulatory measurement of sBP at 12 months (S13 and S14 Figs). Assuming patients lost to follow-up had uncontrolled BP attenuated the results, whereas assuming that they had controlled BP accentuated them (S15 and S16 Figs, respectively). Exclusion of patients with controlled BP at baseline also accentuated the results (S17 Fig). A post-hoc comparison of studies with blinded outcome (2,829 patients) versus unblinded (3,257 patients) showed that blinding was associated with a reduced point estimate for the change in sBP at 12 months in those studies examining higher-level interventions, albeit with overlapping confidence intervals (level 1 & 2 intervention studies: −1.51, 95% CI −4.06 to 1.04 [blinded] versus −0.83, 95% CI −2.38 to 0.73 [unblinded]; level 3 & 4 intervention studies: −4.67, 95% CI −7.51 to −1.84 [blinded] versus −6.16, 95% CI −9.36 to −2.95 [unblinded]).

Where studies had measured changes in antihypertensive medication over time, there was evidence of attenuation of the change in sBP when the analysis was adjusted for change in medication (S18 and S19 Figs). The influence analysis did not suggest that any one study was materially influencing the results (S20 Fig and Egger’s test found no evidence of asymmetry in the funnel plot (*P* = 0.9, S21 Fig).

## Discussion

### Main findings

Using IPD from 25 studies totalling 10,487 patients, this meta-analysis provides strong evidence that the degree of BP lowering is related to the intensity of the co-intervention (i.e., additional support) combined with self-monitoring, with little or no effect from self-monitoring alone.

These results held whether systolic or diastolic clinic BP or clinic BP control were assessed and were consistent at both 6 and 12 months. No data were available from studies with intensive co-interventions which used ambulatory BP monitoring to measure outcomes at 12 months or longer, and those with little or no co-intervention showed similar effects to the clinic BP data (no effect in either case). There was a suspicion of attenuation of the effect of self-monitoring in the few studies to date that have followed up patients for longer than 1 year but data were sparse. Future research might be directed towards longer studies with ambulatory BP measurement (or other measurements to reduce the white coat effect) for outcomes. Self-monitoring appeared most effective at lowering BP in people on fewer BP medications at baseline, and there was a suggestion of a greater effect with higher BP—provided BP was not 170 mmHg or above. Analyses considering those with apparent resistant hypertension at baseline suggested that self-monitoring works less well in this group, but this analysis was not prespecified, could not take into account dose of antihypertensive medication, and should be interpreted with caution. In terms of comorbidities, the effects of self-monitoring were similar whether or not an individual had a history of MI, diabetes, or CKD. In people with previous stroke, there may be a reduced effect of self-monitoring but this did not reflect more intensive treatment prior to randomisation.

### Strengths and weaknesses

To our knowledge, this is the first analysis of self-monitoring of BP to use IPD from a wide range of self-monitoring trials from North America, Australia, and Europe and including both specialist and primary care settings. IPD allowed for standardised adjustment of outcomes and sufficient power to detect differences between subgroups.

An important issue in IPD analysis is selection of studies. The BP—SMART collaboration has gained access to a large number of datasets; nevertheless, some studies were not available due to unavailability of data or lack of response. Despite this, only 4 studies eligible for the primary outcome (14% of available patient data) were unable to provide data, and sensitivity analyses suggested no material change in the results when the published aggregate data from these studies were included.

Quality of included studies was adequate in terms of randomisation sequences, appropriate allocation concealment, and analyses. Follow-up was high for most studies but only half used blinded assessment of outcome. However, a post-hoc sensitivity analysis showed no difference in results from blinding, perhaps because in most studies BP was assessed using automated monitors reducing the chance of bias.

Despite the use of IPD and the division of studies into subgroups, significant heterogeneity remained, which limited the ability to do meta-analysis. However, this does not negate the conclusion that the evidence for both BP reduction and control is stronger for higher-intensity interventions and weak for self-monitoring alone. The hypothesis that effect would vary with level of intervention was prespecified and the categorisation of studies into 4 levels of intervention was agreed to by all study investigators before results were available.

Whilst all included studies compared self-monitoring of BP to control groups without self-monitoring, inevitably different investigators used different protocols and therefore studies differed in inclusion criteria, self-monitoring regime, and target BPs. These issues could at least in part explain the remaining heterogeneity between studies. The exclusion of studies which did not use lower BP targets for self-monitored BP did not change the results, but even IPD analysis is unable to take differences between studies entirely into account and this may be reflected in the heterogeneity which remained. Significance tests should be interpreted with caution when, as in Figs 6 and 7, multiple coequal exposures are under test; however, the 3 *P* values ≤ 5% for heterogeneity across these 18 tests are unlikely to be all due to chance alone and the tests were prespecified.

Most outcome data were based on clinic measurement of BP, which is what was used by the majority of trials of outcome of hypertension treatment [1]. Ambulatory monitoring might reduce any attenuation to the white coat effect from repeated habituation to measurement but, whilst 6 studies used ambulatory BP as an outcome [25,32,33,43,45] (including 1 unpublished study), all but 1 of these used less intensive or no co-interventions. The single intensive study with an ambulatory outcome had data to 6 months and a positive result, whereas the remaining 4 studies showed no impact on ambulatory BP in common with the pooled results for clinic BP. Other studies have used multiple automated BP measurements in the clinic to assess habituation and have found no evidence that the BP differences are removed when the white coat effect is reduced, though further studies examining the effects of self-monitoring with intensive co-interventions on outcomes which reduce white coat effects are arguably needed [36] [35].

Even with IPD, issues such as loss to follow-up may be important. Included studies had rates of follow-up between 58% and 98% at 12 months with most studies following-up around 90%. In the main analysis, formal methods for handling missing data were not used since methods for imputation in IPD meta-analysis are in their infancy; however, the impact of each individual study on the overall results as assessed by the influence analysis suggests that factors such as differential follow-up between studies were unlikely to have affected the results [52].

The outcomes included in this review are all related to BP. Whilst BP is directly related to stroke and coronary heart disease risk, it is nevertheless an intermediate outcome. Ideally, such hard outcomes would be directly measured in trials. However, because of relatively short follow-up and small numbers of participants, few included individual trials did so.

### Comparison with the previous literature

There have been previous systematic reviews of trials of self-monitoring [4,13,14,53], including those focussing on specific outcomes such as adherence [54] or processes such as telemonitoring [55], but all previous analyses have relied on summary statistics rather than IPD. Compared to the most recent and comprehensive summary data review, the current study has provided pooled estimates of the effect of self-monitoring with different levels of co-intervention, suggests that self-monitoring alone has little impact on BP, and provides new evidence that the level of BP reduction is related to the intensity of the co-intervention [4].

Self-monitoring in the absence of such a co-intervention had little effect on BP. This is not to say that self-monitoring alone should be discouraged, for it brings other advantages both theoretical (better estimation of the underlying BP, increased self-efficacy for the patient) [6] and practical (increased adherence, reduced need for monitoring within the clinic, and identification of white coat and masked hypertension) [24,54]. These advantages are despite any potential inaccuracy caused by individuals not conforming to the recommended self-monitoring regime [9,10].

Obese patients had similar BP reductions to non-obese individuals but greater chance of BP control, which does not reflect differences in mean BP. The findings concerning patients with previous stroke and resistant hypertension require some caution, particularly the latter which was a post-hoc analysis, but have not been previously described. In the case of stroke, the results do not appear to be due to baseline intensity of antihypertensive treatment and warrant further study as more data become available.

### Meaning of the study

Combining self-monitoring with increased collaboration between patient and either a nurse, physician, or pharmacist can result in important decreases in BP (6 mmHg systolic on average for the more intensive co-interventions) and improved control. The mechanisms for these reductions in BP could include lifestyle changes (no data available); increased adherence to medication (no data available) [54]; or increased prescription of medications, i.e., overcoming clinical inertia (data available from 11 studies). In order to assess the impact of enhanced medication prescription, number of medication changes was plotted against changes in BP and showed that increased numbers of medication changes were weakly correlated with reduced BP (S22 Fig). Whatever the mechanism, the literature suggests that a 6 mmHg reduction in sBP, as observed in higher-intensity interventions, would reduce subsequent stroke by more than 20% [56]. Considering the content of such interventions is an important part of decision-making in the implementation of self-monitoring. Table 1 and S1 Table describe the key characteristics of effective interventions which depend on actively intervening in terms of medication titration and/or health behaviours. Much of the effect appears to be associated with one-to-one intervention combined with medication intensification. Self-monitoring can therefore facilitate significant improvements in BP level and control but should not necessarily be seen as reducing clinical input because clinical input within the co-interventions is often required for effective BP lowering.

The recent SPRINT trial results suggest that more intensive BP interventions are likely to be important in terms of morbidity and mortality [57]. Increasing the level or intensity of intervention also increases the cost of an intervention, both directly to the health provider and also in terms of patients’ time. Understanding the relative cost-effectiveness of the different co-interventions is likely to be important in deciding policy in this area and will require further work.

The effects appear to be independent of age, sex, and a range of comorbidities (such as MI, CKD, diabetes, and obesity), but there was a suggestion that people receiving less intensive antihypertensive treatment, and those with the highest BPs (up to 170 mmHg systolic), may have the most to gain, presumably because they are not already receiving sufficient doses of medication. Conversely, with resistant hypertension there appeared to be little effect from self-monitoring. Similar results for stroke should be interpreted cautiously and warrant further study.

The data presented appear to indicate a potential attenuation of the beneficial effects of self-monitoring over time (see S6, S7 and S8 Figs). We believe that the key issue is a need for longer studies (at least 2 years, and preferably 5 years or more) that are accompanied by investigation of how best to administer a self-monitoring—based intervention in the long term, including whether it should be perhaps “topped up” with additional training over time.

Finally, we know from the individual trials that only a proportion of those with hypertension will be suitable for self-monitoring. Despite this, the numbers of people with hypertension and access to their own BP monitor are likely to be well into the tens of millions internationally and represent an important population to engage with [58,59].

### Future research

Several unanswered questions remain. Ultimately, trials including cardiovascular endpoints would provide the strongest evidence for self-monitoring in the management of hypertension but may not be appropriate given the strong evidence linking BP to outcome. Further consideration of self-monitoring in the presence of comorbidities seems warranted, particularly for stroke. Furthermore, this review has not included economic outcomes (available from 6 of the included studies) or quality of life measures (available in 8 of the included studies), and these outcomes form part of the next series of investigations for this collaboration.

### Conclusions

Self-monitoring of BP combined with co-interventions involving individually tailored support lowers clinic BP but has little effect on its own. Self-monitoring supported by such co-interventions should be recommended as part of routine clinical practice in international guidelines and further research should determine the most cost-effective means of supporting implementation.

## Supporting information

S1 PRISMA Checklist PRISMA checklist. (DOC)

S1 Text The protocol paper published by the BMJ Open (available at http://bmjopen.bmj.com/content/bmjopen/5/9/e008532.full.pdf). (PDF)

S1 Table Levels of self-monitoring intervention. Levels used to describe the included self-monitoring interventions. * 1:1 contact or support in this context refers to contact over and above that in usual care. Abbreviation: BP, blood pressure.
(DOCX)

S2 Table Studies not included in the IPD analysis. *Data were available from trials including 7,138/8,292 (86%) of patients randomised. ^+^Data at 6 months follow-up were available from 8,563/12,822 (67%) of patients randomised. Abbreviation: IPD, individual patient data.
(DOCX)

S3 Table Assessment of bias. *Due to the nature of the intervention, the participants in all studies were aware that they were in the self-monitoring group.
(DOCX)

S4 Table Funding provided to the included studies. Table showing the funding of the included studies.
(DOCX)

S5 Table Distribution of baseline medications by history of stroke. (DOCX)

S1 Fig Flow diagram of the systematic search and selection of relevant studies. Flow diagram of the systematic review and selection of studies for the IPD.
(DOCX)

S2 Fig Example search strategy. An example search from Medline.
(DOCX)

S3 Fig Impact of self-monitoring of BP on clinic sBP according to level of co-intervention support at 6 months (21 studies). Change in sBP adjusted for age, sex, baseline clinic BP, and history of diabetes. Abbreviations: BP, blood pressure; sBP, systolic blood pressure.
(TIF)

S4 Fig Impact of self-monitoring of BP on clinic dBP according to level of co-intervention support at 6 months (21 studies). Change in dBP adjusted for age, sex, baseline clinic BP, and history of diabetes. Abbreviations: BP, blood pressure; dBP, diastolic blood pressure.
(TIF)

S5 Fig Impact of self-monitoring of BP on the RR of uncontrolled BP at 6 months according to level of co-intervention support (21 studies). RR of uncontrolled BP adjusted for age, sex, baseline clinic BP, and history of diabetes. Abbreviations: BP, blood pressure; RR, relative risk.
(TIF)

S6 Fig Impact of self-monitoring of BP on clinic sBP according to level of co-intervention support at 18 months (5 studies). Change in sBP adjusted for age, sex, baseline clinic BP, and history of diabetes. Abbreviations: BP, blood pressure; sBP, systolic blood pressure.
(TIF)

S7 Fig Impact of self-monitoring of BP on clinic dBP according to level of co-intervention support at 18 months (5 studies). Change in dBP adjusted for age, sex, baseline clinic BP, and history of diabetes. Abbreviations: BP, blood pressure; dBP, diastolic blood pressure.
(TIF)

S8 Fig Impact of self-monitoring of BP on the RR of uncontrolled BP at 18 months according to level of co-intervention support (5 studies). RR of uncontrolled BP adjusted for age, sex, baseline clinic BP, and history of diabetes. Abbreviations: BP, blood pressure; RR, relative risk.
(TIF)

S9 Fig Impact of self-monitoring of BP on clinic and ambulatory sBP at 6 months. Change in sBP adjusted for age, sex, baseline clinic BP, history of diabetes, and level of intervention. Abbreviations: BP, blood pressure; sBP, systolic blood pressure.
(TIF)

S10 Fig Impact of self-monitoring of BP on clinic and ambulatory dBP at 6 months. Change in dBP adjusted for age, sex, baseline clinic BP, history of diabetes, and level of intervention. Abbreviations: BP, blood pressure; dBP, diastolic blood pressure.
(TIF)

S11 Fig Change in sBP at 12 months including aggregate data from studies which did not contribute IPD for the primary analysis (19 studies). *Four studies containing aggregate data only: Varis et al. [17], Rinfret et al. [22], Artinian et al. [18], and Kim et al. [49]. Change in sBP from studies contributing IPD adjusted for age, sex, baseline clinic BP, and history of diabetes. Abbreviations: IPD, individual patient data; sBP, systolic blood pressure.
(TIF)

S12 Fig Primary analyses excluding all studies* which did not use a home BP threshold which was lower than clinic BP. Change in sBP at 12 months. *Patients from TASMINH1 (Verberk et al. [25] and McManus et al. [24]) and diabetics from HINTS (Bosworth et al. [26]) and TCYB (Bosworth et al. [27]) all excluded. Change in sBP adjusted for age, sex, baseline clinic BP, and history of diabetes. Abbreviations: BP, blood pressure; sBP, systolic blood pressure.
(TIF)

S13 Fig Primary analyses (ABPM) excluding all studies which randomised patients on the basis of ABPM. Change in sBP at 12 months. Change in sBP adjusted for age, sex, baseline clinic BP, history of diabetes, and level of intervention. Abbreviations: ABPM, ambulatory blood pressure monitoring; BP, blood pressure; sBP, systolic blood pressure.
(TIFF)

S14 Fig Primary analyses (ABPM) excluding all studies which randomised patients on the basis of clinic BP. Change in sBP at 12 months. Change in sBP adjusted for age, sex, baseline clinic BP, history of diabetes, and level of intervention. Abbreviations: ABPM, ambulatory blood pressure monitoring; BP, blood pressure; sBP, systolic blood pressure.
(TIFF)

S15 Fig Impact of self-monitoring of BP on the RR of uncontrolled BP at 12 months, with patients lost to follow-up assumed to have controlled BP (15 studies). RR of uncontrolled BP adjusted for age, sex, baseline clinic BP, and history of diabetes. Abbreviations: BP, blood pressure; RR, relative risk.
(TIF)

S16 Fig Impact of self-monitoring of BP on the RR of uncontrolled BP at 12 months, with patients lost to follow-up assumed to have uncontrolled BP (15 studies). RR of uncontrolled BP adjusted for age, sex, baseline clinic BP, and history of diabetes. Abbreviations: BP, blood pressure; RR, relative risk.
(TIF)

S17 Fig Impact of self-monitoring of BP on clinic sBP at 12 months, with patients who had controlled BP at baseline excluded (15 studies). Change in sBP adjusted for age, sex, baseline clinic BP, and history of diabetes. Abbreviations: BP, blood pressure; sBP, systolic blood pressure.
(TIF)

S18 Fig Studies which measured change in medication at follow-up. sBP change at 12 months analysed without adjusting for medication changes at follow-up (11 studies). Change in sBP adjusted for age, sex, baseline clinic BP, and history of diabetes. Abbreviations: BP, blood pressure; sBP, systolic blood pressure.
(TIF)

S19 Fig Studies which measured change in medication at follow-up. sBP change at 12 months analysed adjusting for medication changes at follow-up (11 studies). Change in sBP adjusted for age, sex, baseline clinic BP, history of diabetes, and medication changes at 12 months follow-up. Abbreviations: BP, blood pressure; sBP, systolic blood pressure.
(TIF)

S20 Fig Influence analysis presenting the pooled estimate of mean change in sBP at 12 months with each individual study omitted from the meta-analysis in turn. Each line indicates pooled meta-analysis results with that study omitted from the results. Abbreviation: sBP, systolic blood pressure.
(TIFF)

S21 Fig Funnel plot showing mean change in sBP at 12 months. The standard error is plotted against the mean change in sBP at 12 months. An Egger’s test of zero (*P* = 1.00) would indicate little influence of publication bias. Abbreviation: sBP, systolic blood pressure.
(TIFF)

S22 Fig sBP change plotted against medication changes at 12 months follow-up, by level of intervention (11 studies). *Test for trend using a fixed-effects linear regression model adjusted for study. †The HOMERUS and TCYB trials, and studies by Godwin et al. [43] and Leiva et al. [46], were excluded due to missing data on medication changes at follow-up. Results where a negative change in BP is associated with a positive change in number of medications suggest medication intensification may be related to improved BP at 12 months. Abbreviation: BP, blood pressure; sBP, systolic blood pressure.
(TIFF)

S23 Fig The STATA code used for the meta-analysis. The STATA code used to perform the meta-analysis and figures.
(DOCX)

## Abbreviations

ABPM: ambulatory blood pressure monitoring
BMI: body mass index
BP: blood pressure
CKD: chronic kidney disease
dBP: diastolic blood pressure
DM: diabetes mellitus
IPD: individual patient data
MI: myocardial infarction
RR: relative risk
sBP: systolic blood pressure
