# Supplementary material for: Self-monitoring of blood pressure in hypertension: A systematic review and individual patient data meta-analysis
Source: PLoS Med. 2017 Sep 19;14(9):e1002389. doi: 10.1371/journal.pmed.1002389 (PMC5604965; doi:10.1371/journal.pmed.1002389)
Supplement: S1 PRISMA Checklist — (DOC) [file pmed.1002389.s001.doc]

| **Section/topic** | **#** | **Checklist item** | **Reported on page #** |
| --- | --- | --- | --- |
| **TITLE** | | |  |
| Title | 1 | Identify the report as a systematic review, meta-analysis, or both. | Title: Self-monitoring of Blood Pressure in Hypertension: A Systematic Review and Individual Patient Data Meta-Analysis |
| **ABSTRACT** | | |  |
| Structured summary | 2 | Provide a structured summary including, as applicable: background; objectives; data sources; study eligibility criteria, participants, and interventions; study appraisal and synthesis methods; results; limitations; conclusions and implications of key findings; systematic review registration number. | Abstract  Background (background and objectives), Methods (data sources, criteria and process) Findings (no of studies and participants, synthesis and results) Conclusions (conclusions and implications of findings) |
| **INTRODUCTION** | | |  |
| Rationale | 3 | Describe the rationale for the review in the context of what is already known. | ‘Introduction’ Previous work and rational described |
| Objectives | 4 | Provide an explicit statement of questions being addressed with reference to participants, interventions, comparisons, outcomes, and study design (PICOS). | The introduction describes in the text:  P – Hypertensive patients  I - Self-monitoring of Blood Pressure  C- Conventional care  O – BP reduction and proportion at target  S - Individual patient data analysis |
| **METHODS** | | |  |
| Protocol and registration | 5 | Indicate if a review protocol exists, if and where it can be accessed (e.g., Web address), and, if available, provide registration information including registration number. | Methods (ref 15)  Tucker et al., Individual patient data meta-analysis of self-monitoring of blood pressure (BP-SMART): a protocol. BMJ open. 2015;5:008532. |
| Eligibility criteria | 6 | Specify study characteristics (e.g., PICOS, length of follow-up) and report characteristics (e.g., years considered, language, publication status) used as criteria for eligibility, giving rationale. | Supplementary Fig1 shows the flow chart for the selection of included studies  Methods include ‘Study selection’ inclusion criteria including; length of follow up, setting, intervention details and targets.  Study characteristics of the included studies is shown in Fig 1. |
| Information sources | 7 | Describe all information sources (e.g., databases with dates of coverage, contact with study authors to identify additional studies) in the search and date last searched. | Methods, paragraph 2. ‘Data sources and searches’  Medline, Embase and the Cochrane Library were searched for trials using blood pressure self-monitoring in hypertensive patients (S1&2 Fig; search date November 2014). |
| Search | 8 | Present full electronic search strategy for at least one database, including any limits used, such that it could be repeated. | Supplementary Fig 2 |
| Study selection | 9 | State the process for selecting studies (i.e., screening, eligibility, included in systematic review, and, if applicable, included in the meta-analysis). | Methods: Paragraph 3 ‘Study Selection’ |
| Data collection process | 10 | Describe method of data extraction from reports (e.g., piloted forms, independently, in duplicate) and any processes for obtaining and confirming data from investigators. | Methods: paragraph 4, ‘Data extraction and quality’ assessment |
| Data items | 11 | List and define all variables for which data were sought (e.g., PICOS, funding sources) and any assumptions and simplifications made. | Methods, paragraph 4, Data extraction and quality assessment. Published protocol Tucker et al., Individual patient data meta-analysis of self-monitoring of blood pressure (BP-SMART): a protocol. BMJ open. 2015;5:008532. |
| Risk of bias in individual studies | 12 | Describe methods used for assessing risk of bias of individual studies (including specification of whether this was done at the study or outcome level), and how this information is to be used in any data synthesis. | Results section ‘Sensitivity analyses’ (Results paragraph 8) described the sensitivity analysis, influence analysis and Egger’s test for funnel plot asymmetry.  S11- S19 Fig Sensitivity analysis  S20 Fig Influence analysis  S21 Fig Funnel plot  S3 table Assessment of Bias |
| Summary measures | 13 | State the principal summary measures (e.g., risk ratio, difference in means). | Methods, paragraph 6. data analysis  The primary outcomes were change in systolic and diastolic blood pressure at 12 months and controlled blood pressure below target at 12 months (control as defined by each trial). Analyses are reported in subgroups, by pre-specified level of self-monitoring intervention |
| Synthesis of results | 14 | Describe the methods of handling data and combining results of studies, if done, including measures of consistency (e.g., I^2^) for each meta-analysis. | Methods, paragraph 5. Data synthesis and analysis  A two-stage individual patient data meta-analysis was conducted using linear regression for continuous outcomes and logistic regression for proportions, aggregated across studies by random-effects inverse variance methods. Intention-to-treat comparisons of outcomes between the self-monitoring and comparator arms were summarised with Forest Plots using the I-squared statistic for heterogeneity. Regression models were adjusted for age, sex, baseline clinic blood pressure and diabetic status |

Page 1 of 2

| **Section/topic** | **#** | **Checklist item** | **Reported on page #** |
| --- | --- | --- | --- |
| Risk of bias across studies | 15 | Specify any assessment of risk of bias that may affect the cumulative evidence (e.g., publication bias, selective reporting within studies). | Results section ‘Sensitivity analyses’ (Results paragraph 8) described the sensitivity analysis, influence analysis and Egger’s test for funnel plot asymmetry.  S11- S19 Fig Sensitivity analysis  S20 Fig Influence analysis  S21 Fig Funnel plot  S3 table Assessment of Bias  Discussion, paragraph 9. |
| Additional analyses | 16 | Describe methods of additional analyses (e.g., sensitivity or subgroup analyses, meta-regression), if done, indicating which were pre-specified. | Sensitivity analyses, influence analysis and Eggar’s test for funnel plot asymmetry  Four post hoc analyses were carried out examining resistant hypertension, the distribution of baseline antihypertensive medications, the effectiveness of self-monitoring in stroke and the influence of blinding. |
| **RESULTS** | | |  |
| Study selection | 17 | Give numbers of studies screened, assessed for eligibility, and included in the review, with reasons for exclusions at each stage, ideally with a flow diagram. | Results section paragraph 1 summarises the number of included studies  Flow chart S1 Fig. |
| Study characteristics | 18 | For each study, present characteristics for which data were extracted (e.g., study size, PICOS, follow-up period) and provide the citations. | Table 1 |
| Risk of bias within studies | 19 | Present data on risk of bias of each study and, if available, any outcome level assessment (see item 12). | Results section ‘Sensitivity analyses’ (Results paragraph 8) described the sensitivity analysis, influence analysis and Egger’s test for funnel plot asymmetry.  S11- S19 Fig Sensitivity analysis  S20 Fig Influence analysis  S21 Fig Funnel plot  S3 table Assessment of Bias  Discussion, paragraph 9. |
| Results of individual studies | 20 | For all outcomes considered (benefits or harms), present, for each study: (a) simple summary data for each intervention group (b) effect estimates and confidence intervals, ideally with a forest plot. | Fig 1-8 and S3-8  Described in Results sections Clinic blood pressure, clinic blood pressure control and intensity of co-intervention. |
| Synthesis of results | 21 | Present results of each meta-analysis done, including confidence intervals and measures of consistency. | Fig 1-8 and S3-8  Described in Results sections Clinic blood pressure, clinic blood pressure control and intensity of co-intervention. |
| Risk of bias across studies | 22 | Present results of any assessment of risk of bias across studies (see Item 15). | Described in the Discussion paragraph 9  S11- S19 Fig Sensitivity analysis  S20 Fig Influence analysis  S21 Fig Funnel plot  S3 table Assessment of Bias |
| Additional analysis | 23 | Give results of additional analyses, if done (e.g., sensitivity or subgroup analyses, meta-regression [see Item 16]). | Sensitivity analyses described in Results section ‘Sensitivity analysis’  S2 Table and S20 |
| **DISCUSSION** | | |  |
| Summary of evidence | 24 | Summarize the main findings including the strength of evidence for each main outcome; consider their relevance to key groups (e.g., healthcare providers, users, and policy makers). | Discussion ‘Main findings’, paragraph 1 |
| Limitations | 25 | Discuss limitations at study and outcome level (e.g., risk of bias), and at review-level (e.g., incomplete retrieval of identified research, reporting bias). | Discussion: Strength and weaknesses section |
| Conclusions | 26 | Provide a general interpretation of the results in the context of other evidence, and implications for future research. | Discussion section ‘Meaning of the study’ and ‘Future research’  Discussion: conclusion and recommendations for clinical practice and future research |
| **FUNDING** | | |  |
| Funding | 27 | Describe sources of funding for the systematic review and other support (e.g., supply of data); role of funders for the systematic review. | Sources of Funding were disclosed to the journal.  This research was funded by the Institute for Health Research School for Primary Care Research (NIHR SPCR number 112) and via an NIHR Professorship for RM (NIHR-RP-02-12-015). JS holds a Medical Research Council (MRC) Strategic Skills Postdoctoral Fellowship (MR/K022032/1). FDRH is part funded as Director of the National Institute for Health Research (NIHR) School for Primary Care Research (SPCR), Theme Leader of the NIHR Oxford Biomedical Research Centre (BRC), and Director of the NIHR Collaboration for Leadership in Applied Health Research and Care (CLAHRC) Oxford. The views expressed are those of the author(s) and not necessarily those of the NIHR, the NHS or the Department of Health.  The funders had no role in study design, data collection and analysis, decision to publish, or preparation of the manuscript. |

*From:*  Moher D, Liberati A, Tetzlaff J, Altman DG, The PRISMA Group (2009). Preferred Reporting Items for Systematic Reviews and Meta-Analyses: The PRISMA Statement. PLoS Med 6(7): e1000097. doi:10.1371/journal.pmed1000097

For more information, visit: **www.prisma-statement.org**.

Page 2 of 2
