# Supplementary material for: Self-monitoring of blood pressure in hypertension: A systematic review and individual patient data meta-analysis
Source: PLoS Med. 2017 Sep 19;14(9):e1002389. doi: 10.1371/journal.pmed.1002389 (PMC5604965; doi:10.1371/journal.pmed.1002389)
Supplement: S1 Text — (PDF) [file pmed.1002389.s002.pdf]

Downloaded from http://bmjopen.bmj.com/ on October 25, 2016 - Published by group.bmj.com

BMJ Open

Individual patient data meta-analysis of self-monitoring of blood pressure (BP-SMART): a protocol

Katherine L Tucker,[1] James P Sheppard,[1] Richard Stevens,[1] Hayden B Bosworth,[2] Alfred Bove, Emma P Bray,[3,4] Marshal Godwin,[5] Beverly Green,[6] Paul Hebert,[7] F D Richard Hobbs,[1] Ilkka Kantola,[8] Sally Kerry,[9] David J Magid,[10] Jonathan Mant,[11] Karen L Margolis,[12] Brian McKinstry,[13] Stefano Omboni,[14] Olugbenga Ogedegbe,[15] Gianfranco Parati,[16] Nashat Qamar,[17] Juha Varis,[8] Willem Verberk,[18] Bonnie J Wakefield,[19] Richard J McManus[1]

To cite: Tucker KL, Sheppard JP, Stevens R, *et al*. Individual patient data meta-analysis of self-monitoring of blood pressure (BP-SMART): a protocol. *BMJ Open* 2015;**5**:e008532. doi:10.1136/bmjopen-2015-008532

▸ Prepublication history and additional material is available. To view please visit the journal (http://dx.doi.org/10.1136/bmjopen-2015-008532).

Received 17 April 2015
Revised 16 July 2015
Accepted 27 July 2015

For numbered affiliations see end of article.

Correspondence to
Professor Richard McManus; Richard.McManus@phc.ox.ac.uk

ABSTRACT

Introduction: Self-monitoring of blood pressure is effective in reducing blood pressure in hypertension. However previous meta-analyses have shown a considerable amount of heterogeneity between studies, only part of which can be accounted for by meta-regression. This may be due to differences in design, recruited populations, intervention components or results among patient subgroups. To further investigate these differences, an individual patient data (IPD) meta-analysis of self-monitoring of blood pressure will be performed.

Methods and analysis: We will identify randomised trials that have compared patients with hypertension who are self-monitoring blood pressure with those who are not and invite trialists to provide IPD including clinic and/or ambulatory systolic and diastolic blood pressure at baseline and all follow-up points where both intervention and control groups were measured. Other data requested will include measurement methodology, length of follow-up, cointerventions, baseline demographic (age, gender) and psychosocial factors (deprivation, quality of life), setting, intensity of self-monitoring, self-monitored blood pressure, comorbidities, lifestyle factors (weight, smoking) and presence or not of antihypertensive treatment. Data on all available patients will be included in order to take an intention-to-treat approach. A two-stage procedure for IPD meta-analysis, stratified by trial and taking into account age, sex, diabetes and baseline systolic BP will be used. Exploratory subgroup analyses will further investigate non-linear relationships between the prespecified variables. Sensitivity analyses will assess the impact of trials which have and have not provided IPD.

Ethics and dissemination: This study does not include identifiable data. Results will be disseminated in a peer-reviewed publication and by international conference presentations.

Conclusions: IPD analysis should help the understanding of which self-monitoring interventions for which patient groups are most effective in the control of blood pressure.

Strengths and limitations of this study

- This study will gather all available individual patient data from previous trials examining the effectiveness of self-monitoring of blood pressure in hypertension totalling up to 10 000 randomised patients.
- It will be powered to compare the effectiveness of self-monitoring in different subgroups which was not previously possible.
- This study is inherently retrospective but all proposed analyses will be agreed prior to conducting the investigation.

BACKGROUND

Across Europe and the USA, around 30% of adults have or are being treated for hypertension, which is a key risk factor for cardiovascular disease, the largest cause of death worldwide.[1–4] Treatment of hypertension through lowering blood pressure results in significant reductions in coronary artery disease and stroke.[5] Self-measurement of blood pressure (BP) has been shown in randomised controlled trials to reduce BP over and above standard care.[6 7] The improvements seen are thought to be due to increased patient involvement in their own treatment, resulting in more effective hypertension management.[8] Self-monitoring of BP is an increasingly common part of hypertension management, is well tolerated by patients and has been shown to be a better predictor of end organ damage than office measurement.[9–14]

Previous systematic reviews and meta-analyses have found that self-monitoring reduces clinic BP by an average of around 4 mm Hg for systolic pressure and by around 1.5 mm Hg for diastolic pressure, small, but

Downloaded from http://bmjopen.bmj.com/ on October 25, 2016 - Published by group.bmj.com

significant reductions compared to conventional care.[7 15 16] However, these analyses found significant heterogeneity between the studies included (Systolic I^2=71.9%, Diastolic I^2=42.1%) that could not be accounted for by meta regression.[16] Similar reductions were seen in daytime ambulatory systolic BP monitor (ABPM) but the small number of studies with such data included in the previous analysis made interpretation difficult.[16 17]

Analysis by Bray *et al* suggested that when self-monitoring was accompanied by a cointervention, participants were more likely to meet target BP but this did not explain remaining heterogeneity. Key issues in understanding this include differences in study populations such as age, gender, body mass index (BMI), a previous cardiovascular event and socioeconomic situation. Subgroup analyses from a previous summary meta-analysis suggests that the observed heterogeneity can be explained in part, due to cointerventions such as telemonitoring and use of self-titration and the setting in which the intervention is delivered.[18] Further differences in intervention, comparators and outcome measures may be important and there may be subgroups of patients for whom self-monitoring is of greater or reduced benefit.

An individual patient data meta-analysis of these data may allow better discrimination of the causes of the underlying heterogeneity.

METHODS

Aims and objectives

This study will undertake an individual patient data meta-analysis of randomised trials of self-monitoring BP using an intention-to-treat approach where possible. It will assess the evidence for the effectiveness of self-monitoring blood pressure, examine the effects of mediators of such effects and examine if particular subgroups would particularly benefit from self-monitoring intervention. In addition we will aim to develop a prospective register of trials to facilitate on going analyses.

1. The primary objectives are to estimate the effect of self-monitoring BP compared to standard care on:
   - systolic and diastolic clinic BP at 12-month follow-up
   - systolic and diastolic ambulatory BP at 12 months follow-up
   - proportion controlled below the target specified in the individual trial at 12 months follow-up

   The effects at 6 and 18 months will also be examined as the data allows as a secondary objective.
2. To use individual patient data to further explore the heterogeneity found previously and to assess the effect on outcome of the following where data allow: length of follow-up, cointerventions, baseline demographic (age, gender) and psychosocial factors (deprivation, quality of life), setting, intensity of self-monitoring intervention, comorbidities (eg, history of diabetes, cardiovascular disease, stroke), lifestyle factors (diet, exercise, weight, smoking) and presence or not of antihypertensive treatment and the number of antihypertensive medications prescribed. This will allow better definition of which intervention to use with whom so as to better operationalise implementation of self-monitoring.
3. To develop a prospective register of trials to facilitate on going meta-analysis.

Criteria for considering studies for the IPD meta-analysis

All published and unpublished controlled trials where the authors are able to provide individual patient data will be included that fulfil the following criteria:

- *Population*—patients with hypertension being managed on an outpatient basis.
- *Intervention*—self-measurement of BP without medical professional input plus or minus other cointerventions.
- *Comparator*—no organised self-measurement of BP, although there may be some ad hoc measurement which would be difficult to prevent or assess.
- *Outcome*—systolic and/or diastolic BP measured in clinic, or by daytime ambulatory measurement.
- *Study design*—randomised trial of at least 100 participants followed up for at least 24 weeks.
- *Publication Date* since 2000 (because changes in the technology used for self-monitoring make comparisons prior to this date less relevant).

Search strategy for identification of studies

Relevant electronic databases (MEDLINE, EMBASE, Cochrane Library) will be searched for articles published from 2000. The search strategy has been designed to capture all the relevant literature concerning schedules for self-monitoring of blood pressure. The MEDLINE search strategy is given in online supplementary material appendix A and searches of reference lists of all retrieved papers will be performed. Articles for inclusion will be assessed independently by two reviewers. Non-randomised designs will be excluded. Data will be extracted independently by two team members with disagreements adjudicated by a third. We will study the reference lists of included articles and ask contributing authors if they have, or are aware of any unpublished data which might be included in the review.

Trial eligibility and methodological quality assessment

All published and unpublished controlled trials will be included that assess self-measurement of BP without medical professional input, if usual care did not include organised self-monitoring and if a BP outcome was available that had been taken independently of self-measurement (clinic or ambulatory measurement).

Assessment of the quality of included trials is controversial.[19] Self-monitoring studies are generally un-blinded for obvious reasons. We will assess the quality of studies in terms of the presence of randomisation, the methodology

Downloaded from http://bmjopen.bmj.com/ on October 25, 2016 - Published by group.bmj.com

of outcome assessment, intention-to-treat analyses and attrition rates.[20] We will initially include all studies, and then perform sensitivity analyses considering the potential effect of excluding studies which may be confounded for these reasons.

Data collection

Approaches will be made to all authors of trials that meet the inclusion criteria. The following data will be requested (if available);

Trial level data

- Setting (primary or secondary care)
- Population
  - Inclusion and exclusion criteria
- Method of BP outcome measurement
  - Monitor used
  - Monitor validated?
  - Arm used
  - Number of readings used in analysis
  - Other measurement criteria, for example, were repeated readings at least 1 min apart?
- Details about randomisation
  - Allocation groups
  - Method of generation of randomisation list
  - Method of concealment of randomisation
  - Stratification factors
- Intervention
  - Details of training/education given (both for control and intervention)
  - Targets used for intervention and control groups; if not specified for control concurrent national target will be used
  - Type and frequency of self-monitoring
  - Any additional allocated intervention (ie, cointervention including telemonitoring, self-management)
  - Who titrates medication (healthcare professional/patient)
  - Timing of trial follow-up appointments
- Details about cost of intervention

Individual patient data

- Demographic details
  - Age and gender
- Medical history (specific comorbidities eg, diabetes, cardiovascular disease)
- Number of medications prescribed at baseline and follow-up
- BP readings (clinic, home and ambulatory where available)
  - Baseline
  - Follow-up
- Allocation group
- Lifestyle factors
  - Smoking
  - Alcohol consumption
  - Diet
  - Weight
  - Physical activity
- Psychosocial factors including
  - Measures of deprivation, for example, Indices of Multiple Deprivation
  - Measures of anxiety and depression
  - Measures of quality of life, for example, EQ-5D, SF-36
- Patient satisfaction
- Costs
  - Resource use
  - Consultations
  - Admissions
- Any new incidence of cardiovascular events or death
- Any clustering factors, for example, by practice

Data will be requested either in electronic or paper form and a desired format and coding will be specified. Trialists may supply data in the most convenient way open to them provided details of coding are supplied. The co-ordinating centre will ensure that data items are consistently derived, labelled and coded. Each trial group will be asked to nominate a trialist to lead in the collaboration.

Data validation strategy

Original data will be transferred and stored in a secure environment at the University of Oxford and copies will be made for use in the analyses. Trial details and summary measures will be cross-checked against published articles by two reviewers and inconsistencies will be discussed with the original trialist. Data from each trial will remain the property of each individual group.

Outcome measures

The primary outcomes will be the change in mean office systolic and diastolic blood pressure, change in ambulatory systolic and diastolic BP and proportion of patients with office BP below target between baseline and follow-up. The primary outcome will be 12 months and outcomes will also be assessed at 6 and 18 months. Reporting of outcomes in the original trial report is not an eligibility requirement provided data are available.

Data analysis

Data will be initially tabulated to include important attributes of each trial and to assess comparability, for example of treatment targets.

A two-stage procedure for IPD meta-analysis (described below) will be adopted. Handling of missing data will be by complete case analysis, with sensitivity analyses using other methods including multiple imputation if possible.

The two-stage analysis will use linear regression for continuous outcomes and logistic regression for proportions, aggregated across studies by random effects inverse variance methods. Intention-to-treat comparisons of outcomes between self-monitoring arm and comparator arm will be summarised as forest plots with I^2 statistics for

Downloaded from http://bmjopen.bmj.com/ on October 25, 2016 - Published by group.bmj.com

Table 1 Level of self-monitoring intervention

Level	Name	Description
Level 1	Self-monitoring with minimal additional contact	Self-monitoring without a text system or study phone calls. This could include one off leaflets with educational materials and initial instructions from a nurse on self-monitoring BP or a card for recording BP measurements
Level 2	Self-monitoring with automated feedback or support	Web-based or telephonic tools provide feedback or support. However, no regular 1:1 contact*
Level 3	Self-monitoring with an active intervention	Web-based or telephonic tools provide feedback or support and education offered in regular classes including on hypertension self-management, and behaviour and lifestyle modifications. This could include self-management but not regular 1:1 contact*
Level 4	Self-monitoring with significant tailored support	Individually tailored support from study personnel, pharmacist or a clinician throughout the intervention.* This could include checking BP/medication or education/lifestyle counselling and may be in person, by telephone or via electronic means

*1:1 contact or support in this context refers to contact over and above that in usual care.
BP, blood pressure.

heterogeneity. Analyses will be reported in subgroups, by level of self-monitoring intervention. This will be defined according to levels based on those previously described by Uhlig *et al*,[21] as summarised in table 1. The level of intervention examined in each included study will be agreed by the co-ordinating centre and the relevant trialists prior to conducting the analysis. Regression models used in the primary analysis will be adjusted for patient characteristics (including age and sex), baseline BP and medical history, where appropriate.

Further analyses will explore the effects of age (in 10-year age bands), sex, BMI (dichotomised around BMI of 30), baseline BP (in 10 mm Hg bands), number of medications prescribed at baseline and the presence of comorbidities at baseline (myocardial infarction, stroke, diabetes, chronic kidney disease, obesity) on mean BP change and BP control at follow-up. Exploratory analyses will be conducted (where data are available) including the use and nature of cointerventions (eg, aimed at medication adherence vs behavioural change), planned intensity of self-monitoring (ie, number of home readings), psychosocial factors (eg, deprivation, quality of life), setting and type of healthcare professional involved (eg, pharmacist vs nurse vs physician), lifestyle factors (eg, diet, smoking, alcohol consumption, physical activity) and changes in antihypertensive treatment at follow-up and the impact on mean arterial BP (MAP).

In case of non-linear relationships between the prespecified variables included in the model and outcome not detected by regression, and to further explore relationships where detected, these prespecified variables will be further investigated in an exploratory analysis examining the individual categories (quintiles in the case of continuous variables).

The potential for bias due to non-participation in the IPD will be investigated by comparing aggregate data from eligible trials with and without IPD. Notwithstanding this and the impact of the inclusion criteria (which exclude studies with small populations and/or short follow-up), publication bias for the primary outcome will be explored using Eggar's methods.[22] For included trials a complete case analysis approach will be used; sensitivity analyses will investigate other methods including, if appropriate, multiple imputation.

DISCUSSION

It is hoped that individual patient data analysis will allow a greater understanding of observed between trial heterogeneity and lead to the identification of the characteristics of both the intervention and the individuals most likely to benefit from self-monitoring of BP. This will enhance understanding of self-monitoring of BP and enable better targeted and more effective use of this intervention.

Author affiliations
[1]Nuffield Department of Primary Care, University of Oxford, Oxford, UK
[2]Center for Health Services Research in Primary Care, Durham VAMC, Durham, North Carolina, USA
[3]Department of Cardiology, Temple University School of Medicine, Philadelphia, USA
[4]School of Psychology, University of Central Lancashire, Preston, UK
[5]Family Medicine, Memorial University of Newfoundland, St. John's, Newfoundland, Canada
[6]Group Health Research Institute, Seattle, Washington, USA
[7]Department of Health Services, University of Washington School of Public Health, Washington, DC, USA
[8]Department of Medicine, Turku University Hospital, Turku, Finland
[9]Centre for Primary Care and Public Health, Queen Mary University of London, London, UK
[10]Colorado School of Public Health, University of Colorado, Denver, Colorado, USA
[11]Primary Care Unit, Department of Public Health and Primary Care, University of Cambridge, Cambridge, UK
[12]Health Partners Institute for Education and Research, Minneapolis, Minnesota, USA
[13]Centre for Population Health Sciences, University of Edinburgh, Edinburgh, Midlothian, UK
[14]Clinical Research Unit, Italian Institute of Telemedicine, Solbiate Arno, Varese, Italy

Downloaded from http://bmjopen.bmj.com/ on October 25, 2016 - Published by group.bmj.com

[15]Division of Health and Behavior, Department of Population Health, Center for Healthful Behavior Change, New York University, Langone School of Medicine, New York, USA
[16]Departments of Cardiology, and Clinical Medicine and Prevention, University of Milano Bicocca, Milan, Italy
[17]Primary Care Clinical Sciences, University of Birmingham, Birmingham, UK
[18]Departments of Internal Medicine, Cardiovascular Research Institute Maastricht, Maastricht University Maastricht, The Netherlands
[19]Department of Veterans (VA) Health Services Research, Development Centre for Comprehensive Access and Delivery Research and Evaluation (CADRE), Iowa City VA Medical Centre, University of Iowa, Iowa City, Iowa, USA

Twitter Follow Stefano Omboni at @iitelemed and Richard J McManus at @RichardJMcManus

Contributors The manuscript was prepared by KLT and RJM and edited by all authors, the methods and analysis plan was designed by RS, JPS, SK, KLT and RJM, and reviewed by all authors. All authors have read and reviewed the manuscript.

Funding This research was funded by the National Institute for Health Research School for Primary Care Research (NIHR SPCR number 112) and via an NIHR Professorship for RJMcM (NIHR-RP-02–12-015). JS holds a Medical Research Council (MRC) Strategic Skills Postdoctoral Fellowship. The views expressed are those of the author(s) and not necessarily those of the NIHR, the NHS or the Department of Health.

Competing interests The author(s) declare that they have no competing interests apart from: RJMcM has received research funding in terms of blood pressure monitors from Omron and Lloyds Healthcare. He has received expenses and an honorarium from the Japanese Society of Hypertension. FDRH has in the past received limited free or subsidised BP measuring devices from Microlife and Omron to support hypertension research, where there is no input from the companies to the design, funding, delivery, analysis or interpretation of that research. SO is a consultant of Biotechmed Ltd. (provider of blood pressure telemonitoring services). SK has received research funding in terms of blood pressure monitors from Omron.

Ethics approval This is a protocol paper for the analysis of existing trial data. Each study in the individual patient data set will have received local ethical approval. The planned study will not require further recruitment of patients and the analysis will not include identifiable data.

Provenance and peer review Not commissioned; externally peer reviewed.

Open Access This is an Open Access article distributed in accordance with the terms of the Creative Commons Attribution (CC BY 4.0) license, which permits others to distribute, remix, adapt and build upon this work, for commercial use, provided the original work is properly cited. See: http://creativecommons.org/licenses/by/4.0/

REFERENCES

1. World Health Organization. Cardiovascular diseases. Fact Sheet N° 317. 2007. http://www.who.int/nmh/publications/fact_sheet_cardiovascular_en.pdf (globaly CV are the leading cause of death—particularly ihd and stroke). (updated January 2011).
2. World Health Organization. The top 10 causes of death. 2011. http://www.who.int/mediacentre/factsheets/fs310/en/index.html (ischemic heart disease and stroke—leading causes of death in the world).
3. Adiyaman A, Tosun N, Elving LD, *et al*. The effect of crossing legs on blood pressure. *Blood Press Monit* 2007;12:189–93.
4. Association AH. 2012. http://www.heart.org/HEARTORG/Conditions/HighBloodPressure/UnderstandYourRiskforHighBloodPressure/Understand-Your-Risk-for-High-Blood-Pressure_UCM_002052_Article.jsp
5. Collins R, Peto R, MacMahon S, *et al*. Blood pressure, stroke, and coronary heart disease. Part 2, Short-term reductions in blood pressure: overview of randomised drug trials in their epidemiological context. *Lancet* 1990;335:827–38.
6. McManus RJ, Mant J, Roalfe A, *et al*. Targets and self monitoring in hypertension: randomised controlled trial and cost effectiveness analysis. *BMJ* 2005;331:493.
7. Cappuccio FP, Kerry SM, Forbes L, *et al*. Blood pressure control by home monitoring: meta-analysis of randomised trials. *BMJ* 2004;329:145. Epub 2004/06/15.
8. McManus RJ, Glasziou P, Hayen A, *et al*. Blood pressure self monitoring: questions and answers from a national conference. *BMJ* 2008;337:a2732.
9. Little P, Barnett J, Barnsley L, *et al*. Comparison of acceptability of and preferences for different methods of measuring blood pressure in primary care. *BMJ* 2002;325:258–9.
10. Bobrie G, Chatellier G, Genes N, *et al*. Cardiovascular prognosis of "masked hypertension" detected by blood pressure self-measurement in elderly treated hypertensive patients. *JAMA* 2004;291:1342–9.
11. Fagard RH, Van Den Broeke C, De Cort P. Prognostic significance of blood pressure measured in the office, at home and during ambulatory monitoring in older patients in general practice. *J Hum Hypertens* 2005;19:801–7.
12. Asayama K, Ohkubo T, Kikuya M, *et al*. Prediction of stroke by home "morning" versus "evening" blood pressure values: the Ohasama study. *Hypertension* 2006;48:737–43.
13. Niiranen TJ, Hanninen MR, Johansson J, *et al*. Home-measured blood pressure is a stronger predictor of cardiovascular risk than office blood pressure: the Finn-Home study. *Hypertension* 2010;55:1346–51.
14. Ohkubo T, Imai Y, Tsuji I, *et al*. Home blood pressure measurement has a stronger predictive power for mortality than does screening blood pressure measurement: a population-based observation in Ohasama, Japan. *J Hypertens* 1998;16:971–5.
15. Fahey T, Schroeder K, Ebrahim S. Interventions used to improve control of blood pressure in patients with hypertension. *Cochrane Database Syst Rev* 2005;(1):CD005182.
16. Bray EP, Holder R, Mant J, *et al*. Does self-monitoring reduce blood pressure? Meta-analysis with meta-regression of randomized controlled trials. *Ann Med* 2010;42:371–86.
17. Omboni S, Gazzola T, Carabelli G, *et al*. Clinical usefulness and cost effectiveness of home blood pressure telemonitoring: meta-analysis of randomized controlled studies. *J Hypertens* 2013;31:455–67; discussion 67–8.
18. Agarwal R, Bills JE, Hecht TJ, *et al*. Role of home blood pressure monitoring in overcoming therapeutic inertia and improving hypertension control: a systematic review and meta-analysis. *Hypertension* 2011;57:29–38.
19. Juni P, Witschi A, Bloch R, *et al*. The hazards of scoring the quality of clinical trials for meta-analysis. *JAMA* 1999;282:1054–60.
20. Heneghan C, Ward A, Perera R, *et al*. Self-monitoring of oral anticoagulation: systematic review and meta-analysis of individual patient data. *Lancet* 2012;379:322–34.
21. Uhlig K, Patel K, Ip S, *et al*. Self-measured blood pressure monitoring in the management of hypertension: a systematic review and meta-analysis. *Ann Intern Med* 2013;159:185–94.
22. Egger M, Davey Smith G, Schneider M, *et al*. Bias in meta-analysis detected by a simple, graphical test. *BMJ* 1997;315:629–34.

Downloaded from http://bmjopen.bmj.com/ on October 25, 2016 - Published by group.bmj.com

BMJ Open

Individual patient data meta-analysis of self-monitoring of blood pressure (BP-SMART): a protocol

Katherine L Tucker, James P Sheppard, Richard Stevens, Hayden B Bosworth, Alfred Bove, Emma P Bray, Marshal Godwin, Beverly Green, Paul Hebert, F D Richard Hobbs, Ilkka Kantola, Sally Kerry, David J Magid, Jonathan Mant, Karen L Margolis, Brian McKinstry, Stefano Omboni, Olugbenga Ogedegbe, Gianfranco Parati, Nashat Qamar, Juha Varis, Willem Verberk, Bonnie J Wakefield and Richard J McManus

BMJ Open 2015 5:
doi: 10.1136/bmjopen-2015-008532

Updated information and services can be found at:
http://bmjopen.bmj.com/content/5/9/e008532

These include:

Supplementary Material Supplementary material can be found at:
http://bmjopen.bmj.com/content/suppl/2015/09/15/bmjopen-2015-008532.DC1.html

References This article cites 18 articles, 5 of which you can access for free at:
http://bmjopen.bmj.com/content/5/9/e008532#BIBL

Open Access This is an Open Access article distributed in accordance with the terms of the Creative Commons Attribution (CC BY 4.0) license, which permits others to distribute, remix, adapt and build upon this work, for commercial use, provided the original work is properly cited. See: http://creativecommons.org/licenses/by/4.0/

Email alerting service Receive free email alerts when new articles cite this article. Sign up in the box at the top right corner of the online article.

Errata An erratum has been published regarding this article. Please see next page or:
http://bmjopen.bmj.com/content/6/5/e008532corr1.full.pdf

Topic Collections Articles on similar topics can be found in the following collections

Cardiovascular medicine (639)
Evidence based practice (585)
General practice / Family practice (535)

To request permissions go to:
http://group.bmj.com/group/rights-licensing/permissions

To order reprints go to:
http://journals.bmj.com/cgi/reprintform

To subscribe to BMJ go to:
http://group.bmj.com/subscribe/

Downloaded from http://bmjopen.bmj.com/ on October 25, 2016 - Published by group.bmj.com

Notes

To request permissions go to:
http://group.bmj.com/group/rights-licensing/permissions

To order reprints go to:
http://journals.bmj.com/cgi/reprintform

To subscribe to BMJ go to:
http://group.bmj.com/subscribe/

Correction

Tucker KL, Sheppard JP, Stevens R, *et al*. Individual patient data meta-analysis of self-monitoring of blood pressure (BP-SMART): a protocol. *BMJ Open* 2015;5:e008532.

The affiliation for Professor Gianfranco Parati, a co-author on this paper, is incorrect and should be: Department of Cardiovascular, Neural and Metabolic Sciences, San Luca Hospital, Istituto Auxologico Italiano, Milan, Italy & Department of Medicine and Surgery, University of Milano-Bicocca, Milan, Italy.

BMJ Open 2016;**6**:e008532corr1. doi:10.1136/bmjopen-2015-008532corr1
