# Supplementary material for: Self-monitoring of blood pressure in hypertension: A systematic review and individual patient data meta-analysis
Source: PLoS Med. 2017 Sep 19;14(9):e1002389. doi: 10.1371/journal.pmed.1002389 (PMC5604965; doi:10.1371/journal.pmed.1002389)
Supplement: S1 Table — Levels used to describe the included self-monitoring interventions. * 1:1 contact or support in this context refers to contact over and above that in usual care. Abbreviation: BP, blood pressure. (DOCX) [file pmed.1002389.s003.docx]

**S1 Table.** Level of self-monitoring intervention

| Level | Name | Description |
| --- | --- | --- |
| Level 1 | Self-monitoring with minimal additional contact | Self-monitoring without a text system or study phone calls. This could include one off leaflets with educational materials and initial instructions from a nurse on self-monitoring BP or a card for recording BP measurements. |
| Level 2 | Self-monitoring with automated feedback or support | Web based or telephonic tools provide feedback or support. But no regular 1:1 contact.* |
| Level 3 | Self-monitoring with an active intervention | Web based or telephonic tools provide feedback or support and education offered in regular classes including on hypertension self-management, and behaviour and lifestyle modifications. This could include self-management but not regular 1:1 contact.* |
| Level 4 | Self-monitoring with significant tailored support | Individually tailored support from study personnel, pharmacist or a clinician throughout the intervention.* This could include checking BP / medication or education/ lifestyle counselling and may be in person, by telephone or via electronic means. |

BP = Blood pressure

* 1:1 contact or support in this context refers to contact over and above that in usual care.
