# Supplementary material for: Self-monitoring of blood pressure in hypertension: A systematic review and individual patient data meta-analysis
Source: PLoS Med. 2017 Sep 19;14(9):e1002389. doi: 10.1371/journal.pmed.1002389 (PMC5604965; doi:10.1371/journal.pmed.1002389)
Supplement: S2 Table — *Data were available from trials including 7,138/8,292 (86%) of patients randomised. +Data at 6 months follow-up were available from 8,563/12,822 (67%) of patients randomised. Abbreviation: IPD, individual patient data. (DOCX) [file pmed.1002389.s004.docx]

**S2 Table.** Studies not included in the IPD analysis

| Lead author | Year published | No. randomised | Follow-up (months) | Complete cases |
| --- | --- | --- | --- | --- |
| Artinian | 2007 | 387 | 12 | 336 |
| Baque | 2005 | 1325 | 6 | 1057 |
| Madsen | 2008 | 236 | 6 | 223 |
| Magid | 2011 | 326 | 6 | 283 |
| Marques-Contreras | 2006 | 250 | 6 | 226 |
| Marques-Contreras | 2009 | 1104 | 6 | 921 |
| Marques-Contreras | 2012 | 232 | 6 | 209 |
| Rinfret | 2009 | 223 | 12 | 147 |
| Rudd | 2004 | 150 | 6 | 137 |
| Varis | 2010 | 104 | 12 | 72 |
| Kim | 2014 | 440 | 6,12,18 | 372 (12M)  383 (6M) |
| Hosseininasab | 2014 | 196 | 6 | 190 |
| Total number of not included patients with  6m follow-up^+^ |  | **4259** |  | **3629** |
| Total number of not included patients with primary outcome* |  | **1154** |  | **927** |

*Data were available from trials including 7138/8292 (86%) of patients randomised.

^+^ Data at six months follow-up were available from 8563/12822(67%) patients randomised.
