# Supplementary material for: Self-monitoring of blood pressure in hypertension: A systematic review and individual patient data meta-analysis
Source: PLoS Med. 2017 Sep 19;14(9):e1002389. doi: 10.1371/journal.pmed.1002389 (PMC5604965; doi:10.1371/journal.pmed.1002389)
Supplement: S3 Table — *Due to the nature of the intervention, the participants in all studies were aware that they were in the self-monitoring group. (DOCX) [file pmed.1002389.s005.docx]

| **S3 Table.** Assessment of Bias | | | | | | | |
| --- | --- | --- | --- | --- | --- | --- | --- |
| **Study** | **Bias domain** | **Selection bias** | | **Performance bias** | **Detection bias** | **Attrition bias** | **Reporting bias** |
|  | **Source of bias** | **Random sequence generation** | **Allocation concealment** | **Blinding of personnel assessing outcome?** | **Methodology of outcome assessment** | **Proportion outcome data available**  **(primary outcome from paper)** | **Method of analysis** |
| Halme/Kantola (2005) | | Randomised, random number tables. | Sealed numbered opaque envelopes. | Blinded. | Mean of two office measurements using study validated automated monitor. | Randomised: 269  Data available at 6m: 231 (86%) | Complete case analysis. |
| McManus (2005)  TASMINH1 | | Randomised, computer generated. | Sealed numbered opaque envelopes. | Unblinded. | 3 study readings with electronic automated validated monitor. | Randomised: 441  Data available at 12m: 400 (91%) | Intention to treat, complete case analysis. |
| Bosworth (2007)  HINTS | | Randomised, computer generated. | Sealed numbered opaque envelopes. | Blinded. | 2 BP measurements using a digital sphyg with set protocol. | Randomised: 593  Data available at 12m: 523 (88%) | Intention to treat. |
| Verberk (2007)  HOMERUS | | Randomised, computer generated. | Sealed numbered opaque envelopes. | Blinded. | Average daytime ABPM and 3 consecutive office BP readings with validated automated study device. | Randomised: 517 **additional recruitment following publication.*  Data available at 12m: 434 (84%) | Intention to treat. |
| Green (2008)  eBP | | Randomised, computer generated. | Sealed numbered opaque envelopes. | Blinded. | 3 readings using a validated automated monitor. Mean of 2^nd^ and 3^rd^ readings used in analysis. | Randomised: 778  Data available at 12m: 730 (94%) | Intention to treat. |
| Bosworth (2009)  TCYB | | Randomised, computer generated. | Sealed numbered opaque envelopes. | Blinded. | 2 BP readings using a validated automated monitor. | Randomised:636  Data available at 12m: 504 (79%).  **Of trial arms used in BP-SMART 371 remained out of 476 randomised (78%)* | Intention to treat. |
| Parati and Omboni (2009) TeleBPcare | | Randomised, computer generated. | Internet randomisation. | Unblinded. | 2 BP readings on the study device, a validated automated monitor. | Randomised: 329  Data available at 6m: 288 (88%) | Intention to treat. |
| Earle (2010) | | Randomised, computer generated. | Sealed envelopes | Unblinded | 3 readings using a validated automated monitor. Mean of 2nd and 3rd readings used in analysis. | Randomised: 137  Data available at 6m: 95 (69%) | Intention to treat. |
| Godwin (2010) | | Randomised, computer generated. | Telephone randomisation. | Blinded. | 24h ABPM and BP readings using a validated automated monitor. | Randomised:552  Data available at 12m: 458 (83%) | Intention to treat analysis with multiple imputation. |
| McManus (2010) TASMINH2 | | Randomised, computer generated. | Internet randomisation with phone back up. | Unblinded. | 6 readings using a validated automated monitor. Mean of 2^nd^ and 3^rd^ readings used in analysis. | Randomised: 527  Data available at 12m: 480 (91%) | Intention to treat, complete case analysis |
| Hebert (2011) | | Randomised, computer generated. | Sealed numbered opaque envelopes. | Blinded. | 2 readings using a validated automated BP monitor. Second reading used for analysis. | Randomised: 416  Data available at 9m: 300 (72%) | Intention to treat, complete case analysis. |
| Wakefield (2011) | | Envelopes shuffled in Batches of 30. | Sealed numbered Opaque envelopes. | Unblinded. | 2 BP readings taken by researcher using an automated validated monitor as per JNC guidelines. | Randomised: 302  Data available at 12m: 246 (81%) | Intention to treat analysis With multiple imputation. |
| Bove (2013) HTN | | Randomised, computer generated. | Consecutive random numbers, no concealment | Unblinded. | 2 BP readings. | Randomised: 241  Data available at 6m: 206 (85%) | Complete case. |
| Kerry (2013) | | Randomised, computer generated. | Sealed numbered opaque envelopes. | Blinded. | 3 BP readings taken using a validated automated monitor. Mean 2^nd^ and 3^rd^ readings used in analysis. | Randomised: 381  Data available at 12m: 337 (88%) | Intention to treat. |
| Magid (2013) | | Randomised, computer generated. | Sealed numbered opaque envelopes. | Blinded. | 3 BP readings using the study validated automated monitor. | Randomised: 348  Data available at 6m: 326 (94%) | Intention to treat analysis with multiple imputation. |
| Margolis (2013) Hyperlink | | Randomised, computer generated. | Cluster randomised trial (allocation based on clinic). | Unblinded. | 3 BP readings using a validated automated monitor. | Randomised:450  Data available at 12m: 388 (86%) | Intention to treat; Linear mixed models. |
| Mckinstry (2013)  HITS | | Randomised, computer generated. | Internet randomisation | Blinded. | Day time ABPM. | Randomised: 401  Data available at 6m: 359 (90%) | Intention to treat. |
| Parati and Omboni (2013) TeleBPMET | | Randomised, computer generated. | Sealed numbered opaque envelopes. | Unblinded. | 2 readings on a validated automated monitor. | Randomised: 252  Data available at 12m: 181 (72%) | Intention to treat. |
| Green (2014) eCare | | Randomised, computer generated. | Computer based. Randomisation revealed following input of baseline data. | Blinded. | 3 readings using a validated automated monitor. Mean of 2^nd^ and 3^rd^ readings used in analysis. | Randomised: 101  Data available at 6m: 90 (89%) | Compete case. |
| Leiva (2014) | | Randomised, computer generated. | coordinating centre | Blinded | 3 readings using a validated automated monitor. Mean of the 3 readings in the final analysis. | Randomised: 221  Data available at 12m: 212 (96%) | Intention to treat. |
| McManus (2014) TASMIN-SR | | Randomised, computer generated. | Internet randomisation with phone back up. | Unblinded. | 6 BP readings taken using a validated automated monitor. Mean of 2^nd^ and 3^rd^ readings used in analysis. | Randomised:552  Data available at 12m: 450 (81%) | Intention to treat approach. Complete case with multiple imputation as sensitivity analysis. |
| Ogedegbe (2014)  CAATCH | | Randomised, computer generated. | Sealed numbered opaque envelopes. | Unblinded. | 6 BP readings taken using a validated automated monitor. | Randomised: 1059  Data available at 12m: 738 (70%) | Intention to treat. |
| Stewart (2014) | | Randomised, computer generated. | sealed envelopes | Unblinded | 2 BP readings taken by pharmacist using an automated validated monitor. Mean used in analysis | Randomised: 395  Data available at 6m: 354 (90%) | Intention to treat. |
| Yi (2015) | | Randomised, computer generated. | Automated system within the electronic health record | Unblinded | Readings taken from electronic health records | Randomised: 900  Data available at 9m: 661 (73%) | Intention to treat |
| Parati AUPRES (unpublished – protocol provided) | | Randomised, computer generated. | Sealed numbered opaque envelopes. | Unblinded. | Clinic BP using a semi-automated monitor and ABPM. | Randomised: 702 planned  Data available at 12m: 407 (58% of planned participants) | Intention to treat. |

*Due to the nature of the intervention the participants in all studies were aware that they were in the self-monitoring group
