# Supplementary material for: Self-monitoring of blood pressure in hypertension: A systematic review and individual patient data meta-analysis
Source: PLoS Med. 2017 Sep 19;14(9):e1002389. doi: 10.1371/journal.pmed.1002389 (PMC5604965; doi:10.1371/journal.pmed.1002389)
Supplement: S4 Table — Table showing the funding of the included studies. (DOCX) [file pmed.1002389.s006.docx]

**S4 Table.** Funding provided to the included studies

| **Author/s**  **Country, year** | **Funding statement** | **Funding type** |
| --- | --- | --- |
| **Halme/ Kantola**([13](#_ENREF_13))  **Finland, 2005** | Pfizer Oy and Turku University | Public and Commercial |
| **McManus**([14](#_ENREF_14))  **UK 2005**  (TASMINH) | Funding for the study was provided under a national primary care researcher development award to RJMcM from the NHS Executive. Approved funding from Support for Science was administered via the Midlands Research Practice Consortium. | Public |
| **Bosworth**([15](#_ENREF_15))  **US, 2007**  (HINTS) | This research is supported by a grant from Veterans Affairs, Health Services Research and Development (No. IIR 04-426), a career scientist award (No. RCS 08-027) and an Established Investigator Award from American Heart Association to Dr Bosworth. ATHENA-HTN development and testing was supported in part by VA Health Services Research and Development (Nos. CPI 99-275 and IMV-04-062). | Public / charity |
| **Verberk**([16](#_ENREF_16))  **Netherlands, 2007**  (HOMERUS) | Supported by grant 945-01-043 from the Health Care Efficiency Research Program of the Netherlands Organization for Health Research and Development. | Public |
| **Green**([17](#_ENREF_17))  **US, 2008**  (eBP) | The National Heart, Lung, and Blood Institute of the National Institutes of Health (NIH): Grant R01-HL075263 | Public |
| **Bosworth**([18](#_ENREF_18))  **US, 2009**  (TCYB) | National Heart, Lung, and Blood Institute; Pfizer Foundation Health Communication Initiative; and the American Heart Association. | Public, commercial, charity |
| **Parati & Omboni**([19](#_ENREF_19))  **Italy, 2009** (TeleBPcare) | Research funds obtained from the authors Institution along with an unrestricted research grant from Boehringer Ingelheim, Italy. | Institutional /  commercial  unrestricted |
| **Earle (2010)** | This study was funded by Motorola Inc., USA. | Commercial unrestricted |
| **Godwin**([20](#_ENREF_20))  **Canada, 2010** | Funding: Heart and Stroke Foundation of Ontario (T 5345). | Charity |
| **McManus**([21](#_ENREF_21))  **UK, 2010**  (TASMINH2) | Joint funding from the Department of Health Policy Research Programme, National Coordinating Centre for Research Capacity Development, and Midlands Research Practices Consortium (MidReC). Service support costs were obtained from the Department of Health in collaboration with MidReC. The Departments of Primary Care in Birmingham and Southampton also receive funding from NIHR National School for Primary Care Research. | Public |
| **Hebert(**[**22**](#_ENREF_22)**)**  **US, 2011** | Agency for Healthcare Research and Quality (5P01HS010859-050001), and National Institutes of Health National Center for Minority Health and Health Disparities (1P60MD000270-01). | Public |
| **Wakefield(**[**23**](#_ENREF_23)**)**  **US, 2011** | Supported by the Department of Veterans Affairs, Veterans Health Administration, Health Services Research and Development (VA HSR&D) Service (No. NRI 03-312). | Public |
| **Bove(**[**24**](#_ENREF_24)**)**  **US, 2013**  (HTN) | This work was supported by a grant from the Agency for Healthcare Quality and Research. Clinical trial no. NCT00644267. | Public |
| **Kerry**([25](#_ENREF_25))  **UK, 2013** | The main study was funded by the Stroke Association (grant no. TSA 2006/05). The feasibility study was funded by The Isaac Schapera Research Trust. Recruitment to the study was supported by the English National Institute of Health Research Clinical Stroke Research Network. Omron Healthcare UK Ltd. provided monitors for control patients after the end of the trial. (Monitors for intervention patients were provided from study funds.) | Charity |
| **Magid(**[**26**](#_ENREF_26)**)**  **US, 2013** | The study was funded in part by the American Heart Association. | Charity |
| **Margolis**([27](#_ENREF_27))  **US, 2013**  (Hyperlink) | The National Heart, Lung, and Blood Institute. (R01HL090965) | Public |
| **McKinstry**([28](#_ENREF_28))  **UK, 2013**  (HITS) | The BUPA Foundation (grant No 748/G24) with additional support from the High Blood pressure Foundation and NHS Lothian. | Charity / public |
| **Parati**([29](#_ENREF_29))  **Italy, 2013** (TeleBPMET) | This work was financially supported by Bracco S.p.A. through an unconditional and unrestricted grant. | Commercial unrestricted |
| **Green**([30](#_ENREF_30))  **US, 2014**  (eCare) | The National Heart, Lung, and Blood Institute, NIH (grant RC1HL100590) | Public |
| **Leiva (2014)** | The Adherencia study was funded by the Carlos III Health Institute of the Ministry of Economy and Competitiveness of Spain (contract No PS09/01456). The Ministry had no role in the design or conduct of the study; the collection, management, analysis, or interpretation of the data; or the preparation, review, and approval of the manuscript or the decision to submit for publication. | Public |
| **McManus**([31](#_ENREF_31))  **UK 2014**  (TAMSIN-SR) | The National Institute for Health Research (NIHR) under its Programme Grants for Applied Research Programme (Grant Reference Number RP-PG 0606-1153), by the NIHR National School of Primary Care Research (NSPCR 16), and by an NIHR career development fellowship (Dr McManus). | Public |
| **Ogedegbe(**[**32**](#_ENREF_32)**)**  **US, 2014**  (CAATCH) | The National Heart, Lung, and Blood Institute (grant R01 HL78566), National Institutes of Health. Dr Fernandez was supported by an American Heart Association Heritage Afiliate Clinically Applied Research Grant and the National Institutes of Health Loan Repayment Program in Health Disparities Research. Dr Ogedegbe was supported by a grant from the National Center for Minority Health and Health Disparities (program project 2P60 MD000206), National Institutes of Health. | Public |
| **Stewart (2014)** | The HAPPY trial is funded by the Australian Government Department of Health and Ageing as part of the Fourth Community Pharmacy Agreement through the Fourth Community Pharmacy Agreement Research & Development Grants Program managed by the Pharmacy Guild of Australia. | Public |
| **Yi (2015)** | Funding is from the Robert Wood Johnson Foundation and Finding Answers: Disparities Research for Change and New York City tax levy dollars. This funding was administered by the Fund for Public Health in New York, a private non-profit organization that supports innovative initiatives of the New York City Department of Health and Mental Hygiene. The contents of this article are solely the responsibility of the authors and do not necessarily represent the official view of the funders. The New York University Center for the Study of Asian American Health is supported by the National Institute of Health/National Institute on Minority Health and Health Disparities cooperative agreement number 2P60MD000538-10. | Charity / public |
| **Parati**  **Italy (unpublished)**  (AUPRES) | Grant from the Italian Ministry of Education, Universities and Research. | Public |
