# Supplementary material for: Self-monitoring of blood pressure in hypertension: A systematic review and individual patient data meta-analysis
Source: PLoS Med. 2017 Sep 19;14(9):e1002389. doi: 10.1371/journal.pmed.1002389 (PMC5604965; doi:10.1371/journal.pmed.1002389)
Supplement: S5 Table — (DOCX) [file pmed.1002389.s007.docx]

**S5 Table.** Distribution of baseline medications by history of stroke

| **Number of meds at baseline** | **No history of stroke** | **History of stroke** |
| --- | --- | --- |
| 0 | 347 (7%) | 44 (7%) |
| 1 | 1,762 (36%) | 221 (36%) |
| 2 | 1,701 (35%) | 213 (34%) |
| 3 | 729 (15%) | 93 (15%) |
| 4 | 191 (4%) | 41 (7%) |
| >5 | 100 (2%) | 9 (1%) |
