# Supplementary material for: Self-monitoring of blood pressure in hypertension: A systematic review and individual patient data meta-analysis
Source: PLoS Med. 2017 Sep 19;14(9):e1002389. doi: 10.1371/journal.pmed.1002389 (PMC5604965; doi:10.1371/journal.pmed.1002389)
Supplement: S1 Fig — Flow diagram of the systematic review and selection of studies for the IPD. (DOCX) [file pmed.1002389.s008.docx]

**S1 Fig**. Flow diagram of the systematic search and selection of relevant studies

**Database search**

2846 unique studies identified

2,714 studies not relevant on basis of titles and abstract screening

**Full text screening**

132 studies

61 studies were not eligible following detailed review (not self-monitoring of BP in Hypertensive population)

**Potentially eligible for this review**

71 studies

12 studies before year 2000

13 studies <100 participants

9 studies followed-up for less than 6 months
1 study with ambulatory monitoring as comparator

**Studies approached to participant in IPD**

**36 studies**

**36**

3636 studies

12 study authors did not respond or were not able to collaborate

1 additional unpublished study

**Studies included in IPD**

25 studies
