# Supplementary material for: Self-monitoring of blood pressure in hypertension: A systematic review and individual patient data meta-analysis
Source: PLoS Med. 2017 Sep 19;14(9):e1002389. doi: 10.1371/journal.pmed.1002389 (PMC5604965; doi:10.1371/journal.pmed.1002389)
Supplement: S2 Fig — An example search from Medline. (DOCX) [file pmed.1002389.s009.docx]

**S2 Fig. Example Search Strategy (Medline)**

Database: Ovid MEDLINE(R) 1946 to November 2014
Only trials published since 2000 eligible.

--------------------------------------------------------------------------------
1 blood pressure monitoring, ambulatory/
2 ((blood pressure or bp) adj3 (24h or 24hr? or 24-h or 24-hr? or 24 hour? or ambulatory or determin$ or measur$ or monitoring or monitor$ or self-measur$ or self-monitor$)).tw.
3 or/1-2
4 (home or self$).tw.
5 (telemedicine or tele-medicine or telemonitor$ or tele-monitor$).mp.

6 or/4-

7 randomized controlled trial.pt.
8 controlled clinical trial.pt.
9 randomized.ab.
10 placebo.ab.
11 drug therapy.fs.
12 randomly.ab.
13 trial.ab.
14 groups.ab.
15 or/7-14
16 animals/ not (humans/ and animals/)
17 15 not 16
18 3 and 6 and 17
