# Supplementary figures and images for: Self-monitoring of blood pressure in hypertension: A systematic review and individual patient data meta-analysis
Source: PLoS Med. 2017 Sep 19;14(9):e1002389. doi: 10.1371/journal.pmed.1002389 (PMC5604965; doi:10.1371/journal.pmed.1002389)

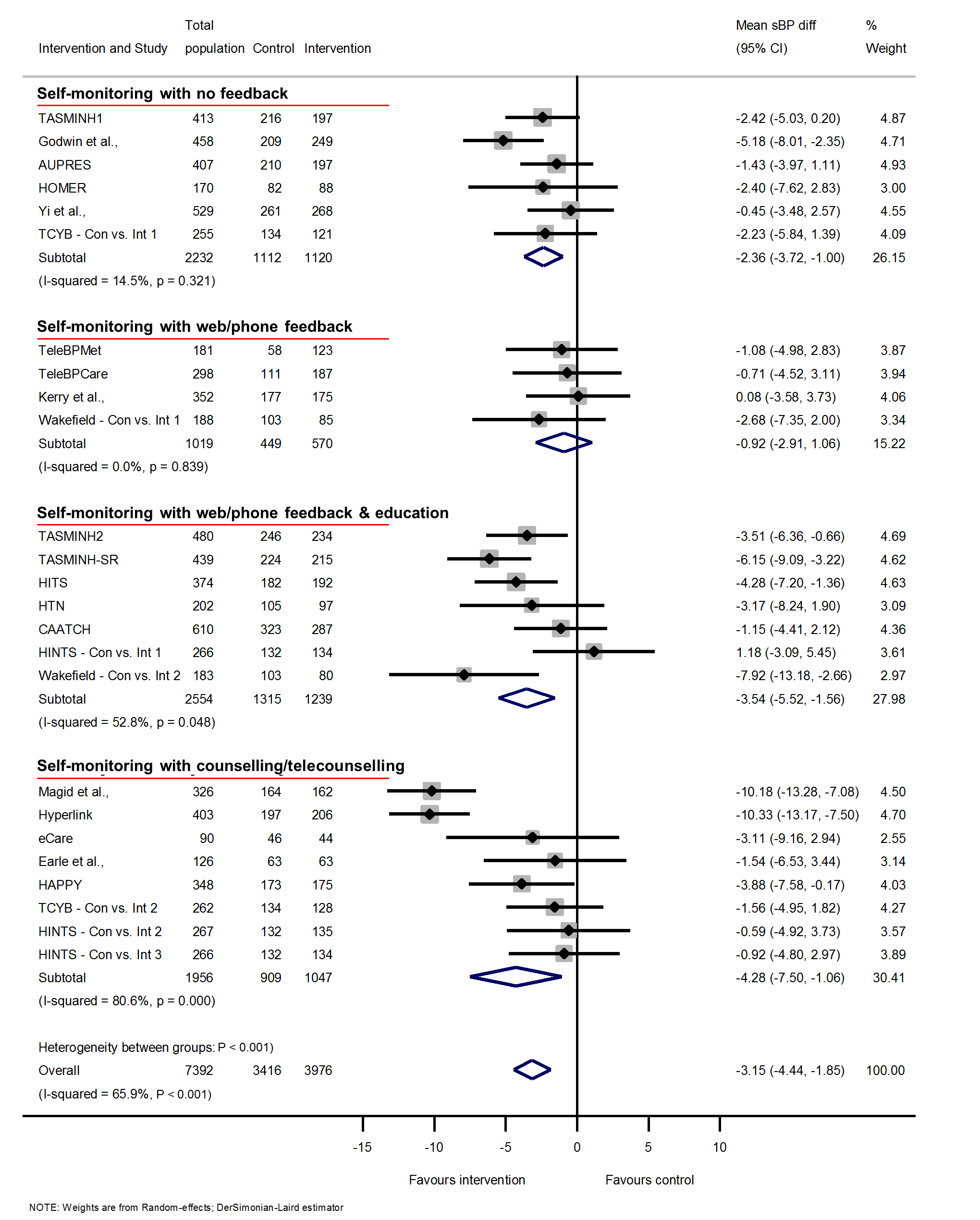

Supplement: S3 Fig — Change in sBP adjusted for age, sex, baseline clinic BP, and history of diabetes. Abbreviations: BP, blood pressure; sBP, systolic blood pressure. (TIF) [file pmed.1002389.s010.tif]

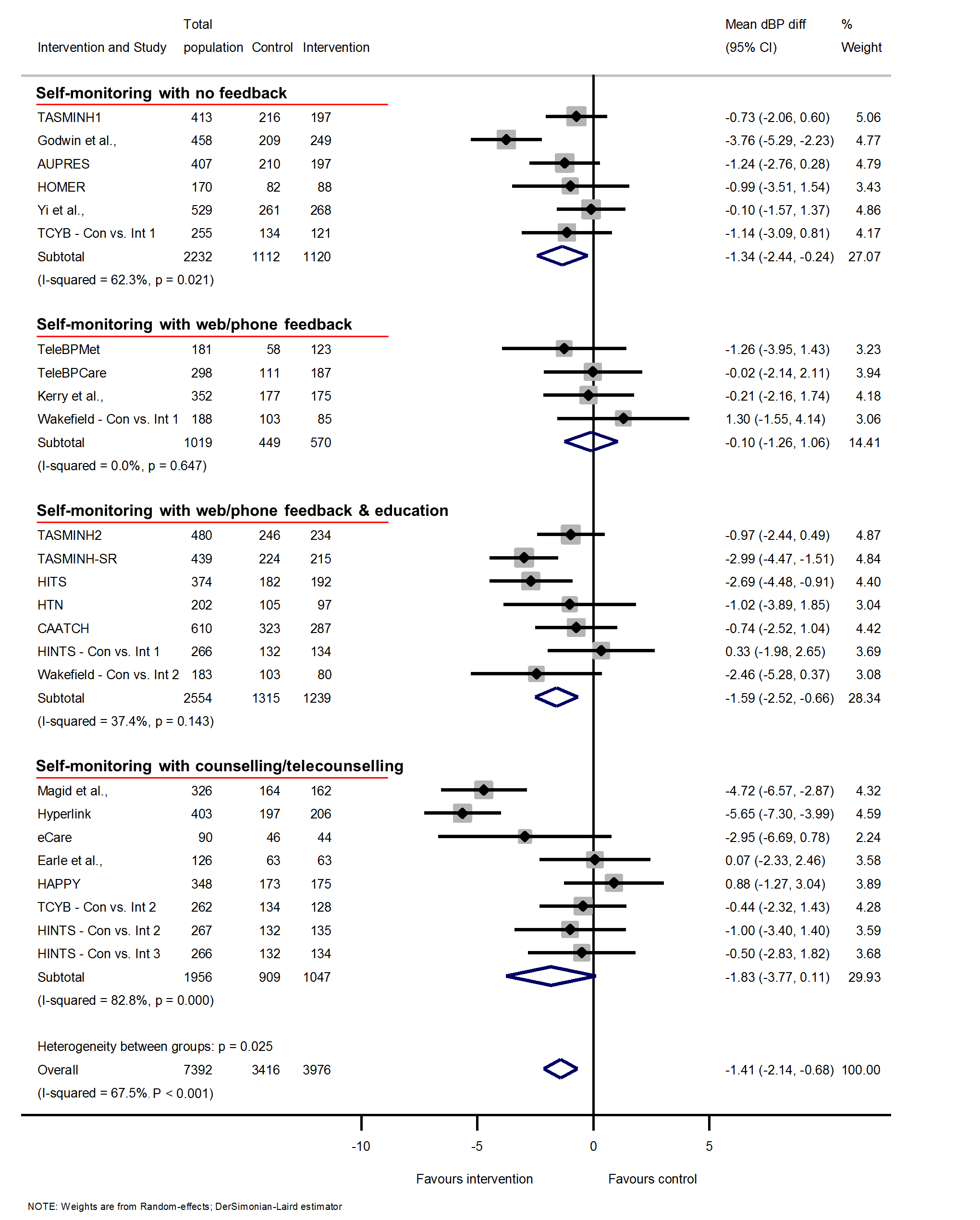

Supplement: S4 Fig — Change in dBP adjusted for age, sex, baseline clinic BP, and history of diabetes. Abbreviations: BP, blood pressure; dBP, diastolic blood pressure. (TIF) [file pmed.1002389.s011.tif]

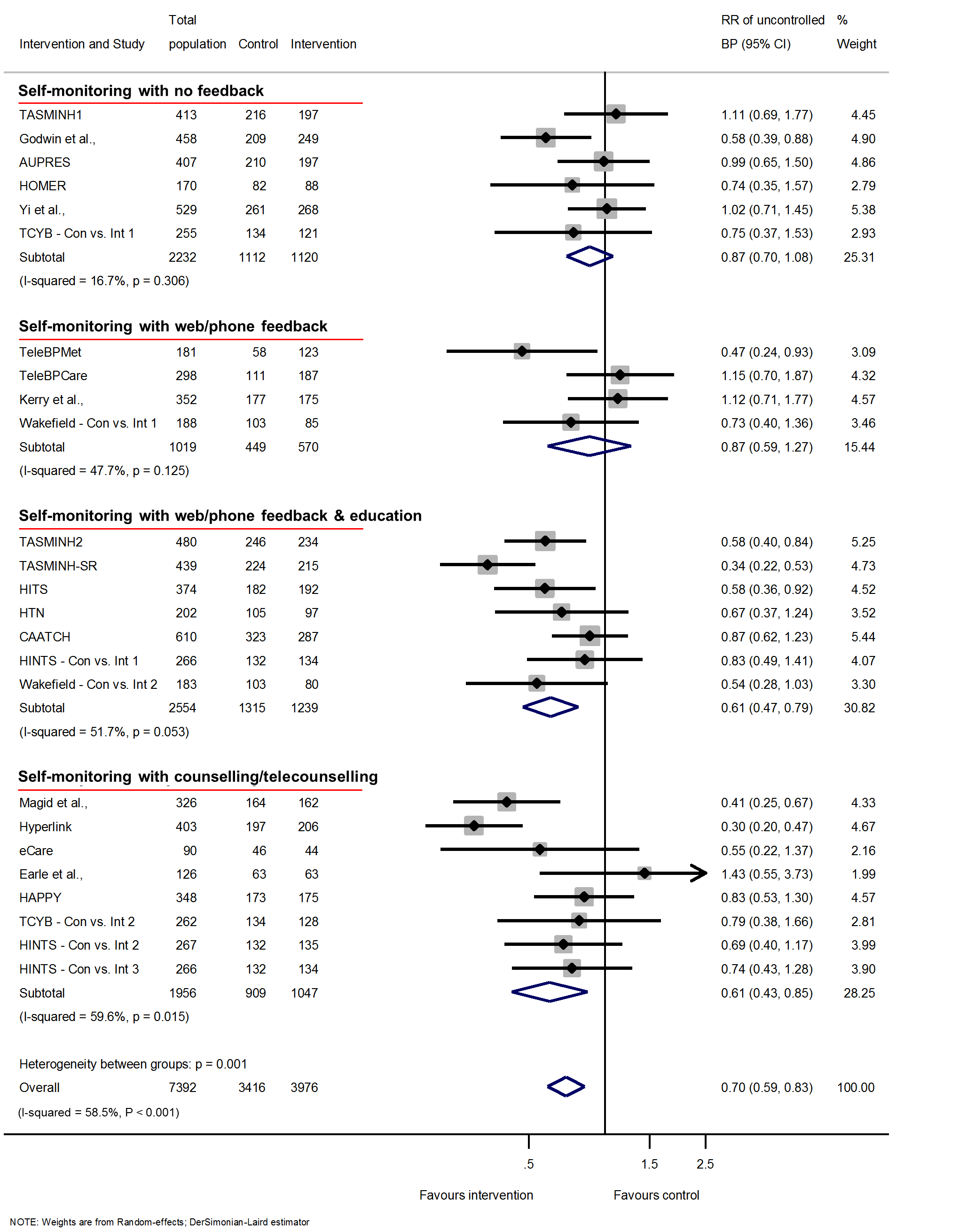

Supplement: S5 Fig — RR of uncontrolled BP adjusted for age, sex, baseline clinic BP, and history of diabetes. Abbreviations: BP, blood pressure; RR, relative risk. (TIF) [file pmed.1002389.s012.tif]

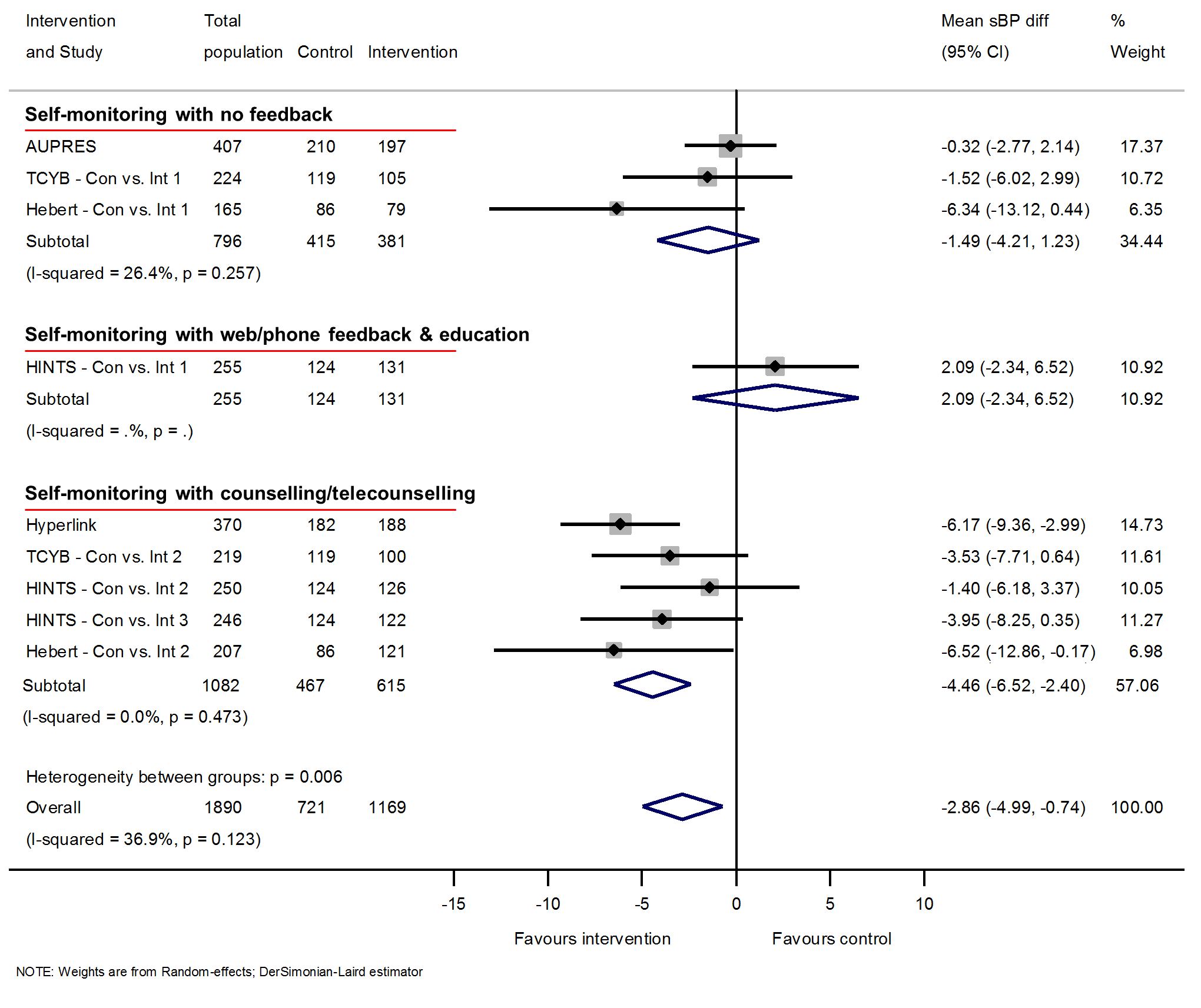

Supplement: S6 Fig — Change in sBP adjusted for age, sex, baseline clinic BP, and history of diabetes. Abbreviations: BP, blood pressure; sBP, systolic blood pressure. (TIF) [file pmed.1002389.s013.tif]

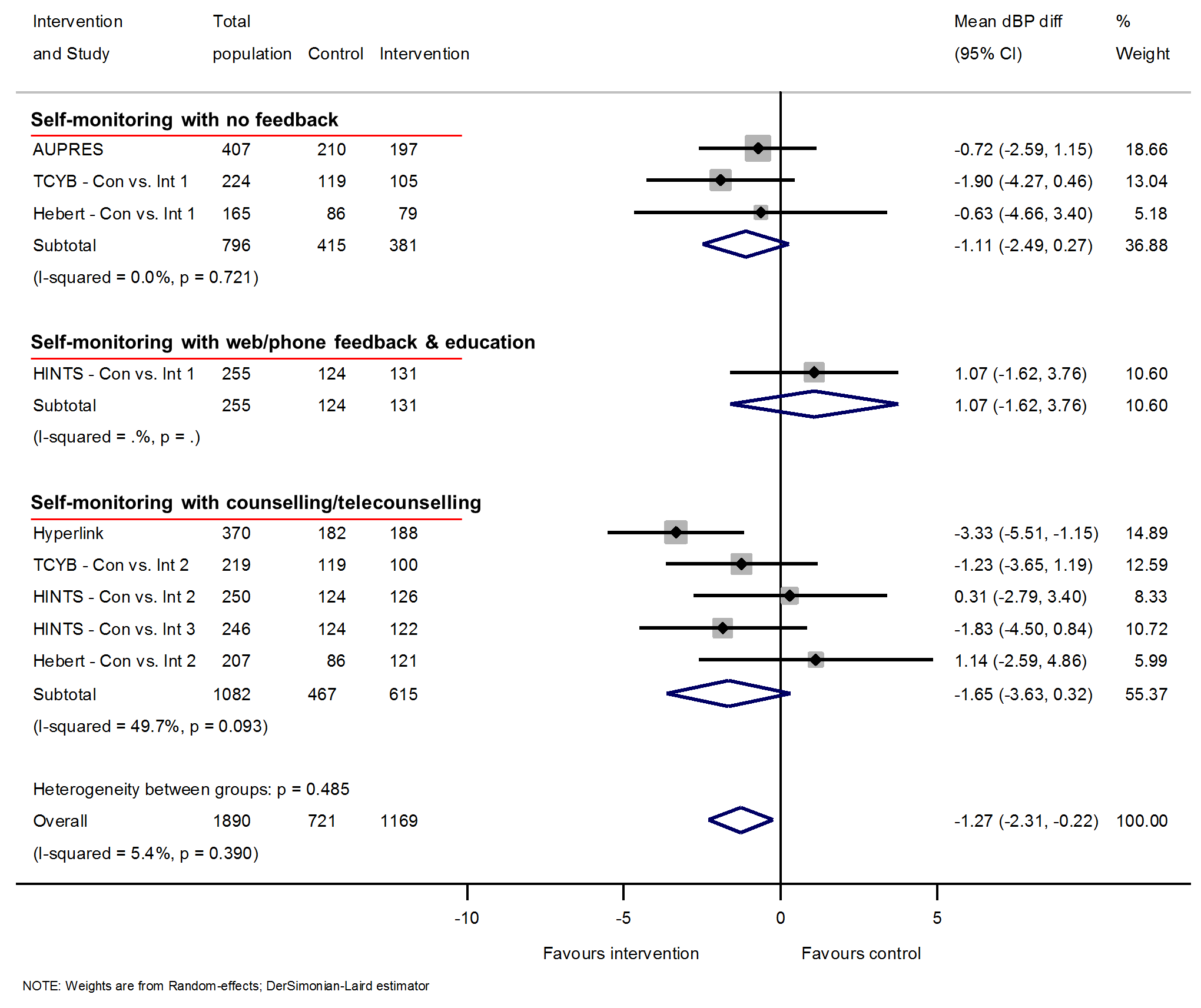

Supplement: S7 Fig — Change in dBP adjusted for age, sex, baseline clinic BP, and history of diabetes. Abbreviations: BP, blood pressure; dBP, diastolic blood pressure. (TIF) [file pmed.1002389.s014.tif]

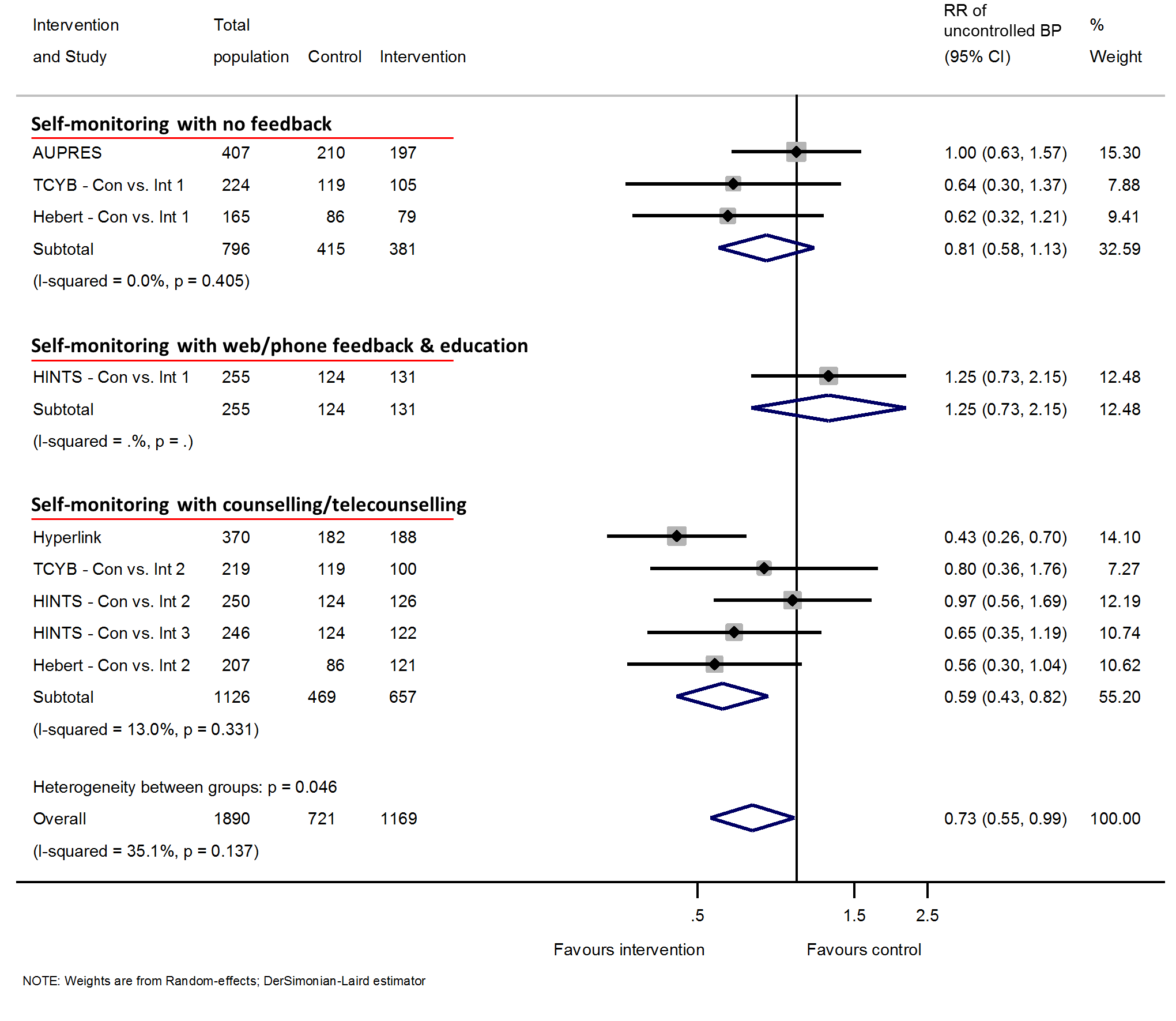

Supplement: S8 Fig — RR of uncontrolled BP adjusted for age, sex, baseline clinic BP, and history of diabetes. Abbreviations: BP, blood pressure; RR, relative risk. (TIF) [file pmed.1002389.s015.tif]

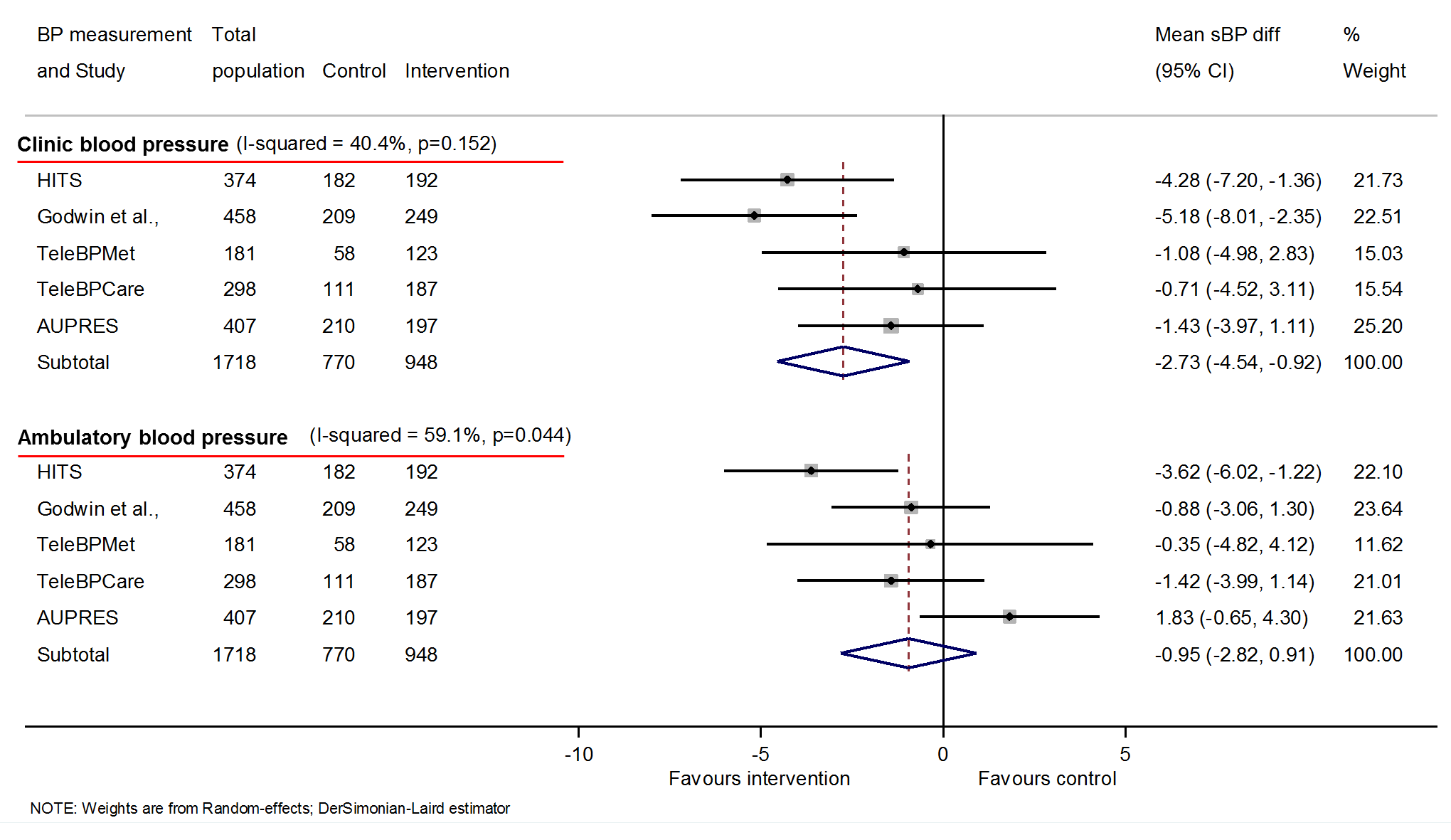

Supplement: S9 Fig — Change in sBP adjusted for age, sex, baseline clinic BP, history of diabetes, and level of intervention. Abbreviations: BP, blood pressure; sBP, systolic blood pressure. (TIF) [file pmed.1002389.s016.tif]

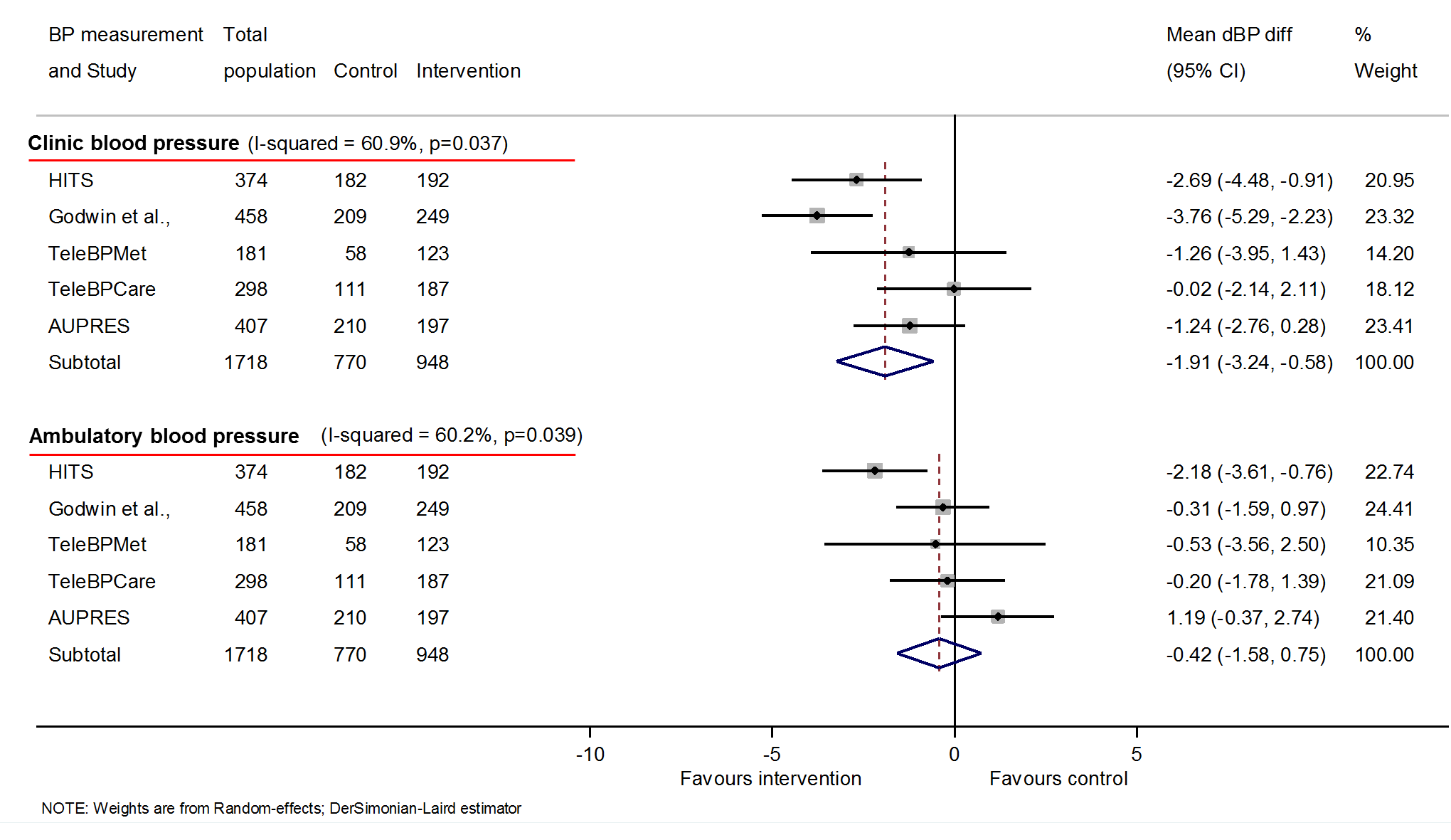

Supplement: S10 Fig — Change in dBP adjusted for age, sex, baseline clinic BP, history of diabetes, and level of intervention. Abbreviations: BP, blood pressure; dBP, diastolic blood pressure. (TIF) [file pmed.1002389.s017.tif]

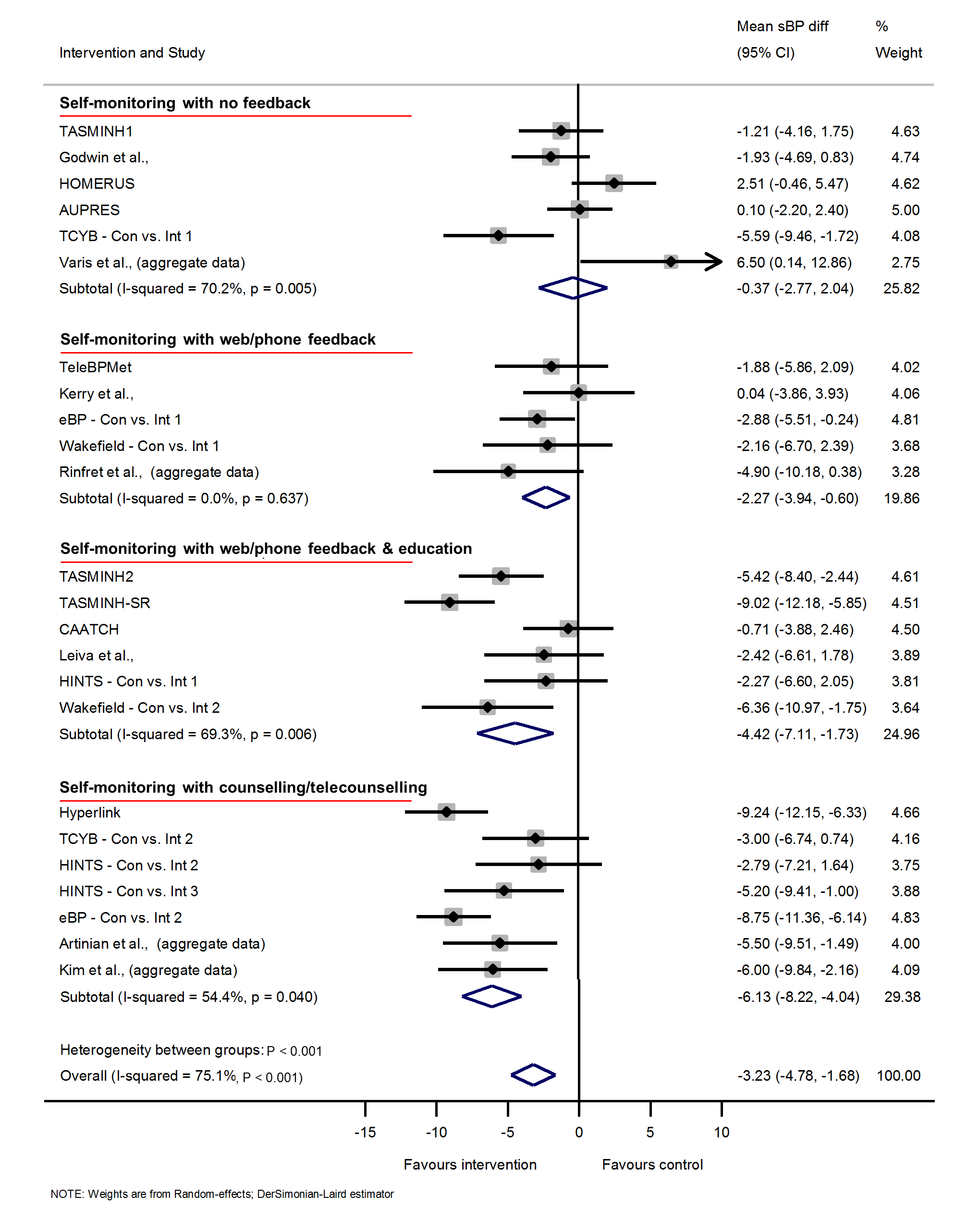

Supplement: S11 Fig — *Four studies containing aggregate data only: Varis et al. [17], Rinfret et al. [22], Artinian et al. [18], and Kim et al. [49]. Change in sBP from studies contributing IPD adjusted for age, sex, baseline clinic BP, and history of diabetes. Abbreviations: IPD, individual patient data; sBP, systolic blood pressure. (TIF) [file pmed.1002389.s018.tif]

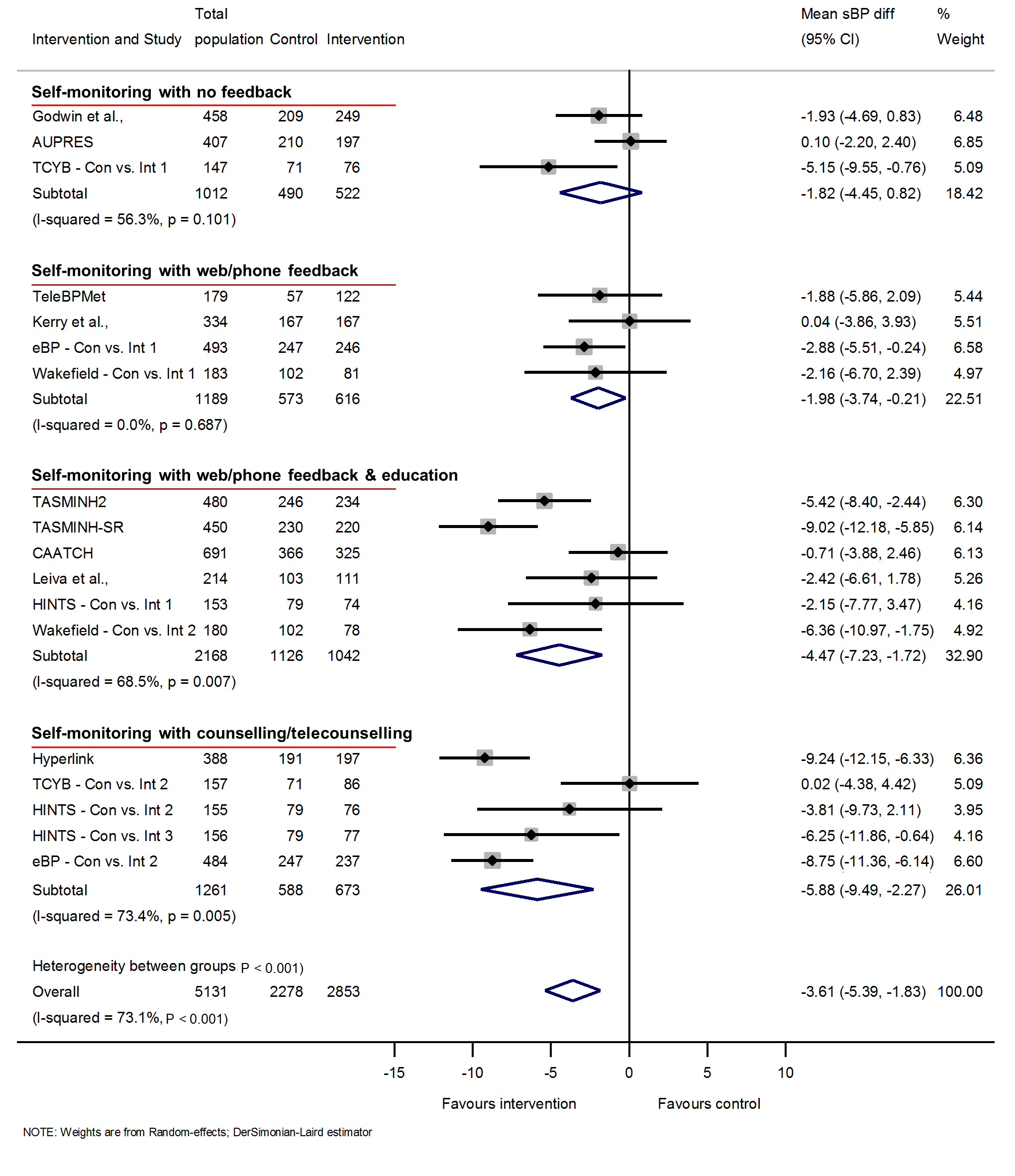

Supplement: S12 Fig — Change in sBP at 12 months. *Patients from TASMINH1 (Verberk et al. [25] and McManus et al. [24]) and diabetics from HINTS (Bosworth et al. [26]) and TCYB (Bosworth et al. [27]) all excluded. Change in sBP adjusted for age, sex, baseline clinic BP, and history of diabetes. Abbreviations: BP, blood pressure; sBP, systolic blood pressure. (TIF) [file pmed.1002389.s019.tif]

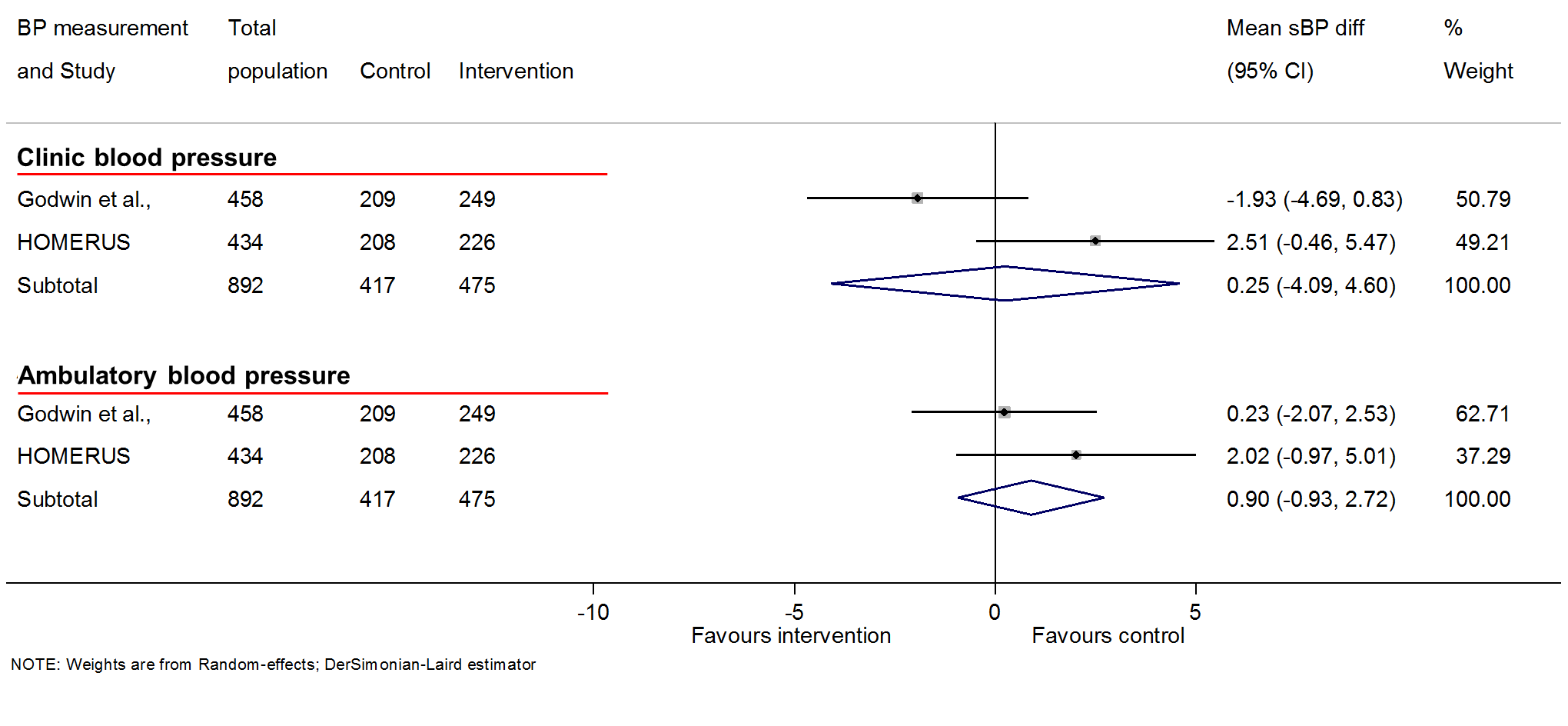

Supplement: S13 Fig — Change in sBP at 12 months. Change in sBP adjusted for age, sex, baseline clinic BP, history of diabetes, and level of intervention. Abbreviations: ABPM, ambulatory blood pressure monitoring; BP, blood pressure; sBP, systolic blood pressure. (TIFF) [file pmed.1002389.s020.tiff]

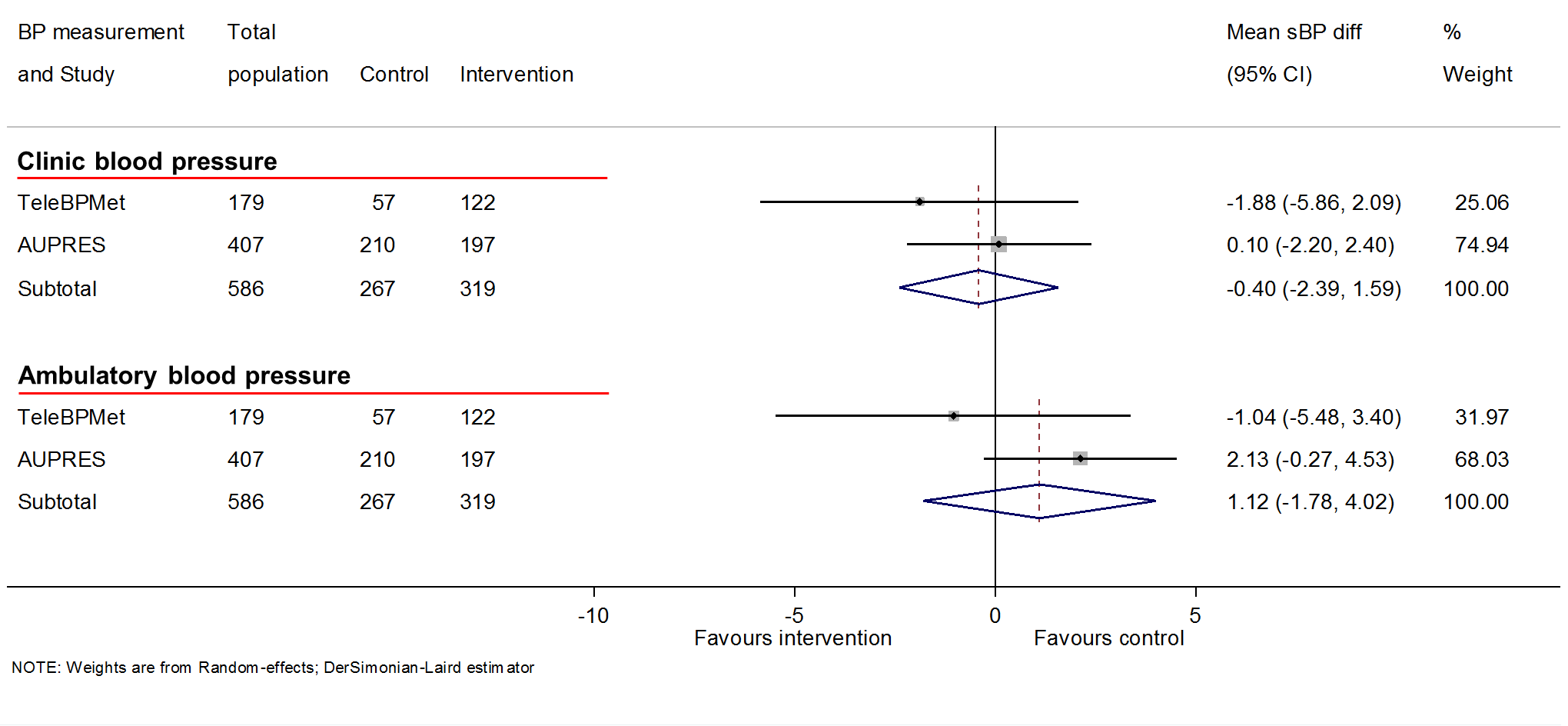

Supplement: S14 Fig — Change in sBP at 12 months. Change in sBP adjusted for age, sex, baseline clinic BP, history of diabetes, and level of intervention. Abbreviations: ABPM, ambulatory blood pressure monitoring; BP, blood pressure; sBP, systolic blood pressure. (TIFF) [file pmed.1002389.s021.tiff]

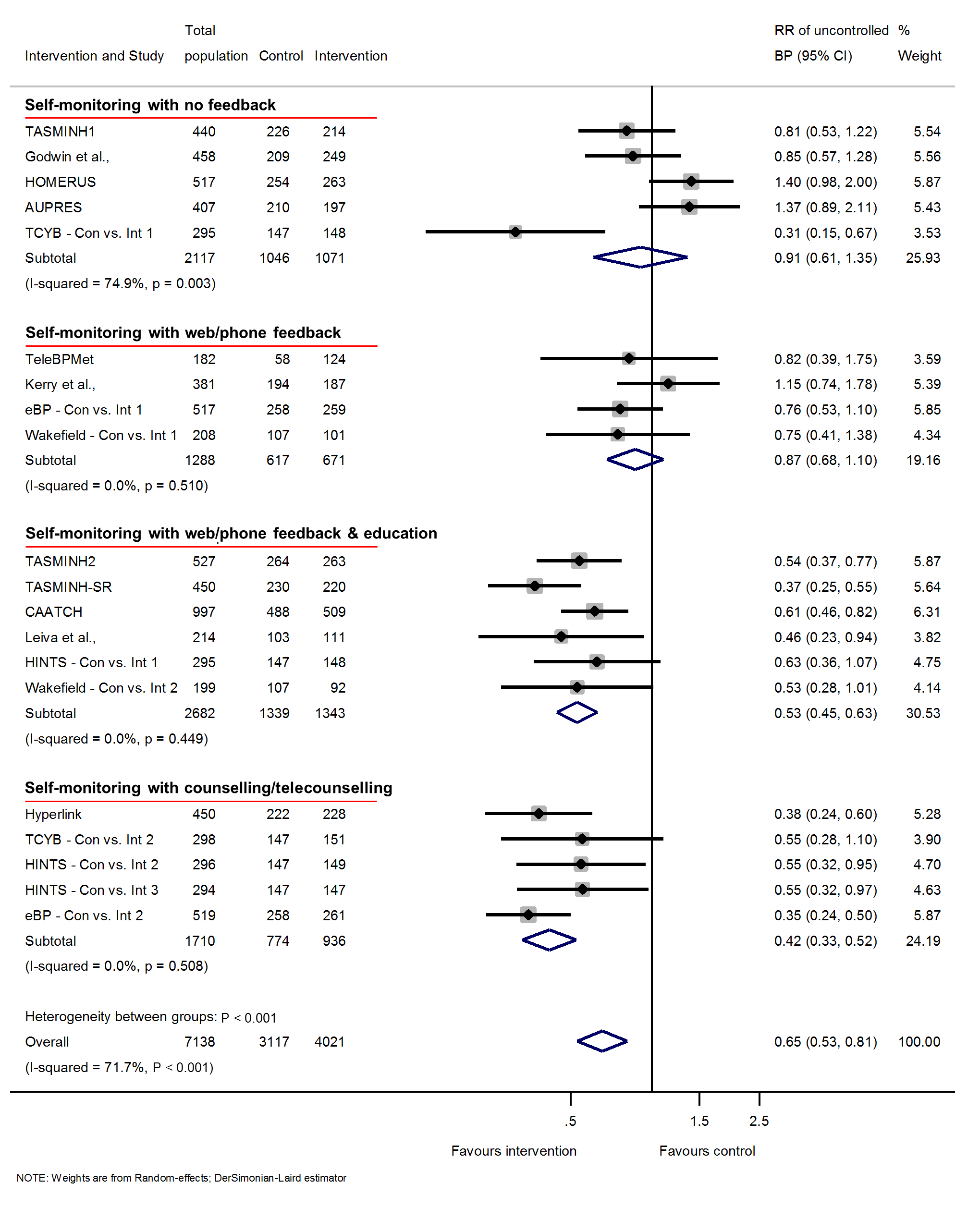

Supplement: S15 Fig — RR of uncontrolled BP adjusted for age, sex, baseline clinic BP, and history of diabetes. Abbreviations: BP, blood pressure; RR, relative risk. (TIF) [file pmed.1002389.s022.tif]

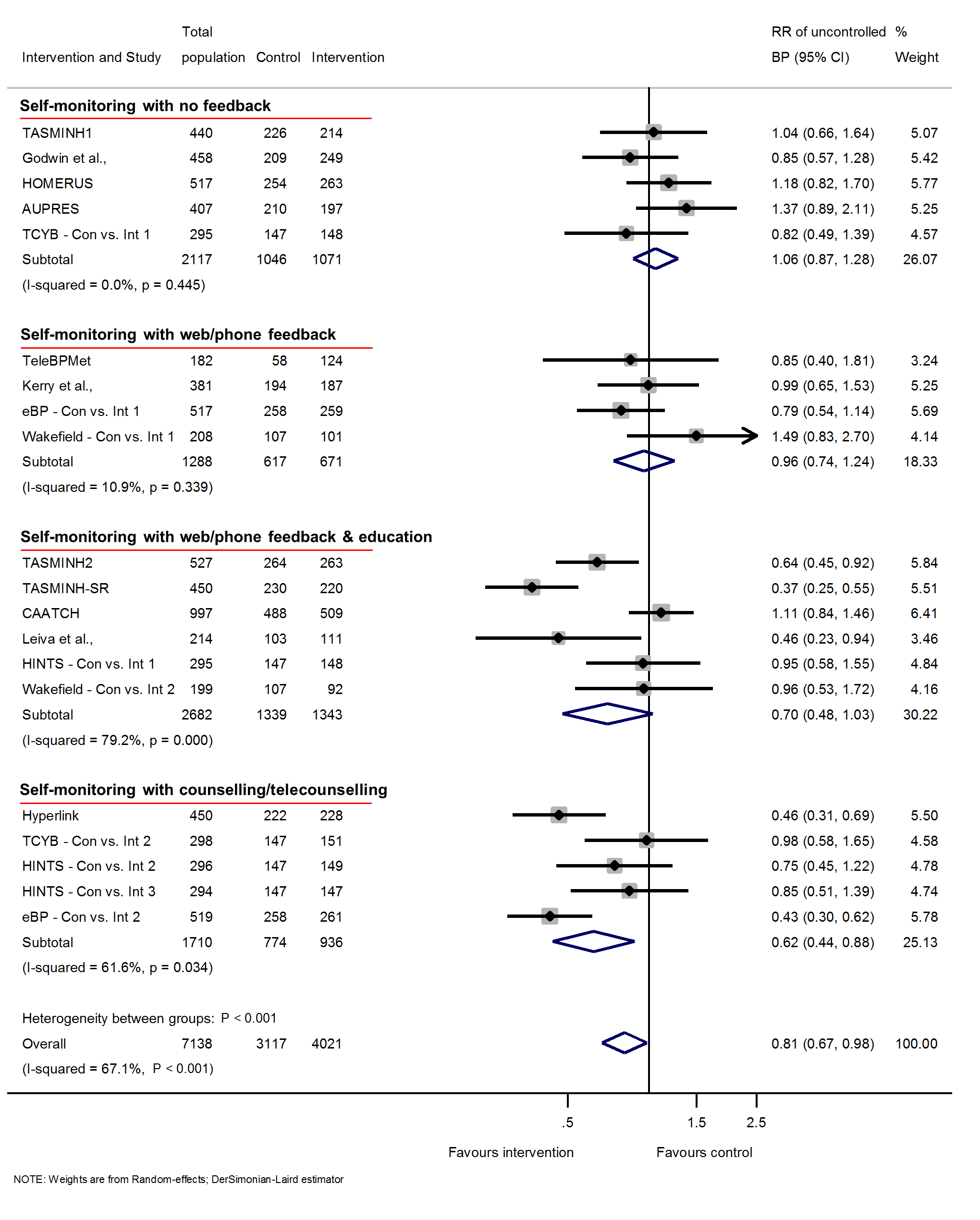

Supplement: S16 Fig — RR of uncontrolled BP adjusted for age, sex, baseline clinic BP, and history of diabetes. Abbreviations: BP, blood pressure; RR, relative risk. (TIF) [file pmed.1002389.s023.tif]

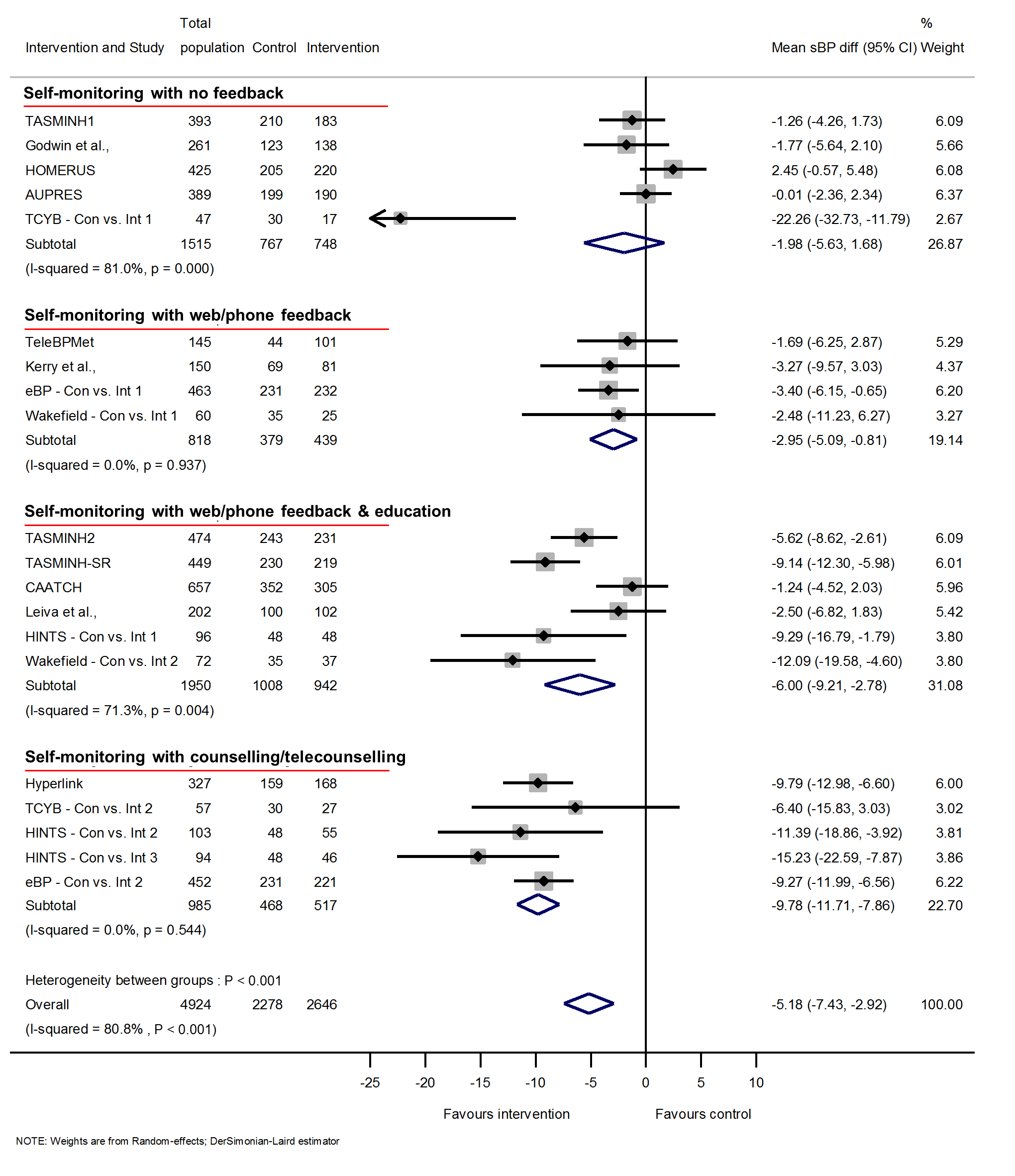

Supplement: S17 Fig — Change in sBP adjusted for age, sex, baseline clinic BP, and history of diabetes. Abbreviations: BP, blood pressure; sBP, systolic blood pressure. (TIF) [file pmed.1002389.s024.tif]

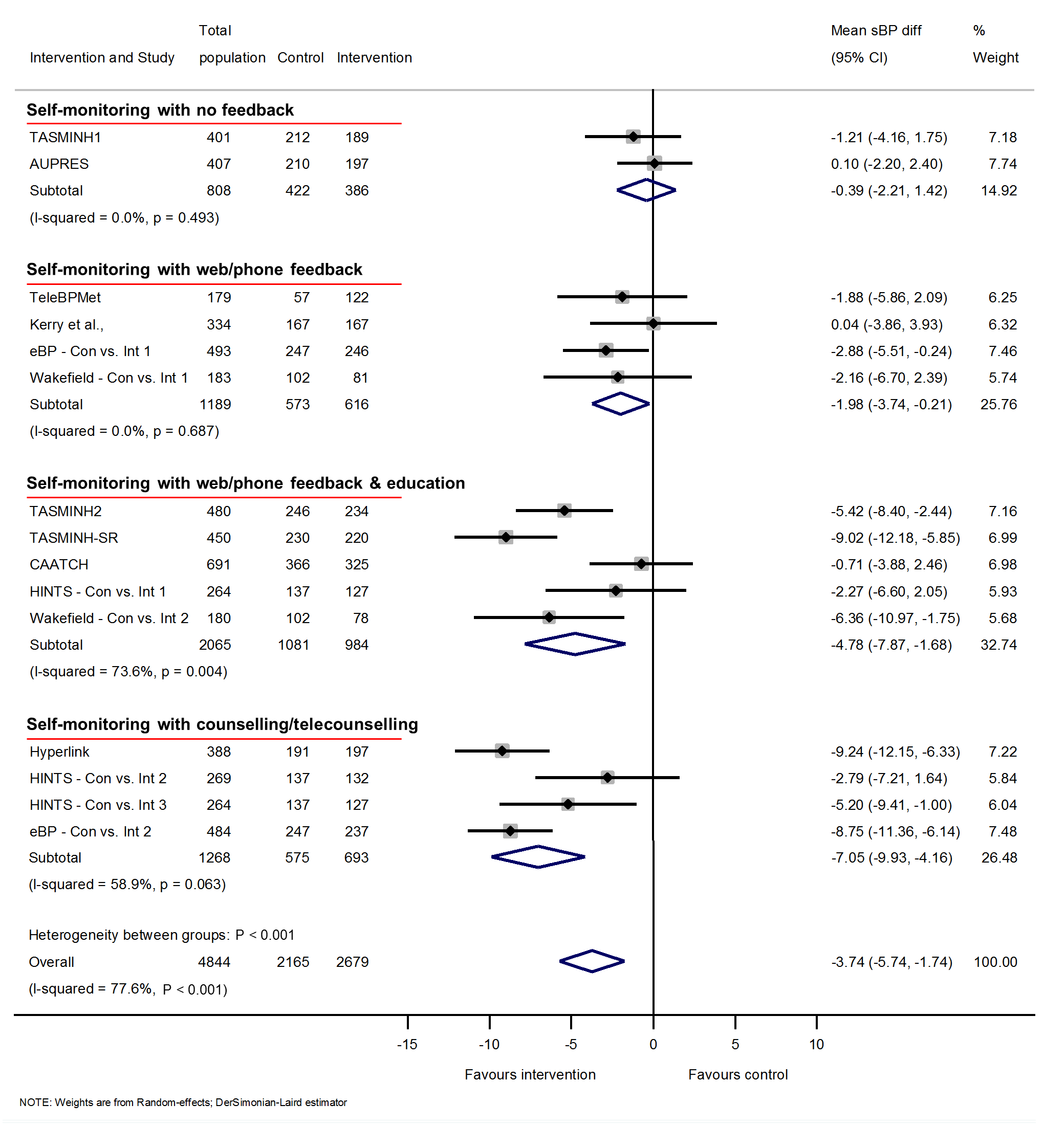

Supplement: S18 Fig — sBP change at 12 months analysed without adjusting for medication changes at follow-up (11 studies). Change in sBP adjusted for age, sex, baseline clinic BP, and history of diabetes. Abbreviations: BP, blood pressure; sBP, systolic blood pressure. (TIF) [file pmed.1002389.s025.tif]

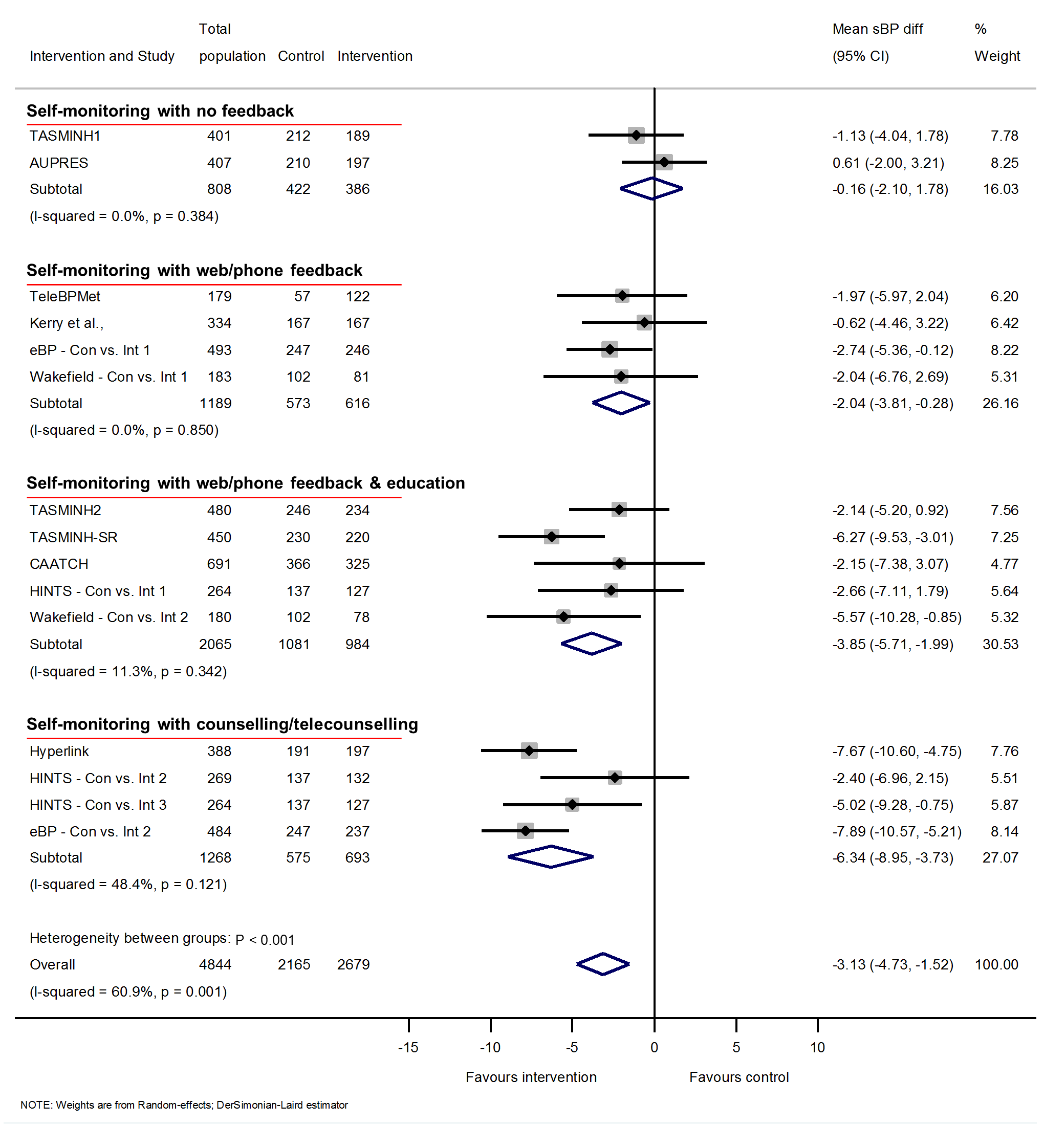

Supplement: S19 Fig — sBP change at 12 months analysed adjusting for medication changes at follow-up (11 studies). Change in sBP adjusted for age, sex, baseline clinic BP, history of diabetes, and medication changes at 12 months follow-up. Abbreviations: BP, blood pressure; sBP, systolic blood pressure. (TIF) [file pmed.1002389.s026.tif]

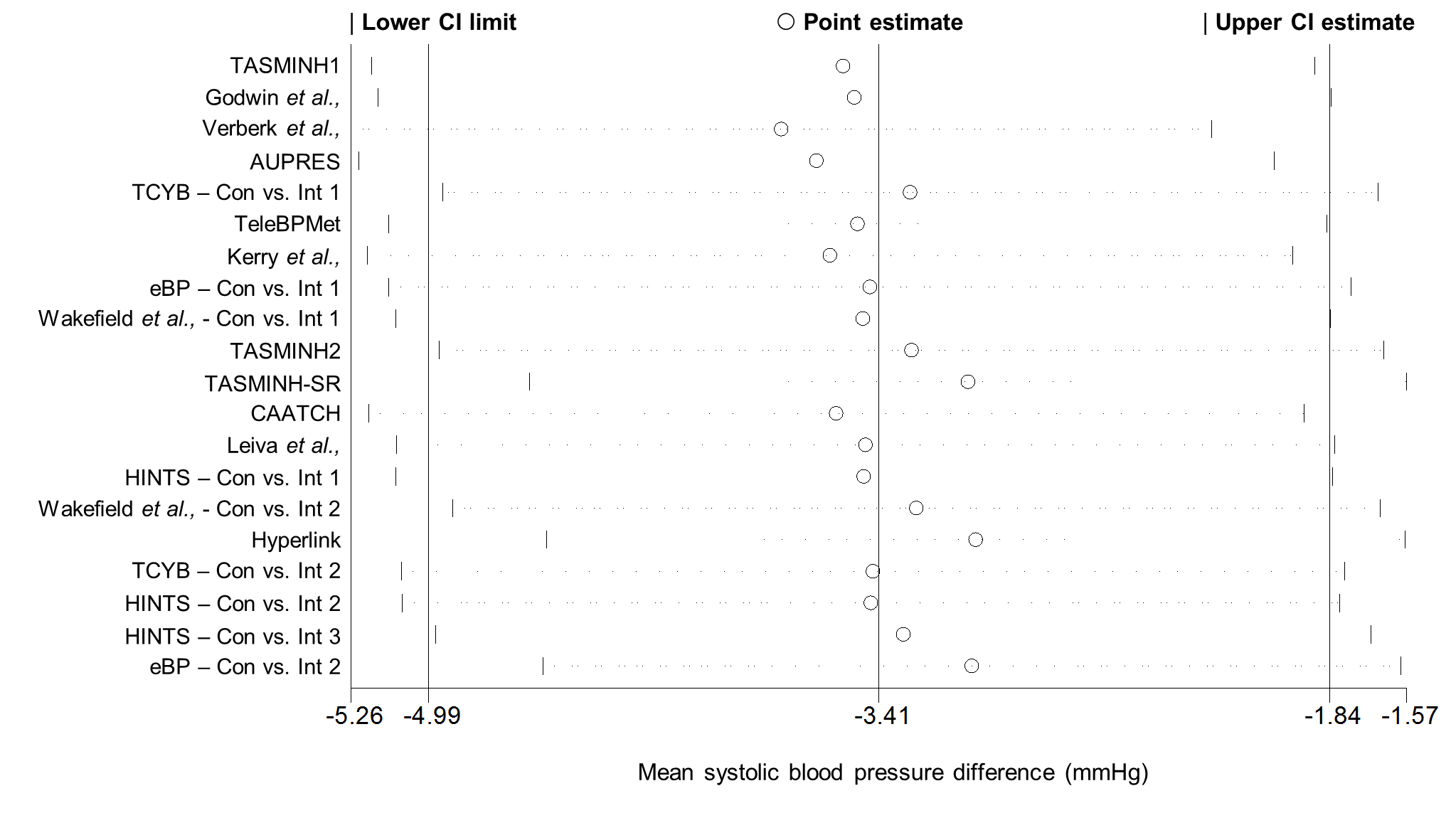

Supplement: S20 Fig — Each line indicates pooled meta-analysis results with that study omitted from the results. Abbreviation: sBP, systolic blood pressure. (TIFF) [file pmed.1002389.s027.tiff]

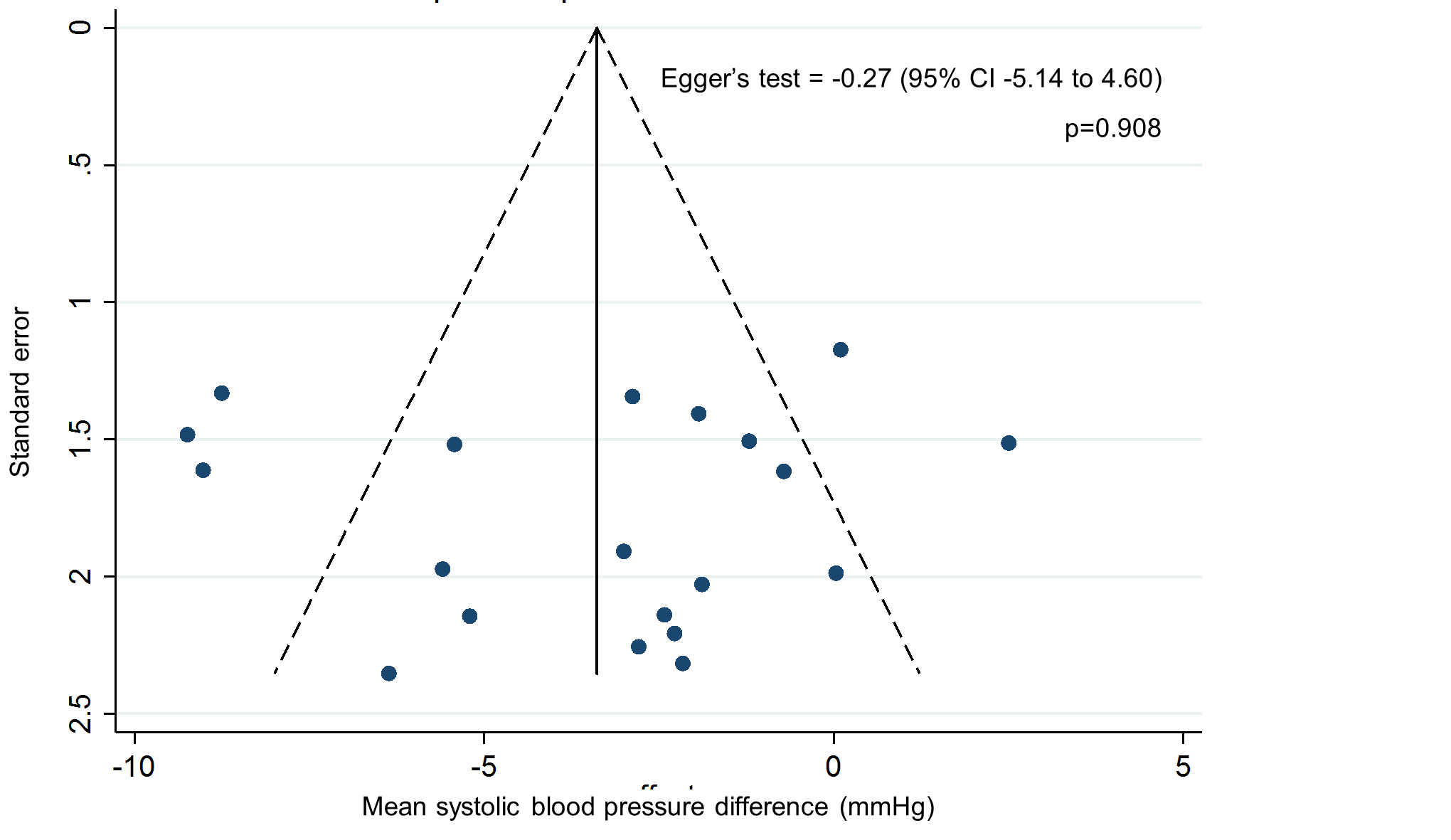

Supplement: S21 Fig — The standard error is plotted against the mean change in sBP at 12 months. An Egger’s test of zero (P = 1.00) would indicate little influence of publication bias. Abbreviation: sBP, systolic blood pressure. (TIFF) [file pmed.1002389.s028.tiff]

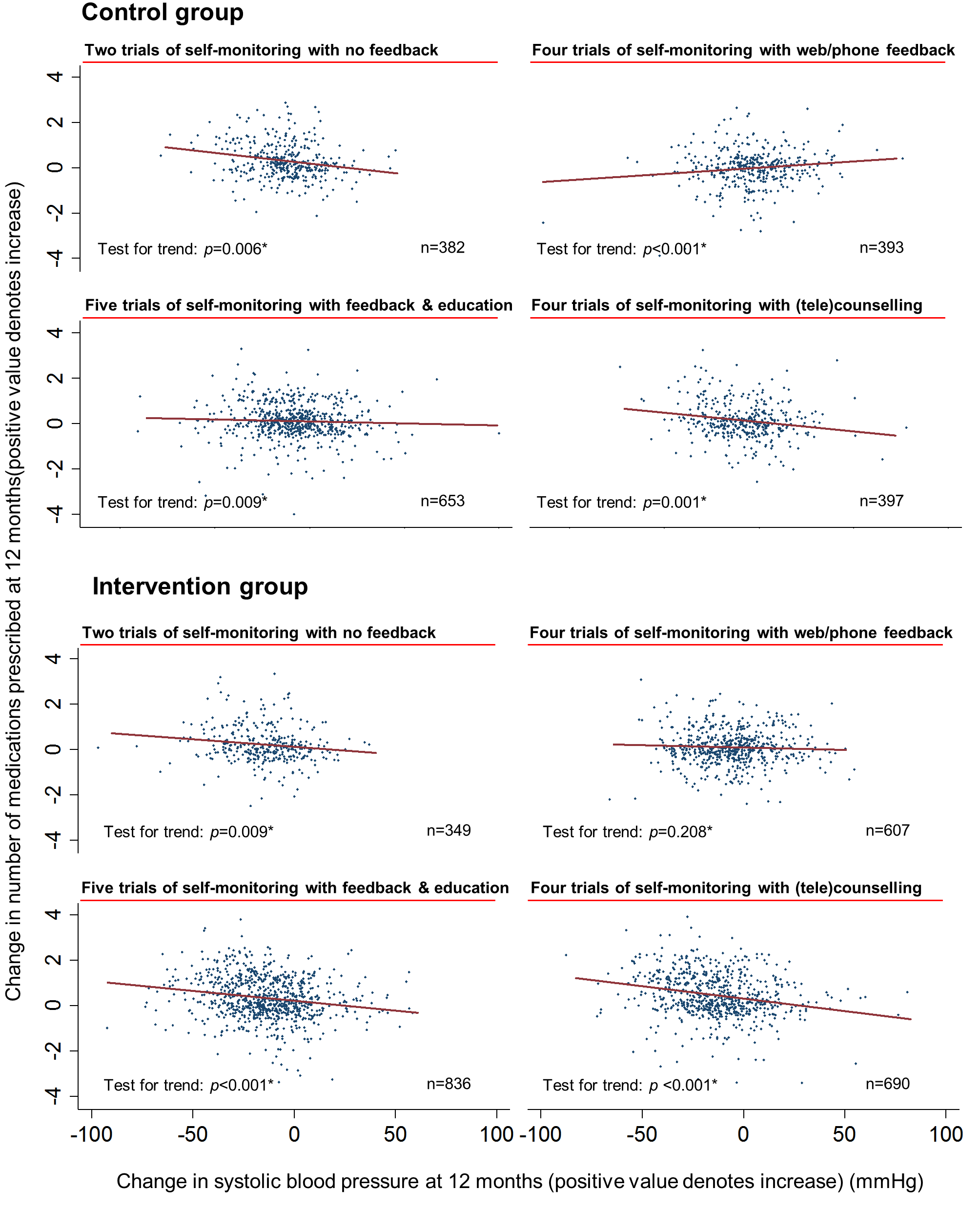

Supplement: S22 Fig — *Test for trend using a fixed-effects linear regression model adjusted for study. †The HOMERUS and TCYB trials, and studies by Godwin et al. [43] and Leiva et al. [46], were excluded due to missing data on medication changes at follow-up. Results where a negative change in BP is associated with a positive change in number of medications suggest medication intensification may be related to improved BP at 12 months. Abbreviation: BP, blood pressure; sBP, systolic blood pressure. (TIFF) [file pmed.1002389.s029.tiff]
