# Supplementary material for: Self-monitoring of blood pressure in hypertension: A systematic review and individual patient data meta-analysis
Source: PLoS Med. 2017 Sep 19;14(9):e1002389. doi: 10.1371/journal.pmed.1002389 (PMC5604965; doi:10.1371/journal.pmed.1002389)
Supplement: S23 Fig — The STATA code used to perform the meta-analysis and figures. (DOCX) [file pmed.1002389.s030.docx]

**S23 Fig.** The STATA code used for the Meta-analysis.

****Primary analyses****

************************************************IPD meta-analysis******************************************************

use "G:\SMART BP IPD\Pooled analysis\BPSMART dataset_Full.dta", clear

****************Systolic blood pressure*******************

*****IPD mean change in sBP by study and level of intevention, adjusted by age, sex and baseline BP; ************

*6 months

**Control patients for HINTS complete (for individual study point estimates)

set more off

ipdmetan, study(Study) by(level_of_intervention) effect(Mean sBP diff) re lcols((count) Total_population_6m (sum) Control_6m Intervention_6m) ///

forest(graphregion(color(white)) graphregion(margin(zero)) favours(Favours intervention # Favours control) texts(105) astext(60) xlabel(-15(5)10) range(-15,10) ///

noadjust ysize(6) xsize(5.5)) : regress Change_in_sBP_6m Allocation Age Sex CSBP_Baseline Diabetes if Dataset_ID==1 | Dataset_ID==2 ///

| Dataset_ID==4 | Dataset_ID==5 | Dataset_ID==7 | Dataset_ID==8 | Dataset_ID==9 | Dataset_ID==10 | Dataset_ID==11 ///

| Dataset_ID==15 | Dataset_ID==16 | Dataset_ID==17 | Dataset_ID==3 | Dataset_ID==18 | Dataset_ID==191 | Dataset_ID==192 ///

| Dataset_ID==131 | Dataset_ID==132 | Dataset_ID==133 | Dataset_ID==20 | Dataset_ID==121 | Dataset_ID==122 | Dataset_ID==22 | Dataset_ID==23 | Dataset_ID==25

**Control patients for HINTS halved (for level 4 sub-group point estimate)

use "G:\SMART BP IPD\Pooled analysis\BPSMART dataset_Full_split2.dta", clear

set more off

ipdmetan, study(Study) by(level_of_intervention) effect(Mean sBP diff) re lcols((count) Total_population_6m (sum) Control_6m Intervention_6m) ///

forest(graphregion(color(white)) graphregion(margin(zero)) favours(Favours intervention # Favours control) texts(105) astext(60) xlabel(-15(5)10) range(-15,10) ///

noadjust ysize(6) xsize(5.5)) : regress Change_in_sBP_6m Allocation Age Sex CSBP_Baseline Diabetes if Dataset_ID==1 | Dataset_ID==2 ///

| Dataset_ID==4 | Dataset_ID==5 | Dataset_ID==7 | Dataset_ID==8 | Dataset_ID==9 | Dataset_ID==10 | Dataset_ID==11 ///

| Dataset_ID==15 | Dataset_ID==16 | Dataset_ID==17 | Dataset_ID==3 | Dataset_ID==18 | Dataset_ID==191 | Dataset_ID==192 ///

| Dataset_ID==131 | Dataset_ID==132 | Dataset_ID==133 | Dataset_ID==20 | Dataset_ID==121 | Dataset_ID==122 | Dataset_ID==22 | Dataset_ID==23 | Dataset_ID==25

**Control patients for HINTS, TYBC and wakefield split (for summary point estimate)

use "G:\SMART BP IPD\Pooled analysis\BPSMART dataset_Full_split.dta", clear

set more off

ipdmetan, study(Study) by(level_of_intervention) effect(Mean sBP diff) re lcols((count) Total_population_6m (sum) Control_6m Intervention_6m) ///

forest(graphregion(color(white)) graphregion(margin(zero)) favours(Favours intervention # Favours control) texts(105) astext(60) xlabel(-15(5)10) range(-15,10) ///

noadjust ysize(6) xsize(5.5)) : regress Change_in_sBP_6m Allocation Age Sex CSBP_Baseline Diabetes if Dataset_ID==1 | Dataset_ID==2 ///

| Dataset_ID==4 | Dataset_ID==5 | Dataset_ID==7 | Dataset_ID==8 | Dataset_ID==9 | Dataset_ID==10 | Dataset_ID==11 ///

| Dataset_ID==15 | Dataset_ID==16 | Dataset_ID==17 | Dataset_ID==3 | Dataset_ID==18 | Dataset_ID==191 | Dataset_ID==192 ///

| Dataset_ID==131 | Dataset_ID==132 | Dataset_ID==133 | Dataset_ID==20 | Dataset_ID==121 | Dataset_ID==122 | Dataset_ID==22 | Dataset_ID==23 | Dataset_ID==25

*12 months

**Control patients for HINTS complete (for individual study point estimates)

use "G:\SMART BP IPD\Pooled analysis\BPSMART dataset_Full.dta", clear

set more off

ipdmetan, study(Study) by(level_of_intervention) effect(Mean sBP diff) re lcols((count) Total_population_12m (sum) Control_12m Intervention_12m) ///

forest(graphregion(color(white)) graphregion(margin(zero)) favours(Favours intervention # Favours control) texts(105) astext(60) xlabel(-15(5)10) range(-15,10) ///

noadjust ysize(6) xsize(5.5)) : regress Change_in_sBP_12m Allocation Age Sex CSBP_Baseline Diabetes if Dataset_ID==1 | Dataset_ID==2 | Dataset_ID==3 ///

| Dataset_ID==6 | Dataset_ID==5 | Dataset_ID==7| Dataset_ID==9 | Dataset_ID==10 | Dataset_ID==16 | Dataset_ID==141 | Dataset_ID==142 ///

| Dataset_ID==191 | Dataset_ID==192 | Dataset_ID==131 | Dataset_ID==132 | Dataset_ID==133 | Dataset_ID==20 | Dataset_ID==121 | Dataset_ID==122 | Dataset_ID==24

**Control patients for HINTS halved (for level 4 sub-group point estimate)

use "G:\SMART BP IPD\Pooled analysis\BPSMART dataset_Full_split2.dta", clear

set more off

ipdmetan, study(Study) by(level_of_intervention) effect(Mean sBP diff) re lcols((count) Total_population_12m (sum) Control_12m Intervention_12m) ///

forest(graphregion(color(white)) graphregion(margin(zero)) favours(Favours intervention # Favours control) texts(105) astext(60) xlabel(-15(5)10) range(-15,10) ///

noadjust ysize(6) xsize(5.5)) : regress Change_in_sBP_12m Allocation Age Sex CSBP_Baseline Diabetes if Dataset_ID==1 | Dataset_ID==2 | Dataset_ID==3 ///

| Dataset_ID==6 | Dataset_ID==5 | Dataset_ID==7| Dataset_ID==9 | Dataset_ID==10 | Dataset_ID==16 | Dataset_ID==141 | Dataset_ID==142 ///

| Dataset_ID==191 | Dataset_ID==192 | Dataset_ID==131 | Dataset_ID==132 | Dataset_ID==133 | Dataset_ID==20 | Dataset_ID==121 | Dataset_ID==122 | Dataset_ID==24

**Control patients for HINTS, TYBC and wakefield split (for summary point estimate)

use "G:\SMART BP IPD\Pooled analysis\BPSMART dataset_Full_split.dta", clear

set more off

ipdmetan, study(Study) by(level_of_intervention) effect(Mean sBP diff) re lcols((count) Total_population_12m (sum) Control_12m Intervention_12m) ///

forest(graphregion(color(white)) graphregion(margin(zero)) favours(Favours intervention # Favours control) texts(105) astext(60) xlabel(-15(5)10) range(-15,10) ///

noadjust ysize(6) xsize(5.5)) : regress Change_in_sBP_12m Allocation Age Sex CSBP_Baseline Diabetes if Dataset_ID==1 | Dataset_ID==2 | Dataset_ID==3 ///

| Dataset_ID==6 | Dataset_ID==5 | Dataset_ID==7| Dataset_ID==9 | Dataset_ID==10 | Dataset_ID==16 | Dataset_ID==141 | Dataset_ID==142 ///

| Dataset_ID==191 | Dataset_ID==192 | Dataset_ID==131 | Dataset_ID==132 | Dataset_ID==133 | Dataset_ID==20 | Dataset_ID==121 | Dataset_ID==122 | Dataset_ID==24

*18 months

**Control patients for HINTS complete (for individual study point estimates)

use "G:\SMART BP IPD\Pooled analysis\BPSMART dataset_Full.dta", clear

set more off

ipdmetan, study(Study) by(level_of_intervention) effect(Mean sBP diff) re lcols((count) Total_population_18m (sum) Control_18m Intervention_18m) ///

forest(graphregion(color(white)) graphregion(margin(zero)) favours(Favours intervention # Favours control) texts(105) astext(60) xlabel(-15(5)10) range(-15,10) ///

noadjust ysize(6) xsize(5.5)) : regress Change_in_sBP_18m Allocation Age Sex CSBP_Baseline Diabetes if Dataset_ID==9 | Dataset_ID==16 ///

| Dataset_ID==131 | Dataset_ID==132 | Dataset_ID==133 | Dataset_ID==121 | Dataset_ID==122 | Dataset_ID==211 | Dataset_ID==212

**Control patients for HINTS halved (for level 4 sub-group point estimate)

use "G:\SMART BP IPD\Pooled analysis\BPSMART dataset_Full_split2.dta", clear

set more off

ipdmetan, study(Study) by(level_of_intervention) effect(Mean sBP diff) re lcols((count) Total_population_18m (sum) Control_18m Intervention_18m) ///

forest(graphregion(color(white)) graphregion(margin(zero)) favours(Favours intervention # Favours control) texts(105) astext(60) xlabel(-15(5)10) range(-15,10) ///

noadjust ysize(6) xsize(5.5)) : regress Change_in_sBP_18m Allocation Age Sex CSBP_Baseline Diabetes if Dataset_ID==9 | Dataset_ID==16 ///

| Dataset_ID==131 | Dataset_ID==132 | Dataset_ID==133 | Dataset_ID==121 | Dataset_ID==122 | Dataset_ID==211 | Dataset_ID==212

**Control patients for HINTS, TYBC and wakefield split (for summary point estimate)

use "G:\SMART BP IPD\Pooled analysis\BPSMART dataset_Full_split.dta", clear

set more off

ipdmetan, study(Study) by(level_of_intervention) effect(Mean sBP diff) re lcols((count) Total_population_18m (sum) Control_18m Intervention_18m) ///

forest(graphregion(color(white)) graphregion(margin(zero)) favours(Favours intervention # Favours control) texts(105) astext(60) xlabel(-15(5)10) range(-15,10) ///

noadjust ysize(6) xsize(5.5)) : regress Change_in_sBP_18m Allocation Age Sex CSBP_Baseline Diabetes if Dataset_ID==9 | Dataset_ID==16 ///

| Dataset_ID==131 | Dataset_ID==132 | Dataset_ID==133 | Dataset_ID==121 | Dataset_ID==122 | Dataset_ID==211 | Dataset_ID==212

*****************Diastolic Blood pressure**********************

*****IPD mean change in dBP by study and level of intevention, adjusted by age, sex and baseline BP; ************

*6 months

**Control patients for HINTS complete (for individual study point estimates)

set more off

ipdmetan, study(Study) by(level_of_intervention) effect(Mean dBP diff) re lcols((count) Total_population_6m (sum) Control_6m Intervention_6m) ///

forest(graphregion(color(white)) graphregion(margin(zero)) favours(Favours intervention # Favours control) texts(105) astext(60) xlabel(-10(5)5) range(-10,5) ///

noadjust ysize(6) xsize(5.5)) : regress Change_in_dBP_6m Allocation Age Sex CDBP_Baseline Diabetes if Dataset_ID==1 | Dataset_ID==2 ///

| Dataset_ID==4 | Dataset_ID==5 | Dataset_ID==7 | Dataset_ID==8 | Dataset_ID==9 | Dataset_ID==10 | Dataset_ID==11 ///

| Dataset_ID==15 | Dataset_ID==16 | Dataset_ID==17 | Dataset_ID==3 | Dataset_ID==18 | Dataset_ID==191 | Dataset_ID==192 ///

| Dataset_ID==131 | Dataset_ID==132 | Dataset_ID==133 | Dataset_ID==20 | Dataset_ID==121 | Dataset_ID==122 | Dataset_ID==22 | Dataset_ID==23 | Dataset_ID==25

**Control patients for HINTS halved (for level 4 sub-group point estimate)

use "G:\SMART BP IPD\Pooled analysis\BPSMART dataset_Full_split2.dta", clear

set more off

ipdmetan, study(Study) by(level_of_intervention) effect(Mean dBP diff) re lcols((count) Total_population_6m (sum) Control_6m Intervention_6m) ///

forest(graphregion(color(white)) graphregion(margin(zero)) favours(Favours intervention # Favours control) texts(105) astext(60) xlabel(-10(5)5) range(-10,5) ///

noadjust ysize(6) xsize(5.5)) : regress Change_in_dBP_6m Allocation Age Sex CDBP_Baseline Diabetes if Dataset_ID==1 | Dataset_ID==2 ///

| Dataset_ID==4 | Dataset_ID==5 | Dataset_ID==7 | Dataset_ID==8 | Dataset_ID==9 | Dataset_ID==10 | Dataset_ID==11 ///

| Dataset_ID==15 | Dataset_ID==16 | Dataset_ID==17 | Dataset_ID==3 | Dataset_ID==18 | Dataset_ID==191 | Dataset_ID==192 ///

| Dataset_ID==131 | Dataset_ID==132 | Dataset_ID==133 | Dataset_ID==20 | Dataset_ID==121 | Dataset_ID==122 | Dataset_ID==22 | Dataset_ID==23 | Dataset_ID==25

**Control patients for HINTS, TYBC and wakefield split (for summary point estimate)

use "G:\SMART BP IPD\Pooled analysis\BPSMART dataset_Full_split.dta", clear

set more off

ipdmetan, study(Study) by(level_of_intervention) effect(Mean dBP diff) re lcols((count) Total_population_6m (sum) Control_6m Intervention_6m) ///

forest(graphregion(color(white)) graphregion(margin(zero)) favours(Favours intervention # Favours control) texts(105) astext(60) xlabel(-10(5)5) range(-10,5) ///

noadjust ysize(6) xsize(5.5)) : regress Change_in_dBP_6m Allocation Age Sex CDBP_Baseline Diabetes if Dataset_ID==1 | Dataset_ID==2 ///

| Dataset_ID==4 | Dataset_ID==5 | Dataset_ID==7 | Dataset_ID==8 | Dataset_ID==9 | Dataset_ID==10 | Dataset_ID==11 ///

| Dataset_ID==15 | Dataset_ID==16 | Dataset_ID==17 | Dataset_ID==3 | Dataset_ID==18 | Dataset_ID==191 | Dataset_ID==192 ///

| Dataset_ID==131 | Dataset_ID==132 | Dataset_ID==133 | Dataset_ID==20 | Dataset_ID==121 | Dataset_ID==122 | Dataset_ID==22 | Dataset_ID==23 | Dataset_ID==25

*12 months

**Control patients for HINTS complete (for individual study point estimates)

use "G:\SMART BP IPD\Pooled analysis\BPSMART dataset_Full.dta", clear

set more off

ipdmetan, study(Study) by(level_of_intervention) effect(Mean dBP diff) re lcols((count) Total_population_12m (sum) Control_12m Intervention_12m) ///

forest(graphregion(color(white)) graphregion(margin(zero)) favours(Favours intervention # Favours control) texts(105) astext(60) xlabel(-10(5)5) range(-10,5) ///

noadjust ysize(6) xsize(5.5)) : regress Change_in_dBP_12m Allocation Age Sex CDBP_Baseline Diabetes if Dataset_ID==1 | Dataset_ID==2 | Dataset_ID==3 ///

| Dataset_ID==6 | Dataset_ID==5 | Dataset_ID==7| Dataset_ID==9 | Dataset_ID==10 | Dataset_ID==16 | Dataset_ID==141 | Dataset_ID==142 ///

| Dataset_ID==191 | Dataset_ID==192 | Dataset_ID==131 | Dataset_ID==132 | Dataset_ID==133 | Dataset_ID==20 | Dataset_ID==121 | Dataset_ID==122 | Dataset_ID==24

**Control patients for HINTS halved (for level 4 sub-group point estimate)

use "G:\SMART BP IPD\Pooled analysis\BPSMART dataset_Full_split2.dta", clear

set more off

ipdmetan, study(Study) by(level_of_intervention) effect(Mean dBP diff) re lcols((count) Total_population_12m (sum) Control_12m Intervention_12m) ///

forest(graphregion(color(white)) graphregion(margin(zero)) favours(Favours intervention # Favours control) texts(105) astext(60) xlabel(-10(5)5) range(-10,5) ///

noadjust ysize(6) xsize(5.5)) : regress Change_in_dBP_12m Allocation Age Sex CDBP_Baseline Diabetes if Dataset_ID==1 | Dataset_ID==2 | Dataset_ID==3 ///

| Dataset_ID==6 | Dataset_ID==5 | Dataset_ID==7| Dataset_ID==9 | Dataset_ID==10 | Dataset_ID==16 | Dataset_ID==141 | Dataset_ID==142 ///

| Dataset_ID==191 | Dataset_ID==192 | Dataset_ID==131 | Dataset_ID==132 | Dataset_ID==133 | Dataset_ID==20 | Dataset_ID==121 | Dataset_ID==122 | Dataset_ID==24

**Control patients for HINTS, TYBC and wakefield split (for summary point estimate)

use "G:\SMART BP IPD\Pooled analysis\BPSMART dataset_Full_split.dta", clear

set more off

ipdmetan, study(Study) by(level_of_intervention) effect(Mean dBP diff) re lcols((count) Total_population_12m (sum) Control_12m Intervention_12m) ///

forest(graphregion(color(white)) graphregion(margin(zero)) favours(Favours intervention # Favours control) texts(105) astext(60) xlabel(-10(5)5) range(-10,5) ///

noadjust ysize(6) xsize(5.5)) : regress Change_in_dBP_12m Allocation Age Sex CDBP_Baseline Diabetes if Dataset_ID==1 | Dataset_ID==2 | Dataset_ID==3 ///

| Dataset_ID==6 | Dataset_ID==5 | Dataset_ID==7| Dataset_ID==9 | Dataset_ID==10 | Dataset_ID==16 | Dataset_ID==141 | Dataset_ID==142 ///

| Dataset_ID==191 | Dataset_ID==192 | Dataset_ID==131 | Dataset_ID==132 | Dataset_ID==133 | Dataset_ID==20 | Dataset_ID==121 | Dataset_ID==122 | Dataset_ID==24

*18 months

**Control patients for HINTS complete (for individual study point estimates)

use "G:\SMART BP IPD\Pooled analysis\BPSMART dataset_Full.dta", clear

set more off

ipdmetan, study(Study) by(level_of_intervention) effect(Mean dBP diff) re lcols((count) Total_population_18m (sum) Control_18m Intervention_18m) ///

forest(graphregion(color(white)) graphregion(margin(zero)) favours(Favours intervention # Favours control) texts(105) astext(60) xlabel(-10(5)5) range(-10,5) ///

noadjust ysize(6) xsize(5.5)) : regress Change_in_dBP_18m Allocation Age Sex CSBP_Baseline Diabetes if Dataset_ID==9 | Dataset_ID==16 ///

| Dataset_ID==131 | Dataset_ID==132 | Dataset_ID==133 | Dataset_ID==121 | Dataset_ID==122 | Dataset_ID==211 | Dataset_ID==212

**Control patients for HINTS halved (for level 4 sub-group point estimate)

use "G:\SMART BP IPD\Pooled analysis\BPSMART dataset_Full_split2.dta", clear

set more off

ipdmetan, study(Study) by(level_of_intervention) effect(Mean dBP diff) re lcols((count) Total_population_18m (sum) Control_18m Intervention_18m) ///

forest(graphregion(color(white)) graphregion(margin(zero)) favours(Favours intervention # Favours control) texts(105) astext(60) xlabel(-10(5)5) range(-10,5) ///

noadjust ysize(6) xsize(5.5)) : regress Change_in_dBP_18m Allocation Age Sex CSBP_Baseline Diabetes if Dataset_ID==9 | Dataset_ID==16 ///

| Dataset_ID==131 | Dataset_ID==132 | Dataset_ID==133 | Dataset_ID==121 | Dataset_ID==122 | Dataset_ID==211 | Dataset_ID==212

**Control patients for HINTS, TYBC and wakefield split (for summary point estimate)

use "G:\SMART BP IPD\Pooled analysis\BPSMART dataset_Full_split.dta", clear

set more off

ipdmetan, study(Study) by(level_of_intervention) effect(Mean sBP diff) re lcols((count) Total_population_18m (sum) Control_18m Intervention_18m) ///

forest(graphregion(color(white)) graphregion(margin(zero)) favours(Favours intervention # Favours control) texts(105) astext(60) xlabel(-10(5)5) range(-10,5) ///

noadjust ysize(6) xsize(5.5)) : regress Change_in_dBP_18m Allocation Age Sex CSBP_Baseline Diabetes if Dataset_ID==9 | Dataset_ID==16 ///

| Dataset_ID==131 | Dataset_ID==132 | Dataset_ID==133 | Dataset_ID==121 | Dataset_ID==122 | Dataset_ID==211 | Dataset_ID==212

***************Relative risk of blood pressure (systolic or diastolic) control******************

***RR of BP control by study and level of intevention, adjusted by age, sex and baseline BP**********

*6 months

**Control patients for HINTS complete (for individual study point estimates)

use "G:\SMART BP IPD\Pooled analysis\BPSMART dataset_Full.dta", clear

set more off

recode BP_control_6m (1=2)

recode BP_control_6m (0=1)

recode BP_control_6m (2=0)

ipdmetan, study(Study) by(level_of_intervention) effect(RR of BP control) lcols((count) Total_population_6m (sum) Control_6m Intervention_6m) ///

forest(graphregion(color(white)) graphregion(margin(zero)) favours(Favours intervention # Favours control) texts(100) astext(60) xlabel(0.5(1)2.5) range(0.1,2.5) ///

noadjust ysize(6.95) xsize(6)) rrr re: mlogit BP_control_6m Allocation Age Sex CSBP_Baseline CDBP_Baseline Diabetes ///

if Dataset_ID==1 | Dataset_ID==2 | Dataset_ID==3 | Dataset_ID==131 | Dataset_ID==132 | Dataset_ID==133 ///

| Dataset_ID==4 | Dataset_ID==5 | Dataset_ID==7 | Dataset_ID==8 | Dataset_ID==9 | Dataset_ID==10 | Dataset_ID==11 ///

| Dataset_ID==15 | Dataset_ID==16 | Dataset_ID==17 | Dataset_ID==18 | Dataset_ID==191 | Dataset_ID==192 | Dataset_ID==20 | Dataset_ID==121 | Dataset_ID==122 ///

| Dataset_ID==22 | Dataset_ID==23 | Dataset_ID==25, baseoutcome(0)

**Control patients for HINTS halved (for level 4 sub-group point estimate)

use "G:\SMART BP IPD\Pooled analysis\BPSMART dataset_Full_split2.dta", clear

set more off

recode BP_control_6m (1=2)

recode BP_control_6m (0=1)

recode BP_control_6m (2=0)

ipdmetan, study(Study) by(level_of_intervention) effect(RR of BP control) lcols((count) Total_population_6m (sum) Control_6m Intervention_6m) ///

forest(graphregion(color(white)) graphregion(margin(zero)) favours(Favours intervention # Favours control) texts(100) astext(60) xlabel(0.5(1)2.5) range(0.1,2.5) ///

noadjust ysize(6.95) xsize(6)) rrr re: mlogit BP_control_6m Allocation Age Sex CSBP_Baseline CDBP_Baseline Diabetes ///

if Dataset_ID==1 | Dataset_ID==2 | Dataset_ID==3 | Dataset_ID==131 | Dataset_ID==132 | Dataset_ID==133 ///

| Dataset_ID==4 | Dataset_ID==5 | Dataset_ID==7 | Dataset_ID==8 | Dataset_ID==9 | Dataset_ID==10 | Dataset_ID==11 ///

| Dataset_ID==15 | Dataset_ID==16 | Dataset_ID==17 | Dataset_ID==18 | Dataset_ID==191 | Dataset_ID==192 | Dataset_ID==20 | Dataset_ID==121 | Dataset_ID==122 ///

| Dataset_ID==22 | Dataset_ID==23 | Dataset_ID==25, baseoutcome(0)

**Control patients for HINTS, TYBC and wakefield split (for summary point estimate)

use "G:\SMART BP IPD\Pooled analysis\BPSMART dataset_Full_split.dta", clear

set more off

recode BP_control_6m (1=2)

recode BP_control_6m (0=1)

recode BP_control_6m (2=0)

ipdmetan, study(Study) by(level_of_intervention) effect(RR of BP control) lcols((count) Total_population_6m (sum) Control_6m Intervention_6m) ///

forest(graphregion(color(white)) graphregion(margin(zero)) favours(Favours intervention # Favours control) texts(100) astext(60) xlabel(0.5(1)2.5) range(0.1,2.5) ///

noadjust ysize(6.95) xsize(6)) rrr re: mlogit BP_control_6m Allocation Age Sex CSBP_Baseline CDBP_Baseline Diabetes ///

if Dataset_ID==1 | Dataset_ID==2 | Dataset_ID==3 | Dataset_ID==131 | Dataset_ID==132 | Dataset_ID==133 ///

| Dataset_ID==4 | Dataset_ID==5 | Dataset_ID==7 | Dataset_ID==8 | Dataset_ID==9 | Dataset_ID==10 | Dataset_ID==11 ///

| Dataset_ID==15 | Dataset_ID==16 | Dataset_ID==17 | Dataset_ID==18 | Dataset_ID==191 | Dataset_ID==192 | Dataset_ID==20 | Dataset_ID==121 | Dataset_ID==122

| Dataset_ID==22 | Dataset_ID==23 | Dataset_ID==25, baseoutcome(0)

*12 months

**Control patients for HINTS complete (for individual study point estimates)

use "G:\SMART BP IPD\Pooled analysis\BPSMART dataset_Full.dta", clear

set more off

recode BP_control_12m (1=2)

recode BP_control_12m (0=1)

recode BP_control_12m (2=0)

ipdmetan, study(Study) by(level_of_intervention) effect(RR of BP control) lcols((count) Total_population_12m (sum) Control_12m Intervention_12m) ///

forest(graphregion(color(white)) graphregion(margin(zero)) favours(Favours intervention # Favours control) texts(100) astext(60) xlabel(0.5(1)2.5) range(0.1,2.5) ///

noadjust ysize(6.95) xsize(6)) rrr re: mlogit BP_control_12m Allocation Age Sex CSBP_Baseline CDBP_Baseline Diabetes if Dataset_ID==1 | Dataset_ID==2 | Dataset_ID==3 ///

| Dataset_ID==6 | Dataset_ID==5 | Dataset_ID==7| Dataset_ID==9 | Dataset_ID==10 | Dataset_ID==16 | Dataset_ID==141 | Dataset_ID==142 ///

| Dataset_ID==191 | Dataset_ID==192 | Dataset_ID==131 | Dataset_ID==132 | Dataset_ID==133 | Dataset_ID==20 | Dataset_ID==121 | Dataset_ID==122 | Dataset_ID==24, baseoutcome(0)

**Control patients for HINTS halved (for level 4 sub-group point estimate)

use "G:\SMART BP IPD\Pooled analysis\BPSMART dataset_Full_split2.dta", clear

set more off

recode BP_control_12m (1=2)

recode BP_control_12m (0=1)

recode BP_control_12m (2=0)

ipdmetan, study(Study) by(level_of_intervention) effect(RR of BP control) lcols((count) Total_population_12m (sum) Control_12m Intervention_12m) ///

forest(graphregion(color(white)) graphregion(margin(zero)) favours(Favours intervention # Favours control) texts(100) astext(60) xlabel(0.5(1)2.5) range(0.1,2.5) ///

noadjust ysize(6.95) xsize(6)) rrr re: mlogit BP_control_12m Allocation Age Sex CSBP_Baseline CDBP_Baseline Diabetes if Dataset_ID==1 | Dataset_ID==2 | Dataset_ID==3 ///

| Dataset_ID==6 | Dataset_ID==5 | Dataset_ID==7| Dataset_ID==9 | Dataset_ID==10 | Dataset_ID==16 | Dataset_ID==141 | Dataset_ID==142 ///

| Dataset_ID==191 | Dataset_ID==192 | Dataset_ID==131 | Dataset_ID==132 | Dataset_ID==133 | Dataset_ID==20 | Dataset_ID==121 | Dataset_ID==122 | Dataset_ID==24, baseoutcome(0)

**Control patients for HINTS, TYBC and wakefield split (for summary point estimate)

use "G:\SMART BP IPD\Pooled analysis\BPSMART dataset_Full_split.dta", clear

set more off

recode BP_control_12m (1=2)

recode BP_control_12m (0=1)

recode BP_control_12m (2=0)

ipdmetan, study(Study) by(level_of_intervention) effect(RR of BP control) lcols((count) Total_population_12m (sum) Control_12m Intervention_12m) ///

forest(graphregion(color(white)) graphregion(margin(zero)) favours(Favours intervention # Favours control) texts(100) astext(60) xlabel(0.5(1)2.5) range(0.1,2.5) ///

noadjust ysize(6.95) xsize(6)) rrr re: mlogit BP_control_12m Allocation Age Sex CSBP_Baseline CDBP_Baseline Diabetes if Dataset_ID==1 | Dataset_ID==2 | Dataset_ID==3 ///

| Dataset_ID==6 | Dataset_ID==5 | Dataset_ID==7| Dataset_ID==9 | Dataset_ID==10 | Dataset_ID==16 | Dataset_ID==141 | Dataset_ID==142 ///

| Dataset_ID==191 | Dataset_ID==192 | Dataset_ID==131 | Dataset_ID==132 | Dataset_ID==133 | Dataset_ID==20 | Dataset_ID==121 | Dataset_ID==122 | Dataset_ID==24, baseoutcome(0)

*18 months

**Control patients for HINTS complete (for individual study point estimates)

use "G:\SMART BP IPD\Pooled analysis\BPSMART dataset_Full.dta", clear

set more off

recode BP_control_18m (1=2)

recode BP_control_18m (0=1)

recode BP_control_18m (2=0)

ipdmetan, study(Study) by(level_of_intervention) effect(RR of BP control) lcols((count) Total_population_18m (sum) Control_18m Intervention_18m) ///

forest(graphregion(color(white)) graphregion(margin(zero)) favours(Favours intervention # Favours control) texts(100) astext(60) xlabel(0.5(1)2.5) range(0.1,2.5) ///

noadjust ysize(6.95) xsize(6)) rrr re: mlogit BP_control_18m Allocation Age Sex CSBP_Baseline CDBP_Baseline Diabetes if Dataset_ID==9 | Dataset_ID==16 ///

| Dataset_ID==131 | Dataset_ID==132 | Dataset_ID==133 | Dataset_ID==121 | Dataset_ID==122 | Dataset_ID==211 | Dataset_ID==212, baseoutcome(0)

**Control patients for HINTS halved (for level 4 sub-group point estimate)

use "G:\SMART BP IPD\Pooled analysis\BPSMART dataset_Full_split2.dta", clear

set more off

recode BP_control_18m (1=2)

recode BP_control_18m (0=1)

recode BP_control_18m (2=0)

ipdmetan, study(Study) by(level_of_intervention) effect(RR of BP control) lcols((count) Total_population_18m (sum) Control_18m Intervention_18m) ///

forest(graphregion(color(white)) graphregion(margin(zero)) favours(Favours intervention # Favours control) texts(100) astext(60) xlabel(0.5(1)2.5) range(0.1,2.5) ///

noadjust ysize(6.95) xsize(6)) rrr re: mlogit BP_control_18m Allocation Age Sex CSBP_Baseline CDBP_Baseline Diabetes if Dataset_ID==9 | Dataset_ID==16 ///

| Dataset_ID==131 | Dataset_ID==132 | Dataset_ID==133 | Dataset_ID==121 | Dataset_ID==122 | Dataset_ID==211 | Dataset_ID==212, baseoutcome(0)

**Control patients for HINTS, TYBC and wakefield split (for summary point estimate)

use "G:\SMART BP IPD\Pooled analysis\BPSMART dataset_Full_split.dta", clear

set more off

recode BP_control_18m (1=2)

recode BP_control_18m (0=1)

recode BP_control_18m (2=0)

ipdmetan, study(Study) by(level_of_intervention) effect(RR of BP control) lcols((count) Total_population_18m (sum) Control_18m Intervention_18m) ///

forest(graphregion(color(white)) graphregion(margin(zero)) favours(Favours intervention # Favours control) texts(100) astext(60) xlabel(0.5(1)2.5) range(0.1,2.5) ///

noadjust ysize(6.95) xsize(6)) rrr re: mlogit BP_control_18m Allocation Age Sex CSBP_Baseline CDBP_Baseline Diabetes if Dataset_ID==9 | Dataset_ID==16 ///

| Dataset_ID==131 | Dataset_ID==132 | Dataset_ID==133 | Dataset_ID==121 | Dataset_ID==122 | Dataset_ID==211 | Dataset_ID==212, baseoutcome(0)

**********ABPM************

****Calculate change in Blood pressure at follow-up***

gen Change_in_AsBPM_6m = ABPM_Daytime_sys_6months-ABPM_Daytime_sys_baseline

gen Change_in_AdBPM_6m = ABPM_Daytime_dia_6months-ABPM_Daytime_dia_baseline

gen Change_in_AsBPM_12m = ABPM_Daytime_sys_12months-ABPM_Daytime_sys_baseline

gen Change_in_AdBPM_12m = ABPM_Daytime_dia_12months-ABPM_Daytime_dia_baseline

*****IPD mean change in sBP at follow-up by study, adjusted by age, sex and baseline BP************

*Clinic 6 mths

set more off

ipdmetan, study(Study) effect(Mean sBP diff) re lcols((count) Total_population_6m (sum) Control_6m Intervention_6m) forest(graphregion(color(white)) ///

graphregion(margin(zero)) favours(Favours intervention # Favours control) texts(105) astext(60) xlabel(-10(5)5) range(-10,5.5) noadjust ysize(3) xsize(6)) ///

: regress Change_in_sBP_6m Allocation Age Sex CSBP_Baseline Diabetes level_of_intervention if Dataset==4|Dataset==5| Dataset==7 | Dataset==8 | Dataset==9

*Ambulatory 6 mths

set more off

ipdmetan, study(Study) effect(Mean sBP diff) re lcols((count) Total_population_6m (sum) Control_6m Intervention_6m) forest(graphregion(color(white)) ///

graphregion(margin(zero)) favours(Favours intervention # Favours control) texts(105) astext(60) xlabel(-10(5)5) range(-10,5.5) noadjust ysize(3) xsize(6)) ///

: regress Change_in_AsBPM_6m Allocation Age Sex CSBP_Baseline Diabetes level_of_intervention if Dataset==4|Dataset==5| Dataset==7 | Dataset==8 | Dataset==9

*Clinic 12 mths

set more off

ipdmetan, study(Study) effect(Mean sBP diff) re lcols((count) Total_population_12m (sum) Control_12m Intervention_12m) forest(graphregion(color(white)) ///

graphregion(margin(zero)) favours(Favours intervention # Favours control) texts(105) astext(60) xlabel(-10(5)5) range(-10,5.5) noadjust ysize(3) xsize(6)) ///

: regress Change_in_sBP_12m Allocation Age Sex CSBP_Baseline Diabetes level_of_intervention if Dataset==6|Dataset==5| Dataset==7 | Dataset==9

*Ambulatory 12 mths

set more off

ipdmetan, study(Study) effect(Mean sBP diff) re lcols((count) Total_population_12m (sum) Control_12m Intervention_12m) forest(graphregion(color(white)) ///

graphregion(margin(zero)) favours(Favours intervention # Favours control) texts(105) astext(60) xlabel(-10(5)5) range(-10,5.5) noadjust ysize(3) xsize(6)) ///

: regress Change_in_AsBPM_12m Allocation Age Sex CSBP_Baseline Diabetes level_of_intervention if Dataset==6|Dataset==5| Dataset==7| Dataset==9

*****IPD mean change in dBP at follow-up by study, adjusted by age, sex and baseline BP************

*Clinic 6 mths

set more off

ipdmetan, study(Study) effect(Mean dBP diff) re lcols((count) Total_population_6m (sum) Control_6m Intervention_6m) forest(graphregion(color(white)) ///

graphregion(margin(zero)) favours(Favours intervention # Favours control) texts(105) astext(60) xlabel(-5(2.5)5) range(-5.5,5.5) noadjust ysize(3) xsize(6)) ///

: regress Change_in_dBP_6m Allocation Age Sex CDBP_Baseline Diabetes level_of_intervention if Dataset==4|Dataset==5| Dataset==7 | Dataset==8 | Dataset==9

*Ambulatory 6 mths

set more off

ipdmetan, study(Study) effect(Mean dBP diff) re lcols((count) Total_population_6m (sum) Control_6m Intervention_6m) forest(graphregion(color(white)) ///

graphregion(margin(zero)) favours(Favours intervention # Favours control) texts(105) astext(60) xlabel(-5(2.5)5) range(-5.5,5.5) noadjust ysize(3) xsize(6)) ///

: regress Change_in_AdBPM_6m Allocation Age Sex CDBP_Baseline Diabetes level_of_intervention if Dataset==4|Dataset==5| Dataset==7 | Dataset==8 | Dataset==9

*Clinic 12 mths

set more off

ipdmetan, study(Study) effect(Mean dBP diff) re lcols((count) Total_population_12m (sum) Control_12m Intervention_12m) forest(graphregion(color(white)) ///

graphregion(margin(zero)) favours(Favours intervention # Favours control) texts(105) astext(60) xlabel(-5(2.5)5) range(-5.5,5.5) noadjust ysize(3) xsize(6)) ///

: regress Change_in_dBP_12m Allocation Age Sex CDBP_Baseline Diabetes level_of_intervention if Dataset==6|Dataset==5| Dataset==7 | Dataset==9

*Ambulatory 12 mths

set more off

ipdmetan, study(Study) effect(Mean sBP diff) re lcols((count) Total_population_12m (sum) Control_12m Intervention_12m) forest(graphregion(color(white)) ///

graphregion(margin(zero)) favours(Favours intervention # Favours control) texts(105) astext(60) xlabel(-5(2.5)5) range(-5.5,5.5) noadjust ysize(3) xsize(6)) ///

: regress Change_in_AdBPM_12m Allocation Age Sex CDBP_Baseline Diabetes level_of_intervention if Dataset==6|Dataset==5| Dataset==7 | Dataset==9

*******Sub-group analyses*******

*****IPD mean change in sBP at 12months by age, adjusted by sex, and baseline BP and level of intervention************

use "G:\SMART BP IPD\Pooled analysis\BPSMART dataset_subgroup.dta", clear

set more off

ipdmetan, study(Subgroup_category) by(Subgroup) effect(Mean sBP diff) re lcols(Total_studies (count) Complete_data (sum) Complete_data_Con Complete_data_Int ) ///

forest(graphregion(color(white)) graphregion(margin(zero)) favours(Favours intervention # Favours control) texts(105) astext(60) xlabel(-15(5)5) range(-13,5) boxsca(50) ///

ysize(6) xsize(5.5) boxopts(msymbol(D))) nooverall nosubgroup: regress Change_in_sBP_12m Allocation Age Sex CSBP_Baseline i.level_of_intervention i.study_variable ///

if Dataset_ID==1 | Dataset_ID==2 | Dataset_ID==3 | Dataset_ID==6 | Dataset_ID==5 | Dataset_ID==7| Dataset_ID==9 | Dataset_ID==10 | Dataset_ID==16 ///

| Dataset_ID==121 | Dataset_ID==122 | Dataset_ID==141 | Dataset_ID==142 | Dataset_ID==191 | Dataset_ID==192 | Dataset_ID==131 ///

| Dataset_ID==132 | Dataset_ID==133 | Dataset_ID==20

*****IPD mean change in dBP at 12months by age, adjusted by sex, and baseline BP and level of intervention************

use "G:\SMART BP IPD\Pooled analysis\BPSMART dataset_subgroup.dta", clear

set more off

ipdmetan, study(Subgroup_category) by(Subgroup) effect(Mean dBP diff) re lcols(Total_studies (count) Complete_data (sum) Complete_data_Con Complete_data_Int ) ///

forest(graphregion(color(white)) graphregion(margin(zero)) favours(Favours intervention # Favours control) texts(105) astext(60) xlabel(-10(5)5) range(-10,5) boxsca(50) ///

ysize(6) xsize(5.5) boxopts(msymbol(D))) nooverall nosubgroup : regress Change_in_dBP_12m Allocation Age Sex CDBP_Baseline i.level_of_intervention i.study_variable ///

if Dataset_ID==1 | Dataset_ID==2 | Dataset_ID==3 | Dataset_ID==6 | Dataset_ID==5 | Dataset_ID==7| Dataset_ID==9 | Dataset_ID==10 | Dataset_ID==16 ///

| Dataset_ID==121 | Dataset_ID==122 | Dataset_ID==141 | Dataset_ID==142 | Dataset_ID==191 | Dataset_ID==192 | Dataset_ID==131 ///

| Dataset_ID==132 | Dataset_ID==133 | Dataset_ID==20

***RR of BP control by study, adjusted by age, sex, Diabetes and baseline BP**********

use "G:\SMART BP IPD\Pooled analysis\BPSMART dataset_subgroup.dta", clear

set more off

recode BP_control_12m (1=2)

recode BP_control_12m (0=1)

recode BP_control_12m (2=0)

ipdmetan, study(Subgroup_category) by(Subgroup) effect(RR of BP control) re lcols(Total_studies (count) Complete_data (sum) Complete_data_Con Complete_data_Int) ///

forest(graphregion(color(white)) graphregion(margin(zero)) favours(Favours intervention # Favours control) texts(100) astext(60) xlabel(0.5(0.5)1.5) range(0.1,2) boxsca(35) ///

ysize(6) xsize(5.5) boxopts(msymbol(D))) nooverall nosubgroup rrr : mlogit BP_control_12m Allocation Age Sex CSBP_Baseline CDBP_Baseline i.level_of_intervention i.study_variable ///

if Dataset_ID==1 | Dataset_ID==2 | Dataset_ID==3 | Dataset_ID==6 | Dataset_ID==5 | Dataset_ID==7| Dataset_ID==9 | Dataset_ID==10 | Dataset_ID==16 ///

| Dataset_ID==121 | Dataset_ID==122 | Dataset_ID==141 | Dataset_ID==142 | Dataset_ID==191 | Dataset_ID==192 | Dataset_ID==131 ///

| Dataset_ID==132 | Dataset_ID==133 | Dataset_ID==20, baseoutcome(0)
